# Blood Pressure Sensors: Materials, Fabrication Methods, Performance Evaluations and Future Perspectives

**DOI:** 10.3390/s20164484

**Published:** 2020-08-11

**Authors:** Ahmed Al-Qatatsheh, Yosry Morsi, Ali Zavabeti, Ali Zolfagharian, Nisa Salim, Abbas Z. Kouzani, Bobak Mosadegh, Saleh Gharaie

**Affiliations:** 1 Faculty of Science, Engineering, and Technology (FSET), Swinburne University of Technology, Melbourne VIC 3122, Australia; Ymorsi@swin.edu.au (Y.M.); nsalim@swin.edu.au (N.S.); 2 Department of Chemical Engineering, The University of Melbourne, Parkville VIC 3010, Australia; ali.zavabeti@unimelb.edu.au; 3 Faculty of Science, Engineering and Built Environment, School of Engineering, Deakin University, Waurn Ponds VIC 3216, Australia; a.zolfagharian@deakin.edu.au (A.Z.); abbas.kouzani@deakin.edu.au (A.Z.K.); 4 Dalio Institute of Cardiovascular Imaging, Weill Cornell Medicine, New York, NY 10065, USA; bom2008@med.cornell.edu

**Keywords:** wearable sensors, sensing materials, smart health monitor devices, sensor operational lifecycle

## Abstract

Advancements in materials science and fabrication techniques have contributed to the significant growing attention to a wide variety of sensors for digital healthcare. While the progress in this area is tremendously impressive, few wearable sensors with the capability of real-time blood pressure monitoring are approved for clinical use. One of the key obstacles in the further development of wearable sensors for medical applications is the lack of comprehensive technical evaluation of sensor materials against the expected clinical performance. Here, we present an extensive review and critical analysis of various materials applied in the design and fabrication of wearable sensors. In our unique transdisciplinary approach, we studied the fundamentals of blood pressure and examined its measuring modalities while focusing on their clinical use and sensing principles to identify material functionalities. Then, we carefully reviewed various categories of functional materials utilized in sensor building blocks allowing for comparative analysis of the performance of a wide range of materials throughout the sensor operational-life cycle. Not only this provides essential data to enhance the materials’ properties and optimize their performance, but also, it highlights new perspectives and provides suggestions to develop the next generation pressure sensors for clinical use.

## 1. Introduction

Cardiovascular diseases caused 31% of deaths worldwide [1], and recently, they had the highest confirmed death cases in Italy and China during the novel pandemic known as the coronavirus disease 2019 (COVID-19) [2,3]. In return, the demand for an accurate home-diagnostic tool for blood pressure measurements, along with other vital signs (e.g., temperature, respiratory rate) has increased massively. These tools, especially if enabled with telemedicine, will not only help assess a patient’s health status, triage the patient to appropriate care, determine potential diagnoses, and predict recovery, but also, it will help provide real-time medical monitoring, for instance, people in home-quarantine [4,5]. Hence, improving the precision and accuracy in blood pressure measurements can help significantly with early diagnosis and cardiovascular risk stratification [6,7,8,9], because inadequate performance in blood pressure measurement will increase current levels of fatal stroke and fatal myocardial infections [10], as well as impose an avoidable financial burden [11].

**Figure 1:**
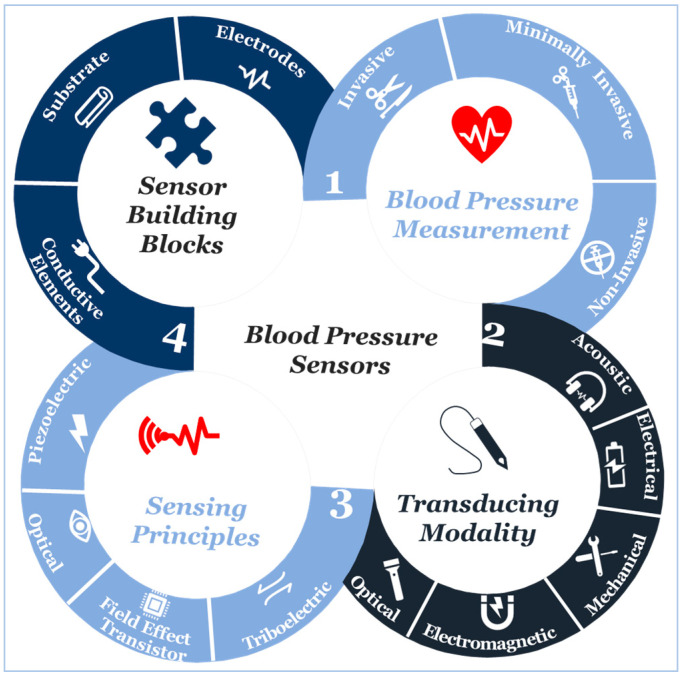
The transdisciplinary approach for the comprehensive review of the recent development in biomaterials used for an accurate yet continuous blood pressure measurement.

At the beginning of the twenty-first century, the use of sensors and mobile internet begins to provide a platform to continuously monitor all vital signs [12,13,14,15,16,17,18,19,20], including blood pressure. Not only does this help reduce the risk of cardiovascular complications, but also it supports making accurate and real-time healthcare data available for healthcare professionals at the office to assist select the best treatment strategies and consider the impact on patient outcomes [21,22,23,24,25,26]. Furthermore, this type of monitoring can save millions of lives around the globe annually [27,28,29]. Advancements in engineering and material science have been the main driver in the development of sensor technologies during the past decade [29,30,31]. Indeed, tactile sensors, and more precisely, skin-like soft electronics begin to transform healthcare [32,33,34]. In return, several studies highlight the crucial implications of this field and indicate that a timely review is necessary [35,36,37]. Since most studies focus on device functionality [38,39,40], there is a need to investigate device clinical performance and capabilities beyond proof-of-concept measurements outside of the laboratory [41], following standardized evaluation approaches [42]. By precisely studying the unique nature of medical needs and evaluating the functionality of sensing principles and materials, we will comprehensively identify materials’ properties and their associated performance in line with structure strategies needed for accurate and continuous blood pressure measurement. Also, we will identify challenges along with future research opportunities. We aim to create a crosslink between healthcare practice and material science following a transdisciplinary approach illustrated in (Figure 1) to emphasize the importance of design and fabrication elements that have been either overlooked or compromised.

## 2. Blood Pressure Measurement

**Figure 2:**
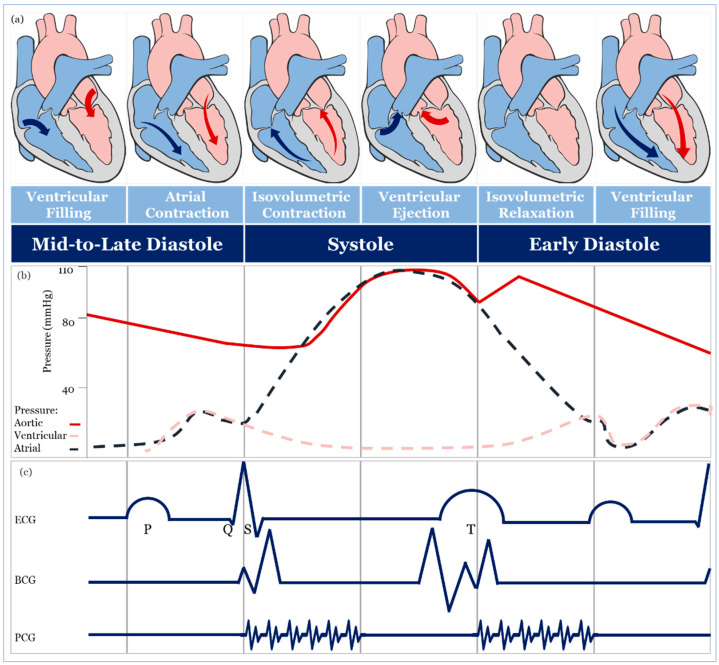
Schematic diagrams: (**a**) cardiac cycle, (**b**) arterial blood pressure versus ventricular and atrial blood pressure values, (**c**) morphological shapes of different signals associated with blood pressure.

The theoretical and practical framework behind accurate blood pressure measurement is complex and, sometimes, overlooked entirely [43], therefore, understanding the effect of different approaches for blood pressure measurement is essential for developing accurate sensing materials suitable for medical use. The volume of blood ejected by the heart into the arteries, the elastance or stiffness of the walls of the arteries, and the rate at which the blood flows out of the arteries altogether affect blood pressure measurement [44]. During the cardiac cycle (Figure 2a,b), systolic pressure occurs as blood is ejected out of the heart and into the arteries, and diastolic pressure is created when the heart rests between heartbeats [43,44,45].

**Figure 3:**
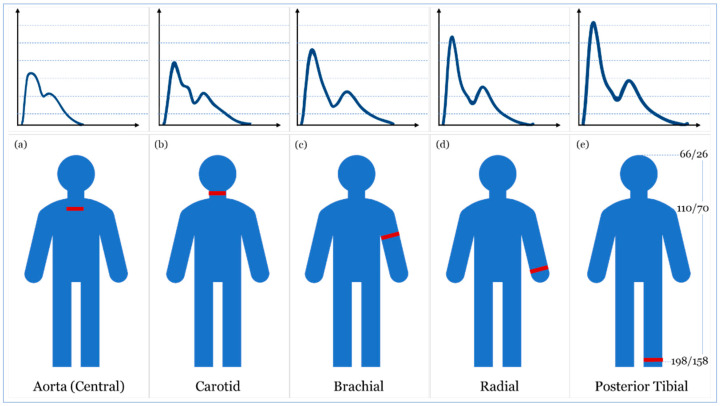
Schematic diagrams (**a**–**e**) illustrate the impact of artery stiffness and location on a blood pressure wave traveling through different arteries in an upright position. (**e**) shows blood pressure measurements in (mmHg) at three different locations (i.e., levels) in a person who is 182 cm tall: top of the head, heart, and foot.

In a healthy individual, systolic blood pressure and diastolic blood pressure are 110–115 mmHg and 70–75 mmHg, respectively [44,45,46]. A blood pressure measure out of this range may be associated with the incidence of several cardiovascular events (e.g., stroke, heart failure, and end-stage renal disease) [6,47]. Since arterial pressure varies continuously during the cardiac cycle, the morphological shape of different signals associated with blood pressure varies as well, as seen in Figure 2c. This shape is acquired as an electrocardiograph (ECG), ballistocardiograph (BCG), and phonocardiograph (PCG) signals [48,49,50]. Also, the morphological shapes and values of blood pressure wave vary when it travels from the highly elastic central arteries to the stiffer ones [51], and when it travels away from the heart either upward (i.e., towards the head) or downward (i.e., towards the foot) in an upright position (Figure 3) [43,47]. In the next sections, we will discuss blood pressure measurement techniques and carefully evaluating their transducing modalities and materials for accurate real-time monitoring.

### 2.1. Invasive and Minimally Invasive Blood Pressure Measurement and Materials

Invasive blood pressure is directly measured by an intravascular catheter unit, which comprises of three main components: an intra-arterial cannula, an infusion tube, and a transducer [52,53]. The intra-arterial cannula is a short and parallel-sided cannula made of different materials such as Teflon^®^ [54], PU [55], PVC [56], Vialon [57] or silicone rubber [58] to reduce thrombosis and bacterial infections [59]. The cannula is connected to an infusion tube, and the catheter-tip is the pressure sensing component in the transducer assembly. The transducer assembly conventionally utilizes MEMS technology to convert pressure waves into electrical signals [60,61] using silicon-based [62] and non-silicon based MEMS, such as Ti/Pt metallic wire coated with PI/SU [63], PEDOT: PSS with a Ag protective layer on a flexible PDMS substrate [64] and PVF_2_ [65]. Other non-catheter-based pressure sensors include a capacitive-based bioresorbable POMaC/PGS/Mg on a (PHB/PHV) substrate sensor [66]. The invasive approach is accurate and free of operator bias. Indeed, it is considered the gold standard for all other measures [67,68].

Minimally-invasive blood pressure measurement is based on nonvascular implantable miniaturized sensors that are compatible with body tissues, and these devices can provide real-time monitoring of the cardiac cycle [69], including intravascular [70], intraocular [71] and intracranial [72] using different MEMS-based implantable blood pressure sensors including Au-PI diaphragms [73] and Si nanomembranes [74]. The accuracy of a minimally invasive approach, in contrast to the invasive, is still controversial, and it may be due to the drift in sensitivity over a long time that affects long-term accuracy [75].

### 2.2. Non-Invasive Blood Pressure Measurement and Materials

#### 2.2.1. Full Occlusion

The full-occlusion technique includes auscultatory [76], oscillometry [77], and palpatory [78]. auscultatory and oscillometry are comparable to a gold standard [6,79], unlike palpatory, which is not used because obtaining a diastolic blood pressure measurement is difficult and may lead to considerable error [78]. The accuracy of the oscillometry method can be highly affected by muscle contraction, noise artifacts, artery stiffness, age, and physical health [76], hence, validation and recalibration are crucial [6,76]. Auscultatory and oscillometry methods are intermittent [80] and different cuff types [81,82,83,84,85,86] and fabrics [87,88,89,90,91] may lead to different blood pressure measurements [92,93]. 

#### 2.2.2. Semi Occlusion

Semi-occlusion technique includes applanation tonometry [94,95,96,97], originally applied for monitoring intraocular blood pressure in glaucoma patients [98] using a Goldmann Applanation Tonometer [99] and quite recently contact lens-based sensors [100,101,102], and extended to include blood pressure measurement of the radial artery based on anisotropic conductive film [103] or a silicon-based MEMS sensing chip [104]. The accuracy of applanation tonometry is controversial, as it is highly dependent on artery location and changes in contact force required to maintain artery in an applanated status over time [105,106].

The volume clamp method of Peńăz, also known as vascular unloading, is a continuous blood pressure measurement [107], in which volumetric change in blood flow in a finger during the cardiac cycle is kept unchanged using a high-speed servo pump connected to a finger cuff and checked by a finger mounted photoplethysmography (PPG) sensor [108,109,110,111,112,113]. Several clinical studies demonstrated the accuracy and reproducibility of volume clamp methods [108,114], however, their accuracy is still controversial because different finger-cuff types [110,111,112] and fabrics [113] may lead to different blood pressure readings. The broad assumptions behind the use of the PPG sensor [115] and the underestimation of the effect of the significant difference in hydrostatic blood pressure between the finger and the heart may lead to an increase in inaccuracy [116,117]. Besides, the volume clamp method requires recalibration at regular times leading to an overestimation of systolic pressure [118]. It is recognized that the finger-cuff can be uncomfortable for patients, especially patients suffering from edema or patients with impaired peripheral blood flow [119,120].

#### 2.2.3. No Occlusion

No-occlusion blood pressure measurement includes blood flow, pulse wave, and stroke volume methods. In the blood flow method, blood pressure is estimated utilizing the bifurcated or diseased artery geometry and the pulsatile blood flow equations [121]. The pulse wave method is a simplified form of pulsatile blood flow equations under certain assumptions is used [122]. In the stroke volume method, mean arterial blood pressure is estimated through measuring changes in the volume of blood pumped from the left ventricle (i.e., cardiac output) and the resistance that must be overcome to push blood and create flow in arteries (i.e., systemic vascular resistance) or through estimating cardiac output from O_2_ consumption levels [123]. The accuracy of the contact [124,125] and non-contact [126,127,128] sensing modalities of the blood flow method is controversial. Contact sensing has met the gold standard level of accuracy under certain conditions and failed to meet it under others, whereas non-contact sensing modalities show a significant reduction in diagnostic performance [127,128]. Likewise, a non-invasive form of FFR (i.e., FFR_CT_) has been described, with some studies showing that it is safe and feasible [129] and with others showing that current clinical trial data are insufficient to make a recommendation for its use in clinical practice [130,131,132].

Sensors based on stroke volume methods, including wearable ICG/ECG, are widely used [133]. The wearable ICG/ECG includes flexible dry electrodes made of a Ti-Au composite [134], a Ni-P plated polyester fabric [135], Ag flakes with MWCNT/PDMS composite [136], a woven fabric treated with PEDOT:PSS [137] and an Ecoflex-Ag MPs self-adhesive micropillar electrode inspired by gecko and grasshopper feet [138]. Furthermore, they can be fabricated of an EPDM rubber electrode containing various additives such as carbon, stainless steel fibers, and CNT [139]. 

Wearable ICG/ECG performance depends on the design and fabrication of high sensitivity electrodes and the continuous contact with skin, as well as their location when placed on the human body surface [139]. Also, their accuracy is mainly associated with the level of calculation complexity, which requires many mathematical assumptions, as well as measurement and physiological artifacts [108,140].

**Figure 4:**
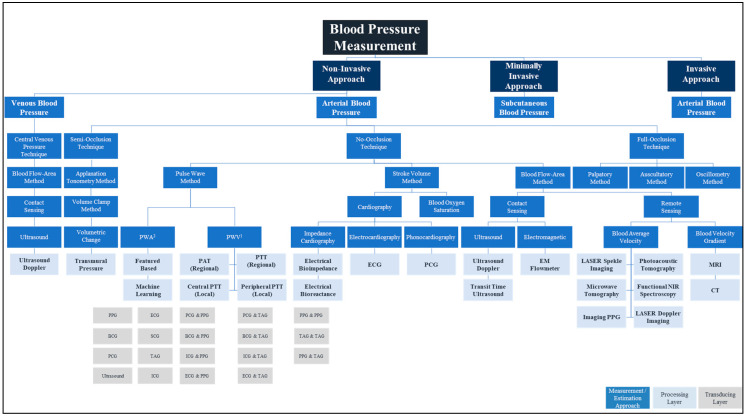
Blood pressure measurement landscape; approaches, methods, processing, and transducing layers. ^1^ PWA: At least a single pulsatility sensor or a single cardiovascular sensor is implemented; ^2^ PWV: At least two pulsatility sensors and/or additional cardiovascular sensor is implemented.

The pulse wave method is widely used in wearable and wireless applications due to its ability to integrate with a wide variety of transducers used in sensor application architectures. In addition to the effect of changes in measurement and physiological artifacts [141] and the pulse wave method does not collectively consider the impact of changes in physiological factors in blood viscosity, vascular wall elasticity, peripheral resistance of the arterial tree, and morphological characteristics in pressure pulse wave that vary regularly [142]. Figure 4 depicts the landscape of blood pressure measurement, approaches, methods, processing, and transducing modality layers, and Table 1 summarizes our analysis findings and highlights areas for further investigation. 

Non-invasive methods—with no-occlusion blood pressure measurements based on wearable devices—offer a promising future. Failing to choose the right materials for the fabrication of wearable devices can lead to either high noise in the received signal, which affects accuracy, or red and itchy rash in the skin caused by direct contact of the materials or even an allergic reaction to a body part causing highly frequent diseases that are clinically referred to by contact dermatitis [143]. Long direct contact of skin with medically unsuitable wearable device materials can foster an attractive and supportive environment for harmful microbiota, increasing the risk of infectious skin diseases, especially amongst patients with chronic diseases [144,145].

## 3. Transducing Modalities and Materials for Non-Invasive Blood Pressure Measurement 

The selection of the most suitable modality can efficiently enhance the accuracy of the non-invasive blood pressure measurement based on wearable devices. For instance, ballistocardiography (BCG) and seismocardiography have recently attracted attention [146] and several BCG and SCG transducing modality sensors utilizing different conductive materials have become available including PVDF [147] and PVDF/Au electrodes in a skin soft electronic tattoo [148], Au/PET and graphene/PMMA at thermal release tape (TRT) or tattoo paper [149], biaxial PP film based on EmFi [150] and a polysilicon surface-micromachined and monolith silicon based on MEMS [150,151].

Wearable and fixable design and fabrication of electrocardiography (ECG) and impedance cardiography (ICG) electrodes include the use of PEDOT: PSS and Ag plated electrodes [149], PVDF-based electrodes [152] and high-grade Ag coated fibers on a Textrode [153]. 

Wet Ag/AgCl electrodes are highly inconvenient for long-term applications because they lead to skin rashes and allergies with prolonged use [154] and when replacing wet electrodes with dry ones, dry electrodes have been reported to compromise user’s safety due to direct electrical contact between the skin and the electrode [155].Using non-contact capacitive coupled electrodes (CCEs) to overcome this safety issue may limit the use of ECG and ICG in continuous blood pressure monitoring [156]. Also, ECG and ICG provide spot measurement and are not suitable for long-term cardiac tracking for wearable devices worn on the wrist [157]. 

Skin patch sensors based on electromagnetic (EM) detection were built from a conductive trace of copper to measure intravascular stroke volume [124] and intracranial blood pressure [158]. Also, a magnetoelastic skin curvature sensor along with ECG electrodes was used to measure blood pressure in the carotid [159]. The use of the EM modality needs high levels of calculation complexity and relies on many mathematical assumptions that may affect the measurement accuracy. 

**Figure 5:**
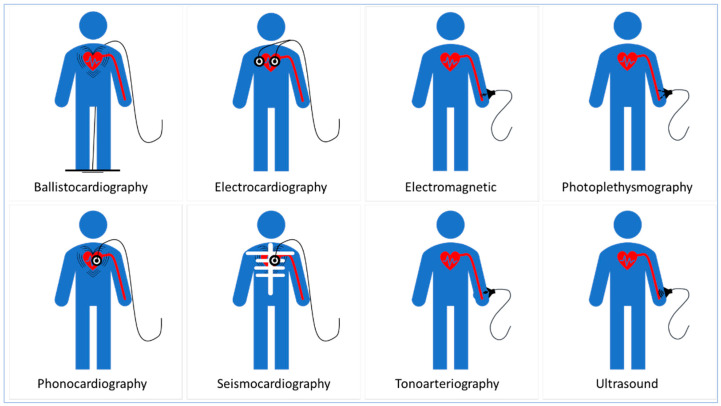
Transducing modalities used in blood pressure measurement.

Optical transduction based on PPG has disadvantages that can limit its usability for accurate, wearable, and continuous blood pressure measurement. The PPG working principle assumes that blood has a constant light absorptivity. In contrast, blood light absorptivity is highly affected by blood composition, particularly substances that have high absorptivity at NIR, such as hemoglobin [160,161]. Also, PPG requires direct contact with the skin that may cause discomfort to the user [162]. Furthermore, the use of the PPG-based transducing modality requires a stable contact force between the sensor and the measurement site [163]. The LED in PPG has a relatively limited light penetration depth (i.e., up to 8 mm). Hence, PPG use is limited to superficial arteries such as radial arteries and peripheral arterioles in fingers and ear lobes [164]. Furthermore, blood pressure waveforms cannot be extracted accurately from arteries adjacent to veins because any volumetric circulatory change in the artery will simply interfere with that in the vein [115]. Other transducing modalities including phonocardiography (PCG) using a PVDF-based sensor [165], tonoarteriography (TAG) using a flexible piezoresistive pressure sensor [166] and ultrasound using a piezo-pillar with filling epoxy on Cu and Cu/Sn electrode at Pi substrate [125] are promising. However, they rely on several mathematical assumptions, as well as physiological artifacts and measurements, including blood viscosity, vessel radius, and beam inclination that may affect their measurement accuracy. (Figure 5) illustrates transducing modalities used in blood pressure measurement. 

On the other hand, mechanical and acoustic transducing modalities have remarkably paved the way for more efficient signal-feature extraction that can reflect the insightful information on blood pressure dynamics [163,167,168,169,170]. In Table 2 we summarize transducing modalities along with some of the sensing principles associated with blood pressure measurement. 

## 4. Sensing Principles

In this section, we review the fundamental sensing principles and evaluate the feasibility of each to identify those with the required performance.

### 4.1. Piezoresistive

Creating a conductive network within an insulating matrix can be explained using the percolation theory [176]. The percolation theory explains the behavior of the composite matrix while transforming from an insulator to a conductor by increasing the content of a conducting filler or fiber gradually till the content reaches “the percolation threshold,” where the relationship between the measured electrical resistivity of the composite matrix and filler volume can be expressed as in Equation (1) [177]:

$$\rho =\rho_{0}{\left[v-v_{c}\right]}^{t},\;for\;v>v_{c}$$

where ρ resistivity of composites, $\rho_{0}$ resistivity of conductive filler, v filler volume, $v_{c}$ percolation threshold and *t* = critical exponent.

The piezoresistivity of the composite matrix dramatically increases several orders of magnitude as a result of the formation of continuous electron paths or conducting networks immediately after the filler volume fraction exceeds a percolation threshold. When the nanocomposite matrix reached an acceptable level of resistivity, the resistance-based electrical signal can be transduced into an applied pressure [178]. Therefore, the change in composite resistivity is mainly derived from two factors:

- The deformation in the composite geometry that may lead to changes in its length and cross-section area.
- The change in resistivity of the composite by changing the resistivity and/or volume of the conductive filler.

The piezoresistive sensing principle is widely used in wearable sensors, including the use of graphene and its derivatives [179,180], CNT and CB [181,182,183], metal NPs and NWs [184,185], conductive polymers [186,187] and MXenes [188,189].

### 4.2. Pizocapacitive

In a two-parallel-plate capacitor, capacitance is directly proportional to the relative static permittivity of the material between the two plates, the area of overlap of the two plates, and the electric conductivity of the material that comprise the two plates. Capacitance is inversely proportional to the separation distance between the two plates. A small amount of exerted force may cause the plates to deflect and capacitance to change [190]. Like the piezoresistive one, the piezocapacitive sensing principle is widely used in wearable sensors, including the use of graphene and its derivatives [191,192], CNT [193,194], metal NPs and NWs [195,196,197], conductive polymers [198,199] and MXenes [200].

### 4.3. Optical

Optical pressure sensors are devices through which light is often guided into a fiber or an optical waveguide. The applied pressure subsequently modifies the light intensity or wavelength through a Fiber Bragg Grating (FBG) configuration [201]. FBG wearable sensors have gained interest as they have electromagnetic immunity and high sensitivity that make them ideal for the use in an MRI environment with no threat to the patient and no influence over the quality of imaging [202,203,204] while using electrically non-conductive, bio-compatible and low optical permeability materials such as PDMS [205] and PA [206]. However, FBG-based sensing is constrained with the necessity of using the sophisticated signal analysis to detect breathing and cardiac activity [203], the little resistance to mechanical stress [206] and the limited range of Bragg wavelengths [204].

### 4.4. Field Effect Transistor

The field effect transistor (FET) pressure sensor utilizes transducing materials in the gate or channel region while controlling the flow of electrical current. The FET modalities include the graphene FET pressure sensor and its derivative [207,208], PVDF multimodal FET sensor [209] and pentacene/ P(VDF-TrFe) multifunctional OFET sensor. For most FET-based pressure sensors, the sensing mechanisms generally relies on the change in the capacitance of the dielectric layer, hence a small change in capacitance will generate a relatively high current signal output due to transistor signal amplification function [210,211].

### 4.5. Triboelectric

The triboelectric pressure sensor utilizes the effect of contact-induced electrification [212,213,214]. Generally, a material would become electrically charged after it comes into physical contact with another dissimilar material, and the strength of charges are different for different materials [212,213,214,215]. Triboelectric sensors include a textile-based sensor made of Ag-coated fabric [215], an eardrum-inspired active sensor made of an ITO-coated nylon thin film laminated onto a PET substrate [216], a membrane-based triboelectric sensor [217], a Downy-structured triboelectric nanogenerator (D-TENG)-based sensor [218], a 3D cellular sensor array [219], a flexible weaving constructed self-powered pressure sensor (WCSPS) [220] and a shape-memory PU (SMPU)-based sensor [221]. D-TENG-based and 3D cellular sensor array sensors showed encouraging results for blood pressure measurement [218,219], and flexible weaving constructed self-powered pressure sensors showed a discrepancy of about 0.87–3.65% when compared to a commercial cuff-based device [220]. However, triboelectric-based pressure sensors usually show a relatively low limit of detection [216,217] and its mechanism does not provide a stable and exact signal output that is sensitive to the magnitude, direction, and location of the applied stress in real-time [222]. Furthermore, the mechanism is profoundly affected by the amount of kinetic energy and momentum generated [223,224] that do not suit this type of application for accurate, yet continuous, blood pressure measurement and monitoring throughout all-day activities.

**Figure 6:**
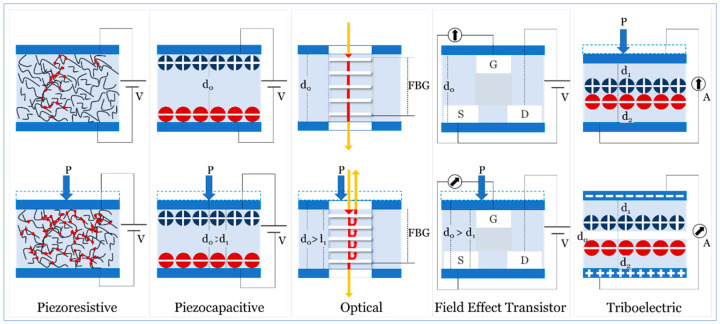
Fundamental sensing principles used in sensors.

Because of the findings we presented above, and because the triboelectric mechanism produces signals sensitive to humidity variations regardless of the use of a hydrophobic substrate structure [222] we will limit our coverage, in this review, to a few examples. The design and fabrication of hydrophobic surfaces, suitable for this mechanism, remain challenging for different combinations of materials to ensure stable operation under various service conditions. (Figure 6) illustrates the sensing principles we studied in this review.

### 4.6. Sensor Performance

**Figure 7:**
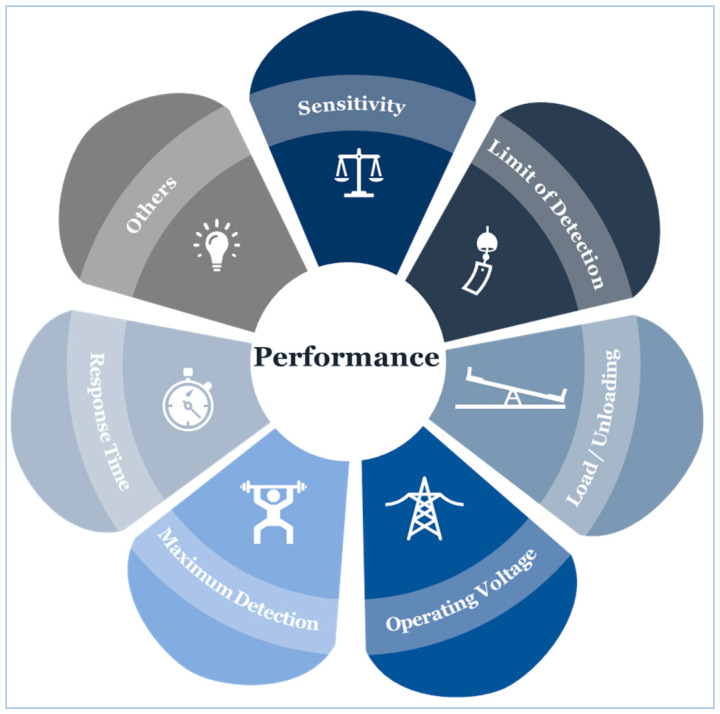
Essential performance elements necessary for accurate and continuous blood pressure measurement when compared to a gold standard.

We have carefully reviewed and considered several factors to retain the highest performance of different design and fabrication strategies. Setting a performance review criterion is crucial to select functional materials that can maintain essential qualities for interacting with biological systems in wearable devices [225]. For instance, mechanical pliability is important for devices that are in direct contact with some regions of the skin to minimize discomfort and respond to various strains associated with the body’s motion. Equally, suitability for medical use is important, too. The dimensions of our review criteria cover mainly the essential performance elements necessary for accurate and continuous blood pressure measurement when compared to a gold standard (Figure 7), including sensitivity, the limit of detection, and response time. Depending on information availability, additional dimensions were included, such as hysteresis, and simplicity in design. For example, hysteresis is a measure of the difference between the received signal and the applied force. High hysteresis is a significant disadvantage, and its effect, therefore, should be reduced.

## 5. Sensor Building Blocks

Active materials, electrode, and substrate are the essential components of a typical wearable sensor [226,227,228]. Besides, a dielectric material, which is an electrical insulator that can be polarized by an applied electric field, is used in some other sensors such as flexible FET-type sensors [211]. Flexibility, stretchability, and conductivity are amongst the most critical properties, hence, developing suitable functional materials with proper electrical and mechanical performance is of great importance to ensure maintaining high performance under various operating conditions. In this section, we review the development of active, substrate, and electrode materials, and study the effect of the use of different design and fabrication strategies of different materials on sensor performance.

### 5.1. Substrate

**Figure 8:**
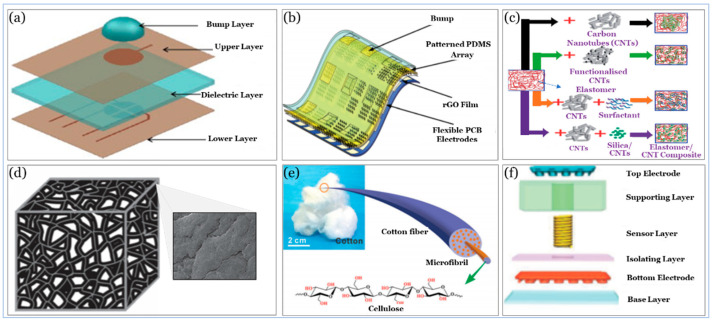
Substrate design and fabrication. (**a**) Bumpy design with PDMS substrate. Reproduced with permission [237] Copyright 2012, Elsevier. (**b**) Microstructure elastomer substrate. Reproduced with permission [238] Copyright 2018, Royal Society of Chemistry. (**c**) Elastomer composite. Reproduced with permission [239] Copyright 2014, Royal Society of Chemistry. (**d**) Porous composite based on a sponge. Reproduced with permission [240] Copyright 2016, Wiley. (**e**) Textile and thin films-cotton fiber substrate. Reproduced with permission [236] Copyright 2016, Royal Society of Chemistry. (**f**) Printed electronics substrate. Reproduced with permission [241] Copyright 2017, Wiley.

The substrate is considered the primary source for the stretchability of wearable sensors, and directly determines the level of comfort and long-term durability. The substrate is the outermost layer, which may directly contact body tissues, hence affects the safety and performance of the sensor. Materials used as substrates are primarily made of either polymers or natural materials, and their properties will depend on their chemical structures and processing approaches. PDMS is one of the most widely used material as a substrate for its high stretchability. It is non-toxic, non-flammable, and hydrophobic with acceptable processability. PET has good transparency, high creep resistance, and acceptable printability. Cellulose paper, on the other side, is biodegradable, biocompatible, recyclable, and inherently flexible, but its durability and stability are still amongst the most significant challenges for enhancing its properties. Table 3 illustrates some of the materials used as a substrate in wearable sensors along with their features and Figure 8 depicts different strategies for substrate design and fabrication.

PDMS materials can be processed quickly to form approximately any shape such as “bumpy” [237] that can be used to squeeze out an air gap to increase the capacitance. This design is simple and provides flexibility, but it cannot provide the desired limit of detection while maintaining capacitance. In addition to their stretchability and simplicity, several design and fabrication strategies for microstructure elastomer include a naturally molded substrate [242], and patterned substrate [238,243] where the combination of microstructure elastomers and active materials (i.e., sensing elements) are sandwiched by the patterned electrodes to create a resistive response that changes whenever a stimulus (e.g., pressure) is applied.

In respect to their low limit of detection and relatively high sensitivity, the sensors with naturally molded and patterned substrates are, sometimes, associated with low durability. The use of the elastomer composite substrate may overcome this drawback and others and provide a practical approach where active materials (e.g., CNTs) are incorporated into the elastomer matrix (e.g., PDMS) [239].

Active materials in the elastomer composite highly improve electrical conductivity and are useful for the reinforcement of the elastomer due to their low density and high aspect ratio. When such fillers are added to an elastomer, its mechanical properties, including tensile strength and hardness, increase, and this will depend on loading, dispersion, and alignment in the composite matrix; yet, this may affect the sensor sensitivity and reduce its limit of detection. As a result, the fabrication of a pressure sensor towards practical applications is becoming a challenging task.

There are different approaches to achieve high sensitivity, such as spray deposition [244] and multilayer spray coating [245], but they require sophisticated fabrication approaches. Other approaches, including porous composites with active material coatings [240], textile, and thin films [236] and printed electronics [241] have been recently utilized in tactile sensors, and they are used in different flexible electronic applications, such as artificial skin [246]. These approaches may help provide better mechanical properties in comparison with that of composite elastomers. However, the need to achieve high sensitivity without affecting other pressure performance elements, such as the limit of detection and durability, is still not adequately addressed. For example, Ag nanowire (NW) coated on cotton as a substrate [236] provides relatively high sensitivity (i.e., 3.4 kPa^−1^) and rapid response and relaxation time (i.e., <50 ms), but with relatively less flexibility (i.e., >5000 cycles). In contrast, CB at PU as a substrate [240] provides relatively higher sensitivity (i.e., 16.4 kPa^−1^), a faster response time (i.e., 20 ms), and higher durability (i.e., 50,000 cycles) but with a relatively low limit of detection (i.e., 91Pa) that does not fulfill blood pressure measurement requirements. In the next section, we will address this challenge in a more systematic approach.

### 5.2. Active Materials

The active materials represent a crucial component to respond to external stimuli. Generally, high performing pressure sensors need to pose high conductivity, exceptional chemical stability, and durability, as well as high flexibility as such, adding to excellent performance that includes fast response and high sensitivity.

#### 5.2.1. Carbon Compound

**Figure 9:**
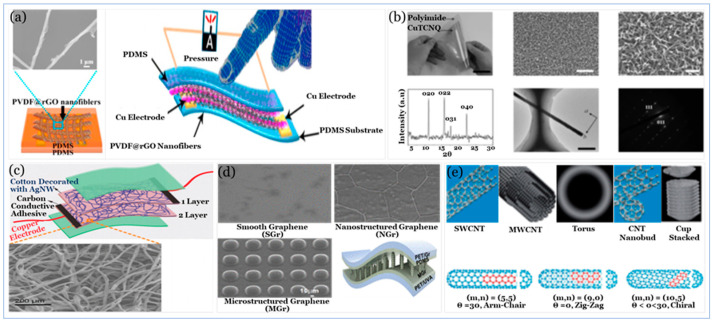
Conductive elements (**a**) Conducting Polymer. Reproduced with permission [251] Copyright 2016, Elsevier. (**b**) MOF. Reproduced with permission [249] Copyright 2015, Wiley. (**c**) Ag NW conductive element. Reproduced with permission [236] Copyright 2016, Royal Society of Chemistry. (**d**) Graphene-based Microstructure Elastomer. Reproduced with permission [247] Copyright 2019, American Chemical Society (**e**) CNTs. Reproduced with permission [239] Copyright 2014, Royal Society of Chemistry.

Nano conducting materials including carbon materials, such as CNT [239] and graphene [247], NWs and nanoparticles (NPs) [236,248], metal-organic-framework (MOF), Mxens [249,250] and conducting polymers [251] are among the most commonly used conductive components (i.e., active materials) that can be either embedded into or placed on the elastomeric polymer substrates. Figure 9 illustrates the different types of active materials used in sensors.

CB is a form of paracrystalline carbon with different sizes and shapes; it has a relatively high surface-area-to-volume ratio and electrical conductivity [252]. CNTs are widely used as active materials due to their high electrical and thermal conductivity [253], great mechanical flexibility, and excellent carrier mobility [254]. There are generally two types of CNTs: SWCNTs and MWCNTs. SWCNTs are cylindrical with a diameter on the order of nanometres that may vary due to strain and different modes of wrapping. An SWCNT results when one layer of carbon atoms in two-dimensional hexagonal lattices is wrapped into the form of a long cylinder (i.e., aspect ratio 300–1000) [255].

**Figure 10:**
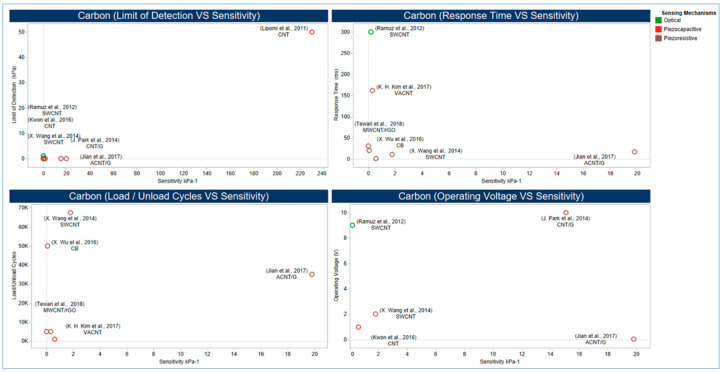
Effect of CNTs alignment on enhancing sensor performance.

**Figure 11:**
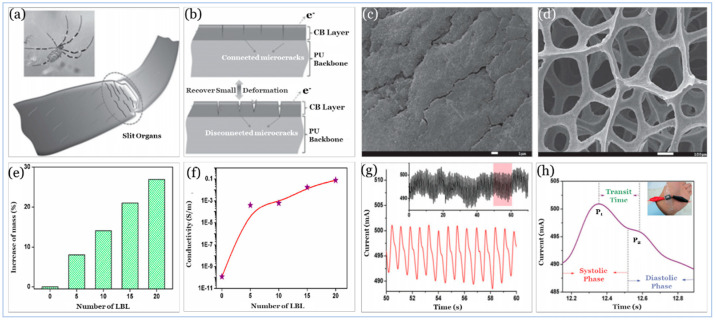
CB@PU Sponge. Reproduced with permission [240] Copyright 2016, Wiley. (**a**) Sketch of the crack-shaped slit organs near the leg joints of the spider. (**b**) SEM images of the microcrack junctions on a CB@PU sponge after compressing pre-treatment. (**c**) SEM images of an uncompressed CB@PU Sponge. (**d**) Comparison of mechanical properties of CB@PU Sponges with different Layer By Layer (LBL) deposition cycles of CB, a relative increase of CB mass on PU Sponges, (**e**) and an increase in conductivity of CB@PU Sponges. (**f**) with different LBL deposition cycles of CB. (**g**) Original signal of current curves for wrist pulse monitoring. (**h**) Zoomed waveform extracted from the original signal, showing some critical features that are essential for health monitoring.

**Figure 12:**
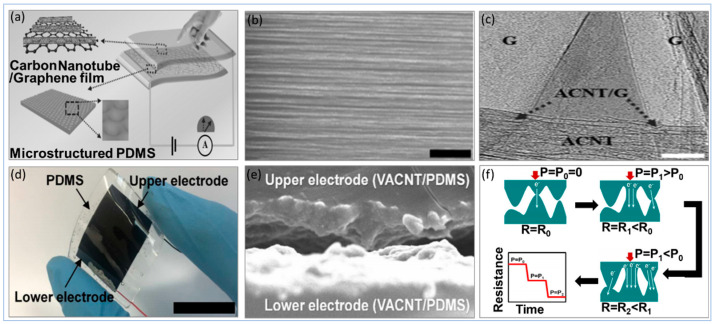
ACNT-Graphene@PDMS. Reproduced with permission [259] Copyright 2017, Wiley. (**a**) Schematic illustration showing the structure of the ACNT-Graphene pressure sensor. (**b**) SEM and optical images of continuous ACNT films drawn from VACNT arrays. (**c**) TEM image of ACNT-Graphene hybrid film. The areas marked by “G” indicate Graphene, and the area labelled by “ACNT” shows the ACNT film, and the regions mentioned by “ACNT/G” demonstrate the incorporation of ACNTs and Graphene. VACNT@PDMS. Reproduced with permission [260] Copyright 2017, American Chemical Society. (**d**) Digital image of the fabricated sensor. (**e**) Cross-sectional SEM image of the sensor. (**f**) Schematic illustration of the basic working principle of the sensor.

Utilizing different types of sensing principles, carbon materials as active materials have been used with PU as CB at PU sponge [240] and MWCNT with rGO at PU foam [256]. Also, it has been used with PDMS as CNT [257,258], aligned carbon nanotube (ACNT) with graphene [259] and vertically aligned carbon nanotube (VACNT) [260], as well as EcoFlex rubber as CNT [194,244]. In addition to the role of volume fraction and aspect ratio of the carbon materials in nanocomposite matrix in improving electrical and mechanical properties, the orientation of active materials within the nanocomposite matrix and substrate morphology (e.g., cell/pore size) can play a pivotal role in tremendously enhancing the matrix sensitivity, the limit of detection, and the durability. The effect of orientation and morphology is illustrated in Figure 10, Figure 11 and Figure 12.

Figure 10 provides a comprehensive dashboard view of the performance of carbon-based active materials where ACNT, VACNT, and CB based sensors showed the highest performance. Figure 11 depicts the performance of a CB based sensor inspired by spider crack mechanisms [240] that naturally increases sensitivity without negatively affecting mechanical properties. In this design, rigid and spherical CB NPs were selected as a conductive filler to form conductive layers on PU Sponge rather than fillers with a higher aspect ratio that could result in entangling or staking with each other, hence reducing flexibility and durability.

Figure 12 shows two different orientation techniques of CNTs on PDMS substrates to increase sensitivity and limit of detection without affecting flexibility. ACNTs architecture is essential for achieving enhanced fundamental characterization. For instance, it was shown that electron mobility of highly ordered CNTs films is 43 times higher than that of a random CNTs network [261] hence the CNTs volume faction in the nanocomposite matrix can be reduced to create a conducting percolation network when CNTs are aligned. Likewise, lowering CNTs volume fraction in nanocomposite matrix improves mechanical properties; storage modulus of ACNTs nanocomposite improved by approximately 40.0% as compared to the random CNTs [262] and tensile strength, as well as elongation of ACNTs fibrous composite enhanced by 150.0% and 62.5%, respectively, when compared to randomized CNTs fibrous composite with similar volume fraction [263].

#### 5.2.2. Graphene and Graphene Derivatives

Another carbon material used as an active material is graphene, which is a 2D allotrope of a single sp^2^ hybridized carbon atom graphite layer comprised of a monoatomic hexagonal lattice structure [264]. Graphene has unique properties, such as large surface area (i.e., up to 263 m^2^ g^−1^), high stiffness (i.e., up to 1.0 TPa), high thermal conductivity (i.e., up to 5000 W m^−1^ k^−1^), high electron mobility (i.e., up to 250,000 cm^2^ V^−1^ S^−1^), high electrical conductivity (i.e., 200,000 cm^2^ V^−1^ s^−1^), and tuneable bandgap [265]. GO is a chemically modified graphene with O_2_ functional groups such as epoxides, alcohols, and carboxylic acids. GO has received a great deal of attention because it readily exfoliates as single sheets in water [266]. rGO, on the other hand, is prepared from rGO by thermal, chemical, or electrical treatments. Hence there are always some defects resulting from unreduced O_2_ functional groups in or on the rGO surface, and subsequently, rGO cannot have the perfect graphene structure, which was described above [267].

Graphene has been used with PET substrates, such as a graphene electrode [190,247,268], a graphene FET [269] and a stencil mask [192]. Also, it has been used with PDMS, such as a graphene film [242] or as an rGO at PDMS [238] or as an rGO at PDMS and ITO at PET [243]. Also, it has been used as a graphene FET [270] and as a laser-induced graphene (LIG) [271] (Table 4).

Carbon compounds such as CNT and CB have been around for around thirty years, and they will keep attracting great interest within the scientific community owing to their superior mechanical, electric, and optoelectronic properties. Still, bio-inspired designs, fabrication strategies, other than the use of aligned primary conductive constituent in laminar composites, and alignment techniques need to be further investigated to meet the performance requirements of medical applications.

Graphene has been used with PI, such as a porous graphene (PG) sponge [276], interdigital electrode (IDE) [285] and with PU as rGO [281] or rGO with GO [273]. Furthermore, graphene has been used with materials such as silicon nitride in MEMS [274], PEN [278], PANI wrapped sponge [279], PMMA [277], DN hydrogels [280] and EcoFlex rubber [284]. Surprisingly, graphene has been also used with other unconventional materials such as tissue paper [275] polystyrene balls [283] and even a 3M VHB Tape [282].

**Figure 13:**
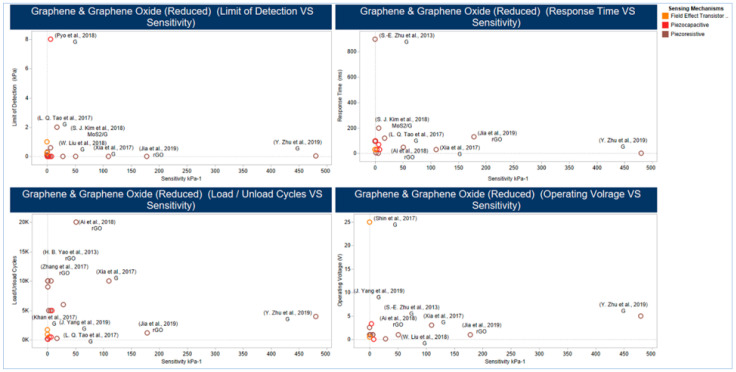
Effect of flexible 3D graphene structure with 2D electrical properties on sensor performance.

Getting a flexible 3D graphene structure with excellent 2D electrical properties was common across strategies used to design and fabricate the top-performing sensors with high sensitivity and flexibility, as illustrated in Figure 13. In the work of Xia and co-workers [242] a 3D graphene structure (i.e., fingerprint-like 3D graphene) was produced in a mechanism different from that of the seed-induced 2D graphene growth of the chemical vapor deposition (CVD) method. This mechanism considers the edge seed-induced growth in a hydrogen-rich environment. Furthermore, the use of hierarchal structure PDMS films (i.e., molded from natural leaves) improves the sensitivity as it provides support and abundant contact sites.

Zhu and co-workers [271] offer a scalable fabrication technology for producing 3D patterned graphene films (i.e., 50 µm) from PDMS with high conductivity and excellent mechanical properties. Direct laser scribing, in ambient air, provides high temperatures that can break Si-C, Si-O, and C-H bonds in PDMS. In return, Si and H atoms can combine with either the O_2_ in the air or the O_2_ atoms in PDMS to form SiO_2_ and gaseous water; the remaining carbon atoms are arranged into graphite structures.This process may be further elaborated through mechanisms of photothermal/photochemical process where the sp^3^ (i.e., carbon atoms are converted to sp^2^) carbon atoms by pulsed laser irradiation [298].

**Figure 14:**
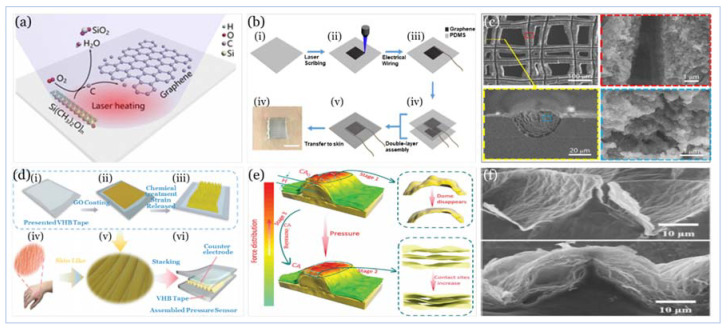
Graphene produced by Direct Laser Scribing of PDMS film. Reproduced with permission [271] Copyright 2019, American Chemical Society. (**a**) Mechanism of direct laser scribing of PDMS film. (**b**) (i–v) Schematic illustration of the fabrication process. (vi) Photograph of a sensor attached to the skin. (**c**) Morphology of the produced graphene with enlarged views at the position marked by red dashed box (top view), yellow dashed box (side view), and blue dashed box (enlarged view within side view). Skin-like pressure sensor with wrinkled reduced graphene oxide. Reproduced with permission [282] Copyright 2019, Royal Society of Chemistry. (**d**) Schematic illustration showing the fabrication procedure of the skin-like wrinkle film. (**e**) The dome-like microstructure is compressed at stage 1, the contact sites between the rGO sheets increase as the air gaps disappear at stage 2, and the wrinkle ridge begins to spread flatly at stage 3, respectively.(**f**) Cross-sectional SEM images of before (top) and after (bottom) reduction of the wrinkle GO showing the appearance of air gaps.

Jia and co-workers [282] present a novel technique, inspired by the microstructure of the human skin surface, where gradient rGO wrinkles are produced, through the reduction of coated GO on a pre-strained 3M VHB Tape. The high sensitivity is achieved through a three-stage contribution mechanism: the dome-like microstructure, caused by the expansion of the underlying rGO layer, increases the active contact area between the sensing layer and the counter electrode leading to an increase in the electron flow path. The gradual disappearance of air gaps, between the stack of GO sheets resulting from chemical treatment when undergoing applied pressure, will make the contact sites among the sheets to increase rapidly, and therefore the electron flow path will further increase significantly. The compression in the wrinkle ridge formed by the flexible matrix will increase the rate of change in the contact area that is positively correlated with sensitivity. Figure 14 illustrates the design and fabrication techniques of the work of Zhu and co-workers [271] and Jia and co-workers [282], as well as graphene and rGO properties.

Graphene has high electrical conductivity, it is more durable than diamond, yet it can be stretched by a quarter of its length, like soft rubber. With such an array of unique properties, Graphene allows for ground-breaking biomedical applications. Still, fabrication strategies, other than embedding aligned conductive material between layers of elastomeric material by different deposition or laser-induced techniques, and alignment techniques need to be further investigated to meet the performance requirements of medical applications.

#### 5.2.3. Conducting Polymers

**Figure 15:**
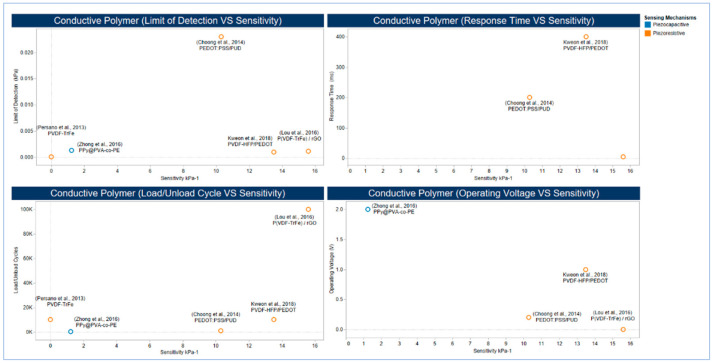
Effect of combined use of rGO and PVDF-TrFe nanofibers on sensor performance.

As more focus has been devoted recently to develop highly efficient piezoresistive pressure sensors, active materials other than carbon-based materials were studied. Ferroelectric polymers such as PVDF is considered as an essential category of frequently used active materials in terms of its flexibility and ease of processing [299]. PVDF is a semicrystalline homopolymer with the molecular formula [-CH2-CF2-]n, the crystallite polymorphs have five phases (α, β, Υ, δ, and ε) [300] The range of electrical dipole moments of the PVDF monomer is 5.0 × 10^−3^ °C.m for α-PVDF and 8.0 × 10^−3^ °C.m for β-PVDF [301,302] hence, conducting polymers are advantageous over other active materials due to their sensitivity and flexibility [303] PVDF has been used in many sensors [287] as PVDF-HFP and PEDOT [286] as well as, along with rGO [251] Furthermore, PEDOT: PSS and an aqueous PUD elastomer blend were used as a composite polymer with high conductivity [176] Another application is the utilization of PPy@PVA-co-PE Nanofibers at elastic POE Nanofibers [288] The composite film of rGO/PVDF showed an excellent performance that can measure the static and dynamic tactile and thermal signals, and this performance has been achieved through the use of rGO that boosted the sensitivity of Electrospun PVDF-TrFe Nanofibers, which in return, maintained flexibility in the nanocomposite. (Figure 15) illustrates the high performance of work [251].

Conducting polymers have tuneable conductivity and high processability through dispersion. They can play a pivotal role in tactile sensing by providing conductivity exceeding that of bulk metal electrodes [304] However, there is a need for investigation for new strategies to enhance mechanical properties and reusability further to meet the performance requirements of medical applications.

#### 5.2.4. Emerging Low Dimensional Materials

**Figure 16:**
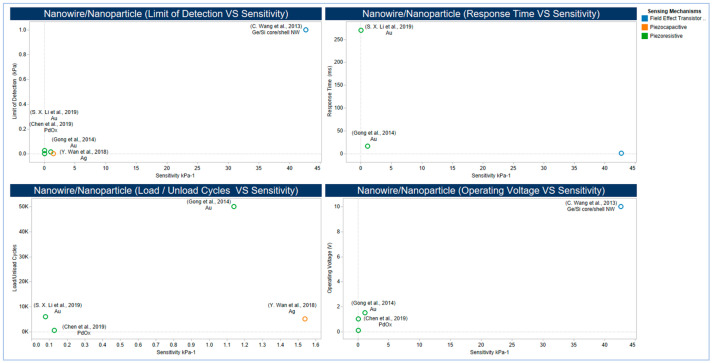
Effect of adoption ultrathin NWs and imprinting to control patterns and orientation of functional materials on sensor performance.

Unlike nanoparticles, which are on the order of nanometres with different shapes (e.g., semi-round, nano-cubes, spheres) [290], nanowires (NWs) are structures with a length typically on the order of microns and diameter on the order of tens of several hundred nanometres (i.e., high aspect ratio >1000) [305,306]. NWs have excellent mechanical properties when they are scaled below 100 nm. However, their resistivity change in response to applied stresses [307] and this makes them favourable for the use in a wide range of applications of piezoresistivity. Ultralong 1D metal NWs can be used to increase sensitivity without affecting flexibility because of their high aspect ratio lowers the percolation threshold of filler materials, resulting in high conductivity [308]. This novel and successful strategy have been used with ultrathin Au NWs (e.g., 2 nm) and high aspect ratio (e.g., >10,000) to provide outstanding performance for wearable sensors [185] with excellent biomedical capabilities [289]. Another strategy is the utilization of the imprinting technique to control the patterns and orientation of functional materials by template restriction [309] to assemble Au NPs in densely packed micro/NWs [290]. The effect of the adoption of these two strategies on sensor performance is illustrated in Figure 16. Owing to their high electrical conductivity, biocompatibility, and exceptional mechanical properties, metal nanoparticles have rarely been applied in flexible pressure sensors [290]. The use of metal nanoparticles may be an opportunity for simple fabrication strategies that strike a balance between meeting the performance of medical applications without an increase in fabrication complexity and cost.

#### 5.2.5. Metal-Organic Frameworks and MXenes

MOF materials are a class of chemical compounds consisting of metal ions or clusters coordinated to organic ligands to form one-dimensional structure, or two or three-dimensional structures [310] with the capability to form ultrahigh porous materials [311], with up to 90% free volume, and a huge internal surface area extending 6000 m^2^/g [312] Although most of the MOF materials are generally associated with a lack of electrical conductivity [313], a few have been reported as electronic conductors [313,314]. MOF materials have been thoroughly studied for major interests such as heterogeneous catalysis and biomedical applications [315,316].

**Figure 17:**
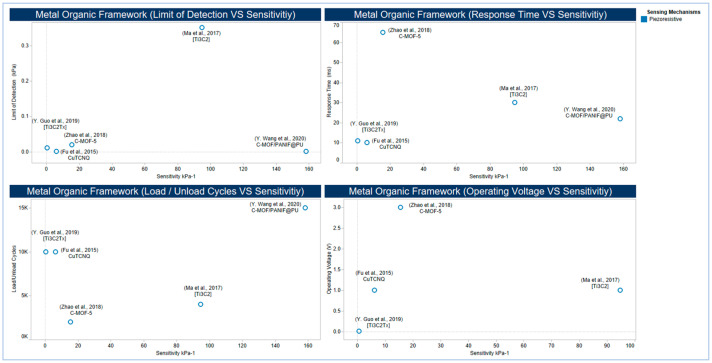
Performance of some MOF- and MXene-based sensors.

MXene materials are two-dimensional transition metal carbides, carbonitrides and nitrides with the general formula M_n+1_X_n_T_x_, where M represents an early transition metal, X is carbon and/or nitrogen, and Tx stands for surface terminations (e.g., OH, O, and F) [317]. In addition to the high flexibility of their interconnected networks in elastic matrices [318], Mxene materials show a unique combination of metallic conductivity and hydrophilicity resulting from the free electrons of transition metal carbide or nitride backbone and Mxene materials surface terminations, respectively [317] that allow them to be used in different applications such as energy storage [319] and pressure sensors [250]. Increasing sensitivity without affecting flexibility is a common objective in tactile sensor design and fabrication. As illustrated in Figure 17, several attempts have been performed utilizing different MOF materials such as the NW arrays of CuTCNQ at PI substrate [249] and C-MOF-PANIF at PU sponge [291], MOF-5 derived porous carbon and PDMS composite [292] as well as two different types of MXene nanosheet materials by sandwiching porous MXene-impregnated tissue paper between a biodegradable PLA and IDE-coated PLA thin sheets [200] and by fabricating a flexible sensor based on Ti_3_C_2_–MXene with IDEs [250].

The NW arrays of CuTCNQ at PI substrate [249] shows a low limit of detectability with higher flexibility due to the high sensitivity and flexibility provided by the combination of MOF nanocrystal arrays and PI substrate. Other design and fabrication strategies, such as the C-MOF-PANIF at PU sponge [291] show higher sensitivity but slower response time.

On the other side, the use of Ti_3_C_2_–MXene with IDEs [250] shows high sensitivity but relatively a higher limit of detection when compared with the work of Fu and co-workers, and Wang and co-workers. This high sensitivity might be due to the high strength of MXene materials as the effective Young’s modulus of a single layer of Ti_3_C_2_Tx is 0.33 ± 0.03 TPa, which is the highest among the mean values reported in nanoindentation experiments for other solution-processed 2D materials [320].

### 5.3. Electrodes

**Figure 18:**
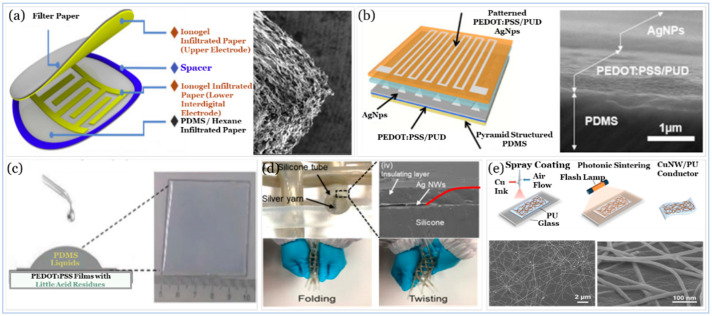
Sensor electrodes. (**a**) Nanocomposite electrode. Reproduced with permission [321] Copyright 2019, Elsevier. (**b**) Patterned electrode. Reproduced with permission [322] Copyright 2017, Elsevier. (**c**) Conducting polymer. Reproduced with permission [323] Copyright 2017, Wiley. (**d**) Conductive texture. Reproduced under an open access creative commons CC BY 4.0 license [324] Copyright 2017, MDPI. (**e**) Metallic electrode. Reproduced with permission [325] Copyright 2016, American Chemical Society.

Ideally, materials used for the design and fabrication of an electrode could maintain excellent conductivity under large stretchability without affecting its sensing stability, as this plays a pivotal role in maintaining the high accuracy of the signals generated in response to changes in the applied pressure. The adoption of different design and fabrication strategies, as illustrated in Figure 18, may lead to a trade-off between the electrode performance and the sensor performance.

Carbon materials such as CNTs and graphene composites are preferred for use as electrode over generally used metals, including Ag, Au, and Cu, for their high conductivity and flexibility [325]. Nanocomposite-based electrodes can provide mechanical flexibility and high conductivity for flexible sensing devices, reduce the material cost of Nobel metal deposition, and complex fabrication (e.g., lithography) and boost sustainability using environmentally friendly materials [321]. The conductive texture-based electrode provides an expandable tactile and strain sensor array [324]. Furthermore, their woven structured power-generating arrays can be integrated into a single multifactional device for piezoelectric energy harvesting and tactile sensing. The gauge factor of such sensors is much higher than that of the conventional metallic strain gauges. However, this gauge factor is less than that of a nanocomposite, for instance, made of MoS2/GF/Ecoflex [284]. Although this woven structure, as a strain sensor, shows acceptable linearity and sensitivity, it shows a hysteresis of a maximally 4.6% difference during stretching and releasing, whereas, a pressure sensor based percolative metal NPs arrays with Ag IDEs shows an insignificant level of hysteresis induced by a 1KPa applied pressure while maintaining a limit of detection and a sensitivity of 0.5 Pa and 0.13 kPa^−1^, respectively [248].

Printed electrodes can provide tremendous physical, mechanical, and electrical properties, such as low resistivity and high flexibility, while exhibiting a low limit of detection under low voltage without compromising on reliability and durability [322,326]. However, there are several challenges associated with this technology that include cost, synthesis, and choice of ink. For example, although both inkjet and screen printing technologies provide good flexibility at relatively low cost, both can offer a conductivity depending on the adhesion between the active materials and substrate that, in return, can be affected by several elements including the viscosity and temperature (i.e., curing) of the ink [327,328].

The use of conducting polymers such as PEDOT: PSS seeks to bridge the gaps in the performance associated with other electrode materials, including poor stretchability, low conductivity, and little stability [323]. Though, the acidic nature of PEDOT: PSS (pH is between 1 and 2) can cause corrosion to any metallic interface with the PEDOT: PSS-based electrodes that can degrade the overall performance of the device [329].

**Figure 19:**
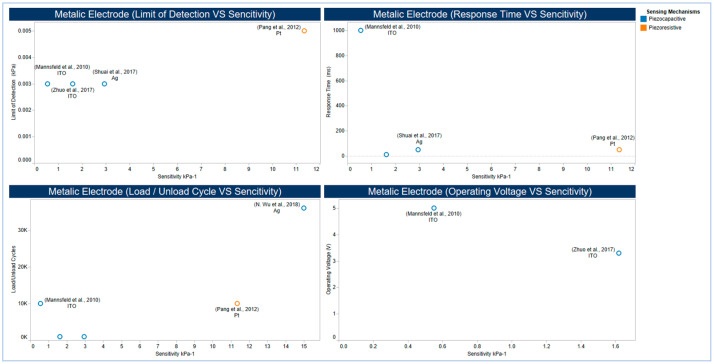
Performance of some sensors based on metallic electrodes.

Sensor-based metallic electrodes, as illustrated in (/unload cycles. These variations can be explained while taking into consideration the difference in the two dielectric and supporting materials applied, and the fabrication strategies followed, as well as the morphology and physical, mechanical, and electrical properties of the Ag used. Figure 19 shows different design and fabrication strategies, including the use of Pt, Ag, and some metal oxides. Using Pt coated polymeric nanofibers (i.e., nanohair) to fabricate a highly sensitive strain-gauge sensor, the work of Pang and co-workers [293] shows ultra-sensitivity with high flexibility when compared with that of the use of ITO electrode [294], which shows a better response time and a lower limit of detection. In the work of Shuai and co-workers [297] and the work of Wu and co-workers [295], tactile sensors are based on the use of Ag in both electrodes. However, they showed significant variations in flexibility in terms of the number of load/ unload cycles. These variations can be explained while taking into consideration the difference in the two dielectric and supporting materials applied, and the fabrication strategies followed, as well as the morphology and physical, mechanical, and electrical properties of the Ag used.

### 5.4. Operational Lifecycle

Wearable medical sensors are usually subject to scratches and mild damages that may limit their robustness and reduce their operational lifetime while affecting their mechanical and electrical capabilities. Equally, they need to maintain clean and safe during all-day activities while support a green environment. Being challenged to take new steps in the design of novel material concepts, researchers have become attracted to electronics that can mimic human skin, enable health monitoring such that they can self-clean [330,331] or self-heal after the wear and tear of every day, [332,333,334,335] or under environmental stresses [336,337,338] and unpredictable damages [339].

Several approaches and strategies have been articulated to enable built-in capabilities for autonomous cleaning [340,341,342], damage repair [343,344,345,346,347,348], diagnosis and reporting [349], as well as healing [350] and degradation [351]. We believe that understanding external and internal factors affecting device performance throughout its operational life cycle can help reduce material wastes and repair costs, as well as increase efficiency, safety, and reliability, which are highly desirable in clinical applications.

**Figure 20:**
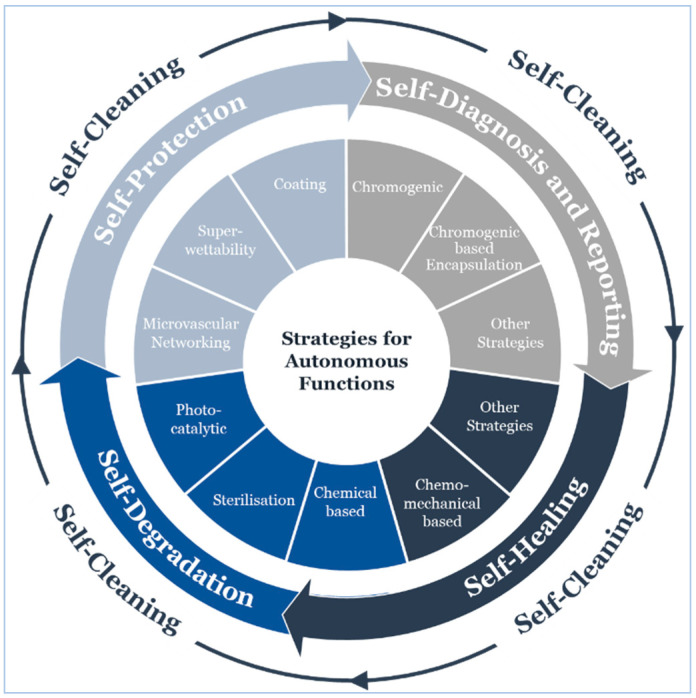
Strategies for autonomous functions throughout the operational lifecycle.

Capitalizing on the work of Patrick and coworkers [348], we developed an operational lifecycle of a wearable sensor that expands to cover four essential functions: self-protection, self-diagnosis and reporting, self-healing, and self-degradation while providing self-cleaning perpetually (Figure 20 illustrates strategies for autonomous functions throughout the operational lifecycle.

#### 5.4.1. Self-Cleaning

Generally, self-cleaning strategies aim to resist microbiota adhesion to a surface, such that they can aggravate biofilm formation [352]. A biofilm is created when planktonic organisms adhere to a surface excreting a slimy, glue-like substance that enables adhesion, and forming shear-resistant, matrix-embedded multicellular communities [353,354]. The use of antibiotics can largely aggravate biofilm formation, though, multi/pan-drug resistant infectious organisms have become a major public health issue [355,356]. Besides, antibiotics are limited to bacterial infections only [357]. Inspired by the Lotus Effect [358], material design that resists microbiota adhesion to a surface has become a powerful alternative strategy [359,360,361,362].

Adhesion of microbiota to solid surfaces is a complex process [363]; hence, several material properties need to be considered to ensure an effective material design strategy. The following is a summary of such features:

*Surface Energy*: Bacterial adhesion is affected by the physicochemical properties of the bacterial cell; hence, surface free energy is one of the most influencing factors of bacterial adhesion at the early stages of biofilm formation [364,365]. Materials with relatively low surface free energy reduce the tendency of bacterial adhesion to surfaces when compared to that of higher surface energies [320,366]. Indeed, this is almost always applicable to viruses since the wet and dry depositions of viruses are usually associated with either bacteria or particulate matter (PM) [367,368]. However, the earlier the first interfacial adhesion is initiated, the sooner the effect of other forces take place [369].

*Surface Chargeability*: The cell surface charge is created from the dissociation or protonation of functional groups that include carboxylate, phosphate and amine moieties [370,371]. With few exceptions, most bacterial and viral cell surfaces carry negative charges under certain physicochemical conditions [372,373]. Hence, positively charged surfaces attract bacterial cells, and electrostatic repulsion disrupts cell contact with negatively charged surfaces. However, electrostatic repulsion disruption can be compromised by extracellular structures that promote adhesion, including fimbriae, flagella, curli, and pili [374].

*Surface Roughness and Microtopography*: The substrate topography at the micro/nanoscale does influence bacterial detachment behavior, particularly, the number of cells attached to the surface, and their orientation relative to the surface details [375,376]. The irregularities on a surface promote bacterial adhesion and biofilm formation, while a smooth surface does not support bacterial adhesion and biofilm formation [377]. This bacterial adhesion can be explained through the large surface area produced by a rough surface in comparison with a smooth one.

*Surface Wettability*: The wettability of either bacterial cells or the surface in contact does affect bacterial adhesion. Bacteria with a hydrophobic cell surface prefer hydrophobic material surfaces, whereas those with a hydrophilic cell surface prefer hydrophilic surfaces [331,378]. Furthermore, bacteria adhesion can be mediated by the properties of the suspending medium, including surface tension, pH, ionic strength, and temperature [379]. Solid materials under several environmental conditions attract various organic and inorganic matters to adsorb to their surfaces before microbiota adhesion, forming a layer called the conditioning film [380,381,382]. Bacterial strains may influence the effect of conditioning films on bacterial adhesion, and this can significantly modify surfaces physicochemical and topographical properties, and subsequently lead to unpredictable deviations from the expected outcomes [380].

Developing mechanisms that consider the effect of surface properties on microbiota adhesion, many studies recommend different strategies to resist such an adhesion. However, more than one mechanism is probably involved at the same time, as these mechanisms have different ranges of action [380]. Hence, we summarize below some of these strategies based on experimental outcomes:

The selection of substrates with surface free energy in the range 20–27 mN/m [330,331], including the use of coating materials such as silicone elastomer, perfluoroalkoxyalkane (PFA) and polytetrafluoroethylene (PTFE) [330]. More information is available in the work of Van der Mei and co-workers [383]. Also, surface free energy difference can provide an accurate and straightforward thermodynamic measure for quantitatively predicting bacterial adhesion [384].

The selection of hydrophobic substrates with increasing surface stiffness, including substrate surfaces made of PDMS in the range 2.4–2.8 MPa [340,341,342]. On more rigid polymer-brush coatings, Gram-positive bacteria (i.e., *Staphylococcus epidermidis*) desorb more readily. In contrast, softer polymer-brush coatings deform upon adhesion that strongly increases the normally oriented adhesion force, which impedes desorption, decreases the elastic modulus (i.e., stiffness) drastically, and increases the loss factor (i.e., viscous portion) [385,386]. Shrink-induced superhydrophobic substrate surfaces, such as PE, PS, and PC, highly prevent *E. coli* bacteria growth [387]. However, the exposure of a substrate surface to a certain medium (e.g., PU to human urine) will render the surface more hydrophilic [388,389]. Also, the adhesion behaviors of Gram-positive bacteria on wettable surfaces are crucially mediated by peptidoglycan (PGN) through direct interaction with the surfaces, but with the highest adhesion to surface when using superhydrophobic bioinspired hierarchal surfaces (e.g., a rose petal-like structure) [390]. Contrariwise, the adhesion behavior of Gram-negative bacteria on wettable surfaces does not consistently agree with that of the Gram-positive bacteria, but generally, they show low adhesion behavior when superhydrophobic surfaces were used [390]. Another potential factor is the sufficiency of the amount of the extracellular polysaccharides (EPS) that the bacteria form to anchor to a surface adequately. It has been observed that Gram-negative bacteria strains (i.e., *Pseudomonas aeruginosa*) were unable to colonize at a superhydrophobic titanium surface fabricated by femtosecond laser ablation. In contrast, Gram-positive bacteria strains (i.e., *Staphylococcus aureus*) were able to colonize the same surface structure [360].

The selection of substrates with smooth surfaces that have a minimum height difference between the highest peak and the lowest valley (i.e., Z range), including substrate surfaces made of PDMS with a Z-range of 15.5 and polystyrene 96-microwell plates with a z-range of 34.8 [379]. Changing the roughness and microtopography using an engineered surface of a sharkskin microstructure patterned on a PDMS substrate reduced *S. aureus* bacteria adhesion by 93.0% [391] and similarly, using an engineered surface of a sharkskin microstructure imprinted with TIO_2_ NPs on a PET substrate reduced *E. coli* bacteria adhesion by 70.0–85.0% [392]. Other materials such as the superhydrophobic surface of a PS fibrous web hydrophobized by PFDTS vapor deposition with reduced solid area fraction displayed self-cleaning ability [393] Also, bacterial adhesion (i.e., *Pseudomonas aeruginosa*) to the surface of PS colloidal crystals (i.e., spheres of 1500 nanometers in diameter) was found to be less than that of PS colloidal crystals but with a smaller diameter (i.e., spheres of 450 nanometers) suggesting that the greater spacing between favorable sites on the 1500-nanometer colloidal crystal hindered the early-stage biofilm formation by separation of cell bodies [359].

The use of electrical current for electro-eradication/inactivation of bacteria including the use of pulsed electric field (PEF) as an inactivation approach due to its effect on the cell wall and coat architecture of *Bacillus pumilus* [394] and the use of a 5.0 mA direct current (DC) for more than 40 min in deionized water shows an over 99.999 % inactivation of *E. coli* and *Staphylococcus aureus* on a uniform conductive membrane of blended carboxyl-functionalized MWCNT with a polysulfone polymer [395].

#### 5.4.2. Self-Protection

Increasing device expected lifetime by implementing preventive measures protecting it against different causes of damage is the main objective for self-protection strategies. Coatings generally provide mechanical [396], chemical [397] and weathering [398,399] protection that increases resistance to wear, and this can be achieved through polymeric and smart coatings. Polymeric coatings (e.g., PDMS [400,401], PMMA [179,402] and PVDF [403,404,405]) can form organic thin films onto a substrate to significantly modify surface reactivity towards various elements including corrosion, adhesion, and wear [406]. Likewise, inorganic coatings (e.g., graphene [407], Ag [408] and Au [409]) can modify surface properties providing corrosion and damage protection. Organic coatings can leverage substrate corrosion and damage resistance. For example, the use of PMMA coating increased adhesion between graphene and PDMS substrate [410] and PVA coatings increased adhesion between GNS and PU yarn [411]. Also, coating, as a self-protection strategy, can extend device longevity by providing a self-degradation property. For instance, TiO_2_ coatings are widely applied due to their safe use [412] and photocatalytic efficiency utilized in degrading organic pollutants into nontoxic inorganic molecules [413], since their efficiency can be further enhanced in the presence of a piezoelectric field [414]. Besides, TiO_2_ photocatalytic efficiency can be further utilized in photoinduced self-decomposition [415]. Silk and silk coatings can be applied to protect against mechanical and chemical degradation threats due to their nontoxicity [416] and exceptional, unique properties [417,418,419]. Since not long ago smart coatings have been increasingly applied to tactile sensors [420] where smart materials are designed to provide protection and remain passive unless promoted to perform a stimuli-based function when activated through certain changes within their environment such as magnetic fields, pH, light or temperature [421,422].

While encapsulated-based and memory-shape assisted self-healing coatings are also self-protection strategies that we will discuss in more detail under self-healing [423,424], the microvascular network is a self-healing strategy, but it can also be applied to protect against thermal-induced stresses. The use of microvascular networks to support a thermal management capability is principally applied to preserve the stiffness-destroying effect of the high temperatures [425]. Tactile sensors are not usually subject to extreme temperatures. In contrast, their temperature is significantly limited to either the user’s skin in contact with the wearable sensor or the external surroundings [426], nevertheless, fluctuation in outdoor and indoor temperature, for instance, can lead to a significant change in the cardiac cycle, which affects blood pressure [427,428] and equally, can induce hysteresis, which affects sensor sensitivity [429]. Therefore, microvascular networks may play a pivotal role in providing thermal stability to reduce/eliminate undesirable thermal hysteresis.

Super-wettability is another effective strategy for self-protection. Super-wettability can provide self-cleaning [430,431], chemical-shielding [432,433] and anti-corrosion [434,435] effects, to name a few. For instance, partially embedded perfluorosilane-coated graphene into TPU by a dissolution and resolidification method can make the resulting composite withstand the abrasion by sandpaper for 20.0 m and a strain up to 400% without losing superhydrophobicity [436].

#### 5.4.3. Self-Diagnosis and Reporting

The principal objective for self-diagnosis and report strategy is to indicate that a device has experienced mechanical stress or damage [437]. Microcracks in polymeric materials are often difficult to detect, yet they can jeopardize the device performance leading to failure. Strategies that enhance detection of damage are necessary to improve functionality, ensure safety, and boost reliability [438]. Since their inception, chromogenic-based polymeric materials have been creating a fundamental interest in the development of materials that change their color in response to an external stimulus [439]. Chromogenic-based self-diagnosis and reporting strategies include mechanochromic [440], thermochromic [441] and electrochromic [442]. Depending on the intensity of mechanical stress that a polymer can be subject to, the affected molecules within the polymeric chain can induce a chemical transformation in the responsive material (i.e., mechanophores) accompanied by a change in optical properties. Mechanophores include NITEC [443] and SP [444,445] which changes color in bulk polymers from yellow to red [446], as it has preferential mechanochemical activation in the stress direction [447]. Aside from mechanochromic, we found that less attention has paid to other chromogenic self-diagnosis and report strategies, particularly, the thermo- and electrochromic. Mechanophore- and mechanochemical-based sensors and probe molecules in the mechanochemistry research area have progressed considerably over the past years [448,449,450,451,452,453]. In contrast, thermo- and electrochromic have advanced differently with less attention on thermochromic fluorescence [454], thermochromic sensors [455] and electrochromic sensors [456]. Self-diagnosis and self-reporting, as well as, self-damage sensing that correlates the electrical conductivity of the CNTs or graphene flakes composite with damage severity [457,458,459], or utilizes electrical resistance tomography mapping [460] can be further improved using the unique properties of thermo- and electrochromic polymeric materials. Finally, chromogenic based encapsulation can be applied by micro- and nanocapsules [461,462] or microvascular networks (i.e., hollow glass fibers full of reactive liquids) to monitor the crack propagation.

#### 5.4.4. Self-Healing

The main purpose of mechanisms for self-healing is to restore original mechanical and electrical capabilities without affecting functional performance [463]. Self-healing strategies can be grouped into different categories when considering strategies by which the healing mechanism is integrated into the targeted material. One categorization is based on the capability of the material to self-heal [464], in which the automatic (i.e., intrinsic) self-healing materials do not need an external healing agent, and non-automatic (i.e., extrinsic) materials do require human intervention or may require external energy or pressure to trigger the self-healing mechanism [465,466]. Another categorization is based on the damage length scale [348]. In this review, we will discuss self-healing strategies while considering self-healing mechanisms and their associated agents, if required, as well as enablers.

Chemical-based self-healing is a strategy, which utilizes the built-in capabilities in the targeted material to initiate a chemical reaction whenever physical damage occurs. This strategy takes place at the smallest scale of the targeted material, where autonomous self-healing mechanisms based on chemical reactions are triggered through several classes, including a covalent bond [467], supramolecular interaction [468], hydrogen-bonding [469], ionic-interactions [470] and π-π stacking [471]. Chemo-mechanical based self-healing is another strategy, which utilizes a rehealing agent within the host matrix (i.e., targeted material) to initiate a chemical reaction in response to mechanical damage [472]. Mainly, there are two chemo-mechanical self-healing classes; this includes encapsulation [423,473] and microvascular networking [345,474]. Other strategies utilize a re-healing agent to initiate a chemical reaction with external stimuli, such as; magnetic field [475,476], UV [477] and light and heat [478] as well as, memory shape-assisted self-healing induced by heat [479] and magnetic fields, and UV [480]. Table 5 summarizes a list of self-healing mechanisms along with some of their structure design properties.

Regaining a high conductivity after the damage is crucial for sensor functionality, hence, different strategies were developed to ensure full recovery utilizing several self-healing mechanisms. For instance, graphene derivatives, particularly GO, offer various modifiable oxygen-based groups (e.g., OH-groups), and its conductivity can be further improved by reducing it into rGO [490,491]. These properties can be considered before the design and fabrication of a self-healable graphene-based sensor. The OH-groups on GO can help form polymeric chains leading to a self-healable structure based on hydrogen-bonds [486,492], ionic interactions [493] and interfacial metal-ligand coordination [494]. Equally important is the GO content in the composite matrix since it affects thermal, electrical, and mechanical properties inversely. It was observed that thermal conductivity healing could be completed even at the lowest content, while electrical conductivity was low at low continents and became higher till the percolation limit. Then, mechanical healing gradually increased with content until it reached a maximal level of 70% [495]. CNTs are favorable in sensors due to their high electric conductivity and ease of functionalization [496,497]. Hence, it is perfect for self-healable sensing structures based on metal-ligand coordination [498], electrostatic interactions [499], hydrogen bond [500,501] In microcapsule-based self-healing mechanism [502], healing temperature [503] and CNT content [504] affect the thermal, electrical, and mechanical properties differently. When using G-CNT altogether, the G-CNT heterostructures may induce a synergistic effect that can improve its tensile strength and self-healing property [505]. In addition to GO and CNT, different conductive materials, such as conductive polymer-based sensing structures, NWs and NPs, and MXenes are favorable to self-healing based on hydrogen bonds, ionic coordination interactions and physical entanglements (e.g., CPH(TOCNF/PAA-PPy) [506]) and hydrogen-bond (e.g., Ag NWs [507] and MXene [508]).

The progress that has been achieved in self-healing is very promising, and though novel materials and innovative strategies in a more comprehensive approach are needed. For instance, wearable devices need to be healed and fully restored to their functional performance under all-day activities and standard working conditions (e.g., at skin temperature or during daylight) with minimal external interactions. Likewise, wearable devices need to be healed utilizing biocompatible and biodegradable materials that are safe, yet reliable, for both humans and the environment.

#### 5.4.5. Self-Degradation

The world produces as much as 50 million tons of electronic and electrical waste (e-waste) a year, and only 20% of this is formally recycled. For instance, there is 100 times more gold in a ton of e-waste than in a ton of gold ore [509]. In medical applications, the large motion-induced artefacts that occur from the loose coupling between wearable devices and a body part undermine device clinical relevance and accuracy [510,511,512] and increase the need for stable components of the wearable devices that safely interface with the skin without causing complications similar to contact dermatitis [143,513]. It is thus urgent to develop environmentally friendly materials, of low cost, and high reusability meeting the required performance of electrical devices as the diverse and hazardous nature of this waste makes recycling difficult and expensive [514]. Degradability is important since self-protected and self-healable materials will continue performing until they ultimately reach the end of their operational life. Hence controlled degradation is not only required when the device performance has become unrestorable to its original state, such that self-healing is no more feasible but also is required when the device is needed for only a certain time. Therefore, the design and fabrication of robust devices that will decompose only when exposed to a certain stimulus are highly desirable. While degradable materials can be broken down into smaller constituent pieces, biodegradable materials can be broken into low molecular weight products as biologically benign or physiological conditions [515,516]. The concept of biodegradability can be further expanded to cover biocompatibility, where biocompatible materials can fulfill their desired functions in contact with a living system without producing an adverse effect or deleterious changes [517]. Biodegradable and biocompatible materials, knowns as bioresorbable materials [518], have attracted much attention in the past decade due to the potential environmental problems caused by traditional nonbiodegradable polymers, and the wide variety of application scenarios include their use in a wide variety of sensors to collect essential physiological information, such as blood pressure, strain, and temperature to name a few [519,520].

Inorganic nanomaterials generally have low biodegradability that can be a challenge in biomedical applications while avoiding nanotoxicity as a result of inhalation or skin exposure [521]. For instance, indium tin oxide or tin-doped indium oxide (ITO) are widely used as excellent inorganic transparent electrical conductors. However, studies suggested that ITO nanoparticles (NPs) possess genotoxic potential on human lungs [522], while others suggested ITO possesses toxicity on cells and organs [523,524] and a material safety data sheet on ITO stated that ITO causes skin irritation and severe eye irritation [525]. Although a range of toxicological studies has been conducted assessing various physicochemical characteristics of nanomaterials, the results have been inconsistent and definitive rules cannot yet be established [521] and reviews on the potential for nanoparticles to penetrate the skin have been mixed [526]. For instance, silver nanoparticles (ranging from 9.8–48.8 nm) coated with PVP has been reported, in vitro, to penetrate through both intact and damaged full-thickness human skin [527]. Besides, shape dependency and active metallic facets of silver nanoparticles have been reported, both in vitro and in vivo, to exhibit diverse skin penetration. It has also been reported that triangular nanoparticles, which appear as 2 nm thick and equilateral triangle plates with an average side length of 50 nm exhibit the lowest skin penetration measured in terms of density and concertation of silver nanoparticles in blood, and rod-shaped nanoparticles, which appears with an average and diameter of 50 and 20 nm, respectively, exhibits the highest [528]. Unlike Ag NPs and ITO, MXenes are 2D inorganic compounds having low cytotoxicity and can be used in antibacterial applications [529].

Natural and synthetic polymers are perfectly suited for biodegradable and biocompatible electronics as they provide a wide variety of material properties and functions that can be tuned using chemical structural modifications allowing conformal contact with complete tissue surface at cost-effective fabrication strategies [530,531]. Table 6 and Table 7 summarise some properties of natural and synthetic polymers utilized in biodegradable and/or biocompatible sensors as substrates, dielectrics, active materials (i.e., conductors), or semiconductors.

Interestingly, natural fabrics such as silk can be tuned to increase its susceptibility to specific sterilization techniques utilized to control its degradation rate [417]. Moreover, silk and silk coatings can provide corrosion resistance while performing as a smart coating [612] and likewise properties of some polymeric materials can be modified to offer longer shelf-life for end-use and allow the controlled degradation in response to UV light, for instance, in some special applications [613].

## 6. Outlook

**Figure 21:**
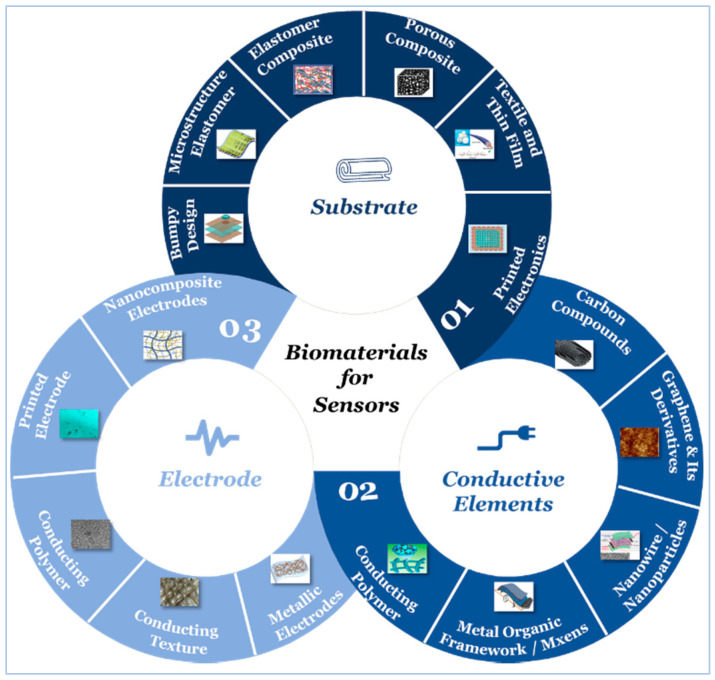
Use of biomaterials in sensor key components.

Biomaterials (Figure 21) have attracted considerable attention due to their exceptional performance and are, therefore, well-received as one of the future building blocks of digital healthcare. For blood pressure measurements and real-time monitoring, wearable sensors have not comprehensively reached a medically accepted level of functionality to replace intermittent cuff-based devices. This gap has been seemingly associated with a disconnect between the two knowledge areas, particularly, blood pressure measurement approaches and their related modalities at one side, and functional materials design and fabrication strategies and their relevant sensing mechanisms at the other one.

Invasive and minimally invasive blood pressure measurement does not support continuous measurement and real-time monitoring. Hence, ECG and PPG signals are mostly used to monitor health status without disturbing the users during daily activities. It would be beneficial to incorporate more comprehensive information collected from multi-modality signals such as mechanical (i.e., piezoresistive), thermal (i.e., thermoresistive), or even humidity to further improve the performance of current wearable sensors rather than considering a single sensing principle as illustrated in this review.

Apart from the rapid development demonstrated in this review, there is a bright future for tactile sensors utilizing smart materials (e.g., piezoelectric, self-healing, self-powering, self-cleaning), additive manufacturing, big data analytics (e.g., artificial intelligence) and cloud computing to fulfill healthcare demand for personalized medicine and remote monitoring. We found that sensitivity was the focus for most of the developed devices [247,248,249,251,257,258,259,260,274,275,276,277,278,279,280,281,282,283,284,285] and those with piezoresistive mechanisms generally showed high performance when compared with others [242,259,271,283,293]. Although piezocapacitive-based sensors still show excellent detectability and sensitivity, they are more susceptible to noise resulting from field interaction and fringing capacitance, as well as, other factors such as temperature [192,247,294,614]. FET-based sensors show high sensitivity with excellent response time due to their perfect functionalities of signal transduction and amplification, but their flexibility measured under continuous cycles of loading/unloading, is still a challenge [224,269,270,278] while the best performing device of this category shows a tiny bending radius lower than 0.02 cm with no significant variation in its electrical characteristics after more than 200 cycles [209] a piezoresistive-based sensor with a similar sensitivity can show consistent performance under continuous cycles of loading/unloading on the order of thousands. Also, FET based sensors require relatively higher operating voltage when compared to that of piezoresistive. This variation in performance should be considered as a motive, not only for further development of existing sensing principles but also for further investigation of novel mechanisms to go beyond meeting high sensitivity requirements to include linear performance without hysteresis.

The high sensitivity requirements have been achieved through the effective utilization of newly developed functional materials, such as PVDF/rGO [251] or ultrathin NWs [289], the optimization of device geometry, such as the design inspired by insects’ sensing capabilities [240] or plant leaves’ morphology [242] or the microstructure of human skin [282]. Other strategies include the reduction of active materials concentration in nanocomposite matrix through alignment [260], the utilization of novel additives manufacturing techniques such as LIG [271], or the imprinting technique to control the patterns and orientation of functional materials by template restriction [290]. The design and fabrication of wearable sensors with autonomous capabilities include wireless transmission [615,616] self-powered [220,617], self-healed [485] and self-degraded [519] will certainly enable continuous diagnosis of cardiovascular disease. However, these features and similar ones are no longer considered desirable, but they are becoming as crucial as other performance measures since they will allow automatic reparation of device malfunctions and disposal. Furthermore, the integrated sensing modality of tactile, temperature, and humidity [420,456,618,619] to extract additional features from other signals (e.g., respiratory) [620] with accuracy under the interface of strong body movement in real-time [621], will boost the performance of pressure measurements.

**Figure 22:**
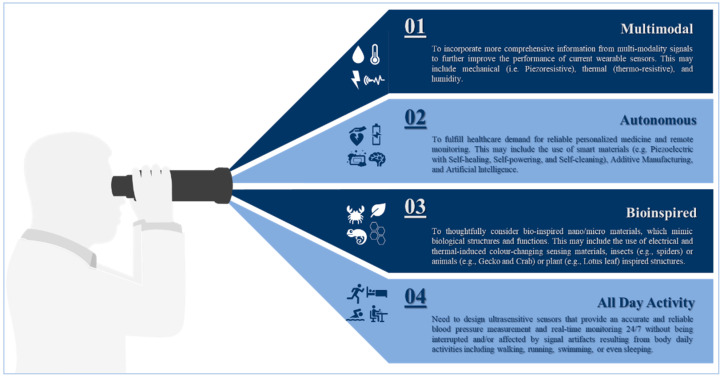
Summary of future development in the field of blood pressure measurement and real-time monitoring.

The performance of wearable sensors, capitalizing on the recent advancement in machine learning and cloud computation, can be further boosted by selecting optimal features that can contribute to dynamic blood pressure changes. In return, this will provide an accurate blood pressure measurement noninvasively and continuously, help enable early prevention and personalized treatment of hypertension, and reduce its burden on society. Finally, there is an essential need for a multidisciplinary approach encompasses of different knowledge areas, mainly, data, material, medical, and engineering sciences to ensure seamless integration between various sensor components and architecture, and address other challenges associated with sensor overall performance, as well as, evaluate the impact of each on device reliability and efficiency. Figure 22 summarises future development in blood pressure measurement and real-time monitoring.

## Figures and Tables

**Table 1:** Summary of current blood pressure measurement techniques and assessment of their potential for accurate yet continuous wearable blood pressure measurement devices.

SN	Approach	Technique	Method	Clinical Compliance		All-Day Activity ^1^		Remarks/Usability
				Periodicity ^2^	Accuracy ^3^	Wearable	Wireless	
1.0	Invasive	Single/Multisensory	Arterial Catheter	Continuous ^4^ [52,53]	Gold Standard [67,68]	N/A ^5^	N/A	Free of Operator Bias. Requires Experts/Clinics or Hospitals.
2.0	Minimally Invasive	Subcutaneous Blood Pressure	Subcutaneous implantable PPG	Beat by Beat ^6^ [69]	Controversial [75]	N/A	N/A	Free of Operator Bias. Requires Experts/Clinics or Hospitals.
3.0	Non-invasive	Full Occlusion	Auscultatory	Intermittent ^7^ [80]	Gold Standard [78]	Can Be ^8^	Can Be	• Operator Bias and White Coat Effect [6].
			Oscillometry	Intermittent [80]	Good [76]	Can Be	Can Be	• Affected by Artery Stiffness and Age [76].
			Palpatory	Intermittent [79]	Poor ^9^ [79]	Can Be	Can Be	• Operator Bias (i.e., Radial Pulse) [79]
		Semi Occlusion	Applanation Tonometry	Continuous [105,106]	Poor ^10^ [105,106]	Can Be	Can Be	Depends on Artery Location. [105,106]
			Volume Clamp	Continuous [107,108,109]	Controversial [110,111,112,113]	Yes	Can Be	• Complex Calibration may Lead to Overestimation of Blood Pressure [118].
			Blood Flow	Beat by Beat [107]	Controversial [107]	Yes	Yes	• Use of Contact Ultrasound Sensor [107]
		Non-Occlusion	Pulse Wave	Beat by Beat [122]	Controversial [141]	Yes ^11^	Yes	• Lack of changes in physiological factors [e.g., Blood Viscosity] [142].
			Stroke Volume	Beat by Beat ^12^ [123]	Controversial [139]	Yes	Yes	• Complex Calibration Due to Physiological Artefacts [108,140].
			Blood Flow ^13^	Beat by Beat [121]	Controversial [127,128]	Yes	Yes	• Needs to be further developed [127,128]

^1^ All Day Activity: Provides minimum capabilities for seamless 24/7 monitoring; ^2^ Periodicity: Provides information about blood pressure waveform; ^3^ Accuracy: Indicates the accuracy of blood pressure values when compared against an arterial catheter method; ^4^ Continuous: Provides a continuous blood pressure waveform; ^5^ N/A: Not Applicable; ^6^ Beat by Beat: Provides a single blood pressure value per heartbeat; ^7^ Intermittent: Provides an intermittent measurement of blood pressure waveform; ^8^ Can Be: it is not practical as it causes irritable contact; ^9^ Palpatory: Except for Systolic Pressure, it is difficult to measure diastolic pressure; ^10^ Applanation Tonometry: Except for eye tonometer, it is generally considered poor; ^11^ Yes: wearable, but this may affect accuracy due to motion/measurement artefacts; ^12^ Beat by Beat: this will depend on the method (e.g., O_2_ consumption); ^13^ Blood Flow: this includes arterial and venous blood pressure.

**Table 2:** List of transducing modalities with some of their associated sensing principles.

SN	Transducer Category	Transducing Modality	Sensing Principles			
			Potential	Capacitive	Piezoelectricity	Piezoresistivity
1.0	Acoustic	PCG	-	*	Piezoelectric Accelerometer [171]	-
		Ultrasound	-		LZT Sensor [172]	-
2.0	Electrical	ECG	Lead Electrode [173]	CCEs [156]	-	-
		ICG	Lead Electrode [174]	-	-	-
3.0	Electromagnetic	EM	-	PRF S-R Sensor [124]	-	-
4.0	Mechanical	BCG	-	Electromechanical Film Sensor [167]	-	-
		SCG	-	-	MEMS Accelerometer & Gyroscope [168]	
		TAG	-	Au/PEN [169]	-	Flexible Piezoresistance [170]
5.0	Optical	PPG	-	-	-	DPP-DTT: PCBM [175]

* Piezoelectric crystals appear electrically as capacitors (since they basically consist of two electrodes surrounding a dielectric material). However, at their resonant frequency they behave closer to a pure resistance.

**Table 3:** List of biomaterials used as a substrate in wearable sensors.

SN	Category	Substrate Material	Chemical Structure	Properties	Reference	Notes
1.0	Synthesis	PDMS	[C_2_H_6_OSi]n	High stretchability. Non-toxic, non-flammable, and hydrophobic. Acceptable Processability: ○ Soft lithography through plant leaf or piece of silk or micromachined silicon wafer. ○ Various fabricated patterns such as Groove, Pyramids, Hemispheres (e.g., Bumpy Design), Rods, and random-distributed channels with different sizes.	[229]	• Lack of biodegradability.
		Ecoflex^®^ Silicone Elastomer	-	High stretchability. Skin-safe silicone with lower modulus.	[230]	• Lack of biodegradability.
		PET	[C_10_H_8_O_4_]n	Acceptable transparency (i.e., >85.0%). High creep resistance and excellent printability.	[231]	• Relatively high modulus of elasticity (about 2~4 GPa).
		PI	C_35_H_28_N_2_O_7_	Acceptable flexibility. Acceptable creep resistance and tensile strength.	[232]	• Lack of biodegradability.
2.0	Natural	Cellulose Paper	[C_6_H_10_O_5_]n	Inherently flexible. Porous, inexpensive, and recyclable. Biodegradable and biocompatibility.	[176,233]	• Durability and stability are still amongst the biggest challenges for enhancing its properties.
		Smart Textile *	-	• Flexible, inexpensive, and biocompatible.	[234,235,236]	Low-level integration. Can be further developed by: Increasing conductivity/ sensitivity Making conductive materials absorbed into porous fibers and textiles. They include Cotton-Ag dipped and repeatedly dried in Ag NWs solutions for a high-performance pressure sensor.

* This includes silk and cotton where sensing chips are integrated into these textiles to realize a clothing-like sensing system.

**Table 4:** Summary of active materials, sensing principles used in wearable sensors along with their sensing properties.

SN	Active Material (Structure)	Sensing Principles	Limit of Detection (kPa) ^1^	Maximum Detection (kPa) ^2^	Sensitivity (kPa^−1^) ^3^	Reference	Notes
1.0	CNT/PDMS (Porous Structure)	Piezoresistive	0.25	100.0	0.588 ^4^	[257]	No blood pressure measurement application. Not all properties were reported (e.g., hysteresis, stability test). High limit of detection.
2.0	CNT@EcoFlex (Buckled Structure)	Piezocapacitive	50.0	1000	230	[244]	No blood pressure measurement application. Not all properties were reported (e.g., hysteresis, stability test). High limit of detection.
3.0	ACNT-Graphene /PDMS (CVD)	Piezoresistive	0.0006	0.3	19.8	[259]	No blood pressure measurement application. Response time is <16.7 ms. ^5^ Stability for more than 35,000 loading/unloading cycles. ^6^ Operating voltage is 0.03 V. ^7^ Low maximum detection.
4.0	CNT/PDMS (Patterned Microstructure)	Piezoresistive	0.0002	59.0	15.1	[258]	No blood pressure measurement application. Performance changed under cycle-test with constant pressure. Not all properties were reported. The operating voltage is 10.0 V. ^7^
5.0	VACNT/PDMS (T-CVD)	Piezoresistive	0.002	10.0	0.3 and up to 0.7	[260]	Blood pressure measurement application without calibration, need to assess accuracy. Response time is around 162 ms. ^5^ Acceptable reproducibility over 5000 cycles of pressure loading/ unloading. ^6^
6.0	SWCNTs/PDMS (Silk Molded Microstructure)	Piezoresistive	0.0006	1.2	1.8	[272]	No blood pressure measurement application but heart pulse. Response time is 10 ms. ^5^ Stability for around 67,500 loading/unloading cycles. ^6^ Operating voltage is 2.0 V. ^7^
7.0	CB@PU Sponge	Piezoresistive	0.091	16.4	0.068	[240]	No blood pressure measurement application but heart pulse. Stability for around 50,000 loading/unloading cycles. ^6^ Response time is 20 ms. ^5^
8.0	MWCNT-rGO@PU Foam	Piezoresistive	0.0035	2.7	0.022	[256]	No blood pressure measurement application but heart pulse. Stability for around 5000 loading/unloading cycles. ^6^ Response time is < 30 ms. ^5^
9.0	SWCNTs/PDMS	Optical	1.0	-	0.2	[201]	No blood pressure measurement application. Response time is 300 ms. ^5^ No stability test information was reported. Transparent optical application.
10.0	CNT/3D Microporous Elastomeric Dielectric Layer	Piezocapacitive	0.0001	130	0.601	[194]	No blood pressure measurement application but heart pulse. Stability for around 1000 loading/unloading cycles. ^6^ Operating voltage can be 1.0 V. ^7^
11.0	Graphene@PU	Piezoresistive	0.009	10.0	0.26	[273]	No blood pressure measurement application but heart pulse. Stability for around 10,000 loading/unloading cycles. ^6^ Not all properties were reported.
12.0	Graphene (MEMS)	Piezoresistive	0.1	-	3.4 × 10^−6^	[274]	No blood pressure measurement application. Response Time is 0.9 s. ^5^ Operating voltage is 2.5 V. ^7^ Not all properties were reported.
13.0	Graphene Paper	Piezocapacitive	2.0	20.0	17.2	[275]	No blood pressure measurement application, but heart pulse. Response time is120 ms. ^5^ Stability for around 300 loading/unloading cycles. ^6^ Fabricated by Dip Coating.
14.0	Graphene Electrode (T-CVD)	Piezocapacitive	4.4 × 10^−5^ (1 mg)	-	3.19	[247]	No blood pressure measurement application, but it sounds to be suitable for this application. The applied voltage is 3.3 V. ^7^ Stability for around 500 loading/unloading cycles. ^6^ Response time is 30 ms. ^5^
15.0	Graphene (Porous GS)	Piezoresistive	0.3	10.0	0.046	[276]	No blood pressure measurement application but heart pulse. Stability for around 200 loading/unloading cycles. ^6^
16.0	Graphene Electrode	Piezocapacitive	8.0	-	6.55	[268]	No blood pressure measurement application. Stability for around 500 loading/unloading cycles. ^6^ Response time is 70ms. ^5^ =
17.0	Suspended Graphene /Polymer (Heterostructure Membranes)	Piezocapacitive	80.0	-	123ZF	[277]	No blood pressure measurement application. Not all properties were reported.
18.0	Graphene Tribotronics	FET	1.0	-	0.02	[278]	No blood pressure measurement application. Stability for around 1700 loading/unloading cycles. ^6^ = Response time is 30 ms. ^5^ = Operating voltage can be 0.5 V. ^7^ = Suitable for electronic skin and touch screen applications.
19.0	rGO/PANI Wrapped Sponge	Piezoresistive	0.1	27.0	0.152	[279]	No blood pressure measurement application. Response time is around 96 ms. ^5^ = Stability for around 9000 loading/unloading cycles. ^6^ = High current output (i.e., ∼300 μA at 1.0 V. ^7^ =
20.0	PNIPAm/CMC/ rGO DN Hydrogel	Thermo-resisitive	-	800.0	-	[280]	No blood pressure measurement application. It is also sensitive to temperature (30 °C–45 °C).
21.0	rGO/PU Sponge	Piezoresistive	4.84 × 10^−5^ (1.1 mg)	-	0.21	[281]	No blood pressure measurement application. Stability for around 10,000 loading/unloading cycles. ^6^ = Response time is about 100 ms. ^5^ = Not all properties were reported.
22.0	rGO/PDMS Film (Pattered Micropyramid)	Piezoresistive	0.0015	1.4	5.5	[243]	No blood pressure measurement application. Response time is 0.2 ms. ^5^ = Stability for around 5000 loading/unloading cycles at 100 Pa. ^6^ = The applied voltage is 1.0 V. ^7^ =
23.0	rGO Films with Continuous Gradient Wrinkles	Piezoresistive	0.0042	3.0	178.0	[282]	No blood pressure measurement application. Stability for around 1200 loading/unloading cycles. ^6^ = Response time is 131 ms. ^5^ = Operating Voltage can be 1.0 V. ^7^ =
24.0	Large-Scale Polystyrene Ball@rGO Core Shell NPs	Piezoresistive	0.003	3.0	50.9	[283]	No blood pressure measurement application but heart pulse. Stability for around 20,000 loading/unloading cycles. ^6^ Response time is 50 ms. ^5^ Operating Voltage can be 1.0 V. ^7^
25.0	Graphene (Electrode Microconformal)	Piezocapacitive	4.4 × 10^−5^ (1 mg)	-	7.68	[190]	No blood pressure measurement application but heart pulse. Stability for around 5000 loading/unloading cycles. ^6^ The applied voltage is 1.0 mV. ^7^ Response time is 30ms. ^5^
26.0	Integrated Arrays of Air-Dielectric Graphene Transistors	FET	0.25	3000	2.05 × 10^−4^	[270]	No blood pressure measurement application. Response Time is 30 ms. ^5^ Stability for around 1000 loading/unloading cycles. ^6^ The gate voltage is 25.0 V. ^7^
27.0	Graphene Transistor Array (Direct-Contact Tribotronic Planar)	FET	0.16 mm^−1^	-	-	[269]	No blood pressure measurement application. Stability for around 1000 loading/unloading cycles. ^6^ Response time is 15 ms. ^5^ Gate voltage can be 2.0 V. ^7^
28.0	Graphene (Direct Laser Scribing PDMS)	Piezoresistive	0.028	-	480.0	[271]	No blood pressure measurement application. Stability for around 4000 loading/unloading cycles. ^6^ Response time is 0.002 ms. ^5^ Operating voltage can be 5.0 V. ^7^
29.0	3D Graphene Film (Fingerprint Like Patterned)	Piezoresistive	0.0002	75.0	110.0	[242]	No blood pressure measurement application but heart pulse. Stability for around 10,000 loading/unloading cycles. ^6^ Response time is 30 ms. ^5^ Operating voltage can be 3.0 V. ^7^
30.0	GO (Spray Coating through a Stencil Mask)	Piezocapacitive	0.24 × 10^−3^	-	0.8	[192]	No blood pressure measurement application. Response time is around 100 ms. ^5^ Maximum hysteresis is 5.0%@ 1.4 kPa. Stability for around 100 loading /unloading cycles. ^6^
31.0	MoS2/GPN /Ecoflex (T-CVD)	Piezoresistive	0.6	25.4	6.06	[284]	Suitable for blood pressure measurement application. Stability for around 4000 loading/unloading cycles. ^6^ Response Time is 200 ms. ^5^
32.0	PVA NWs/ Wrinkled Graphene Film	Piezoresistive	0.00224	-	28.34	[285]	No blood pressure measurement application. Stability for around 6000 loading/unloading cycles. ^6^ The applied voltage is 0.1V. ^7^ Not all properties were reported.
33.0	rGO Film/ PDMS Arrays	Piezoresistive	0.0013	225.0	1.71	[238]	No blood pressure measurement application. Stability for around 5000 loading/unloading cycles. ^6^ Response time is 6 ms. ^5^ Operating Voltage can be 1.0 V. ^7^
34.0	P(VDF-TrFe) /rGO	Piezoresistive	0.0012	-	15.6	[251]	No blood pressure measurement application. Stability for around 100,000 loading/unloading cycles. ^6^ Response time is 5 ms. ^5^ The applied voltage is 1.0 mV. ^7^
35.0	PEDOT:PSS /PUD (Pattered Micropyramid)	Piezoresistive	0.023	8.0	10.3	[176]	No blood pressure measurement application but heart pulse. Response time 0.2 s. ^5^ Stability 800 loading/unloading cycles. ^6^ Applied Voltage is 0.2 V. ^7^
36.0	PVDF-HFP/PEDOT (3D Electrospun Nanofibers)	Piezoresistive	0.001	30.0	13.5	[286]	No blood pressure measurement application but heart pulse. Stability for around 10,000 loading/unloading cycles. ^6^ Response time is around 0.4s. ^5^ The applied voltage is 1.0 V. ^7^
37.0	P(VDF-TrFe) (Electrospun Nanofiber)	Piezoresistive	0.0001	0.012	0.00041	[287]	No blood pressure measurement application. Stability for around 10,000 loading / unloading cycles. ^6^
38.0	[PPy@PVA-co-PE] and POE Nanofibers	Piezoresistive	0.0013	7.0	1.24	[288]	Suitable for blood pressure measurement application. Stability for around 250 loading/unloading cycles. ^6^ Operating voltage can be 2.0 V. ^7^
39.0	Au NWs/Tissue Paper (Dip-Coating)	Piezoresistive	0.013	-	1.14	[289]	No blood pressure measurement application but heart pulse. The operating voltage is 1.5 V ^7^ with low energy consumption that is <30 µW. Response time is <17 ms. ^5^ High Stability for around 50,000 loading/unloading cycles.
40.0	Au NP Densely Packed µNW based Pressure	Piezoresistive	0.025		0.0801	[290]	No blood pressure measurement application. Stability for around 6000 loading/unloading cycles. ^6^ Response time is <270 ms. ^5^ Not all properties were reported.
41.0	Ag IDEs and PdOx NP (Percolative Metal NP Arrays)	Piezoresistive	0.0005	1.0	0.13	[248]	No blood pressure measurement application. Stability for around 500 loading/unloading cycles. ^6^ Hysteresis induced by a 1.0 kPa applied pressure showed a shift of 0.012% from the initial value.
42.0	AG NWs (Ag NW Flower)	Piezocapacitive	0.0006	115.0	1.54	[195]	No blood pressure measurement application. Stability for around 5000 loading/unloading cycles. ^6^ Not all properties were reported.
43.0	Ge/Si Core/shell NW PSR (OLED)	FET	1.0	-	42.7	[210]	No blood pressure measurement application. Response time is 1 ms.5 Operating voltage is 10 V. ^7^ Not all properties were reported.
44.0	MOF (CuTCNQ)	Piezoresistive	0.00073	3.0	6.25	[249]	Suitable for blood pressure measurement application. Stability for around 10,000 loading/unloading cycles. ^6^ Response time is 10 ms. ^5^ Operating voltage can be 1.0 V. ^7^ MOF is relatively expensive.
45.0	C-MOF/PANIF @PU Sponge	Piezoresistive	0.001	60.0	158.26	[291]	No blood pressure measurement application, but heart pulse. Stability for around 15,000 loading/unloading cycles. ^6^ Response time is < 22 ms. ^5^
46.0	C-MOF-5 Derived Porous Carbon	Piezoresistive	0.02	1.0	15.63	[292]	No blood pressure measurement application. Stability for around 2000 loading/unloading cycles. ^6^ Response time is < 65 ms. ^5^ Operating voltage is 3.0 V. ^7^
47.0	MXenes Nanosheets (Ti3C2Tx)	Piezoresistive	0.0102	30.0	0.55	[200]	No blood pressure measurement application. Stability for around 10,000 loading/unloading cycles.6 Response time is less than 11 ms.5 Operating voltage is 0.01 V.7
48.0	MXenes Nanosheets (Ti3C2)	Piezoresistive	-	0.351	7.5	[250]	No blood pressure measurement application. Stability for around 4000 loading/unloading cycles. ^6^ Response time is < 30 ms. ^5^ Operating voltage is 1.0 V. ^7^
49.0	Pt-coated Polymeric Nanofibers (Nanohair)	Piezoresistive	0.005	-	11.35	[293]	No blood pressure measurement application. Response time is 50 ms. ^5^ Stability for around 10,000 loading/unloading cycles. ^6^
50.0	ITO (3D Printed Mold)	Piezocapacitive	0.003	4.0	1.62	[294]	No blood pressure measurement application but heart pulse. Stability for around 1000 loading/unloading cycles. ^6^ Operating voltage can be 3.0 V. ^7^ Response time is < 10 ms. ^5^
51.0	Ag Flexible Piezoelectret-Based Pressure Sensor	Piezoelectric	-	2.5	15.0	[295]	No blood pressure measurement application. Stability for around 36,000 loading/unloading cycles with constant pressure. ^6^ Perfluoro (alkoxy alkane) electret. Not all properties were reported.
52.0	ITO/PDMS (Pattered micro-pyramid)	Piezocapacitive	0.003	20.0	0.55	[296]	No blood pressure measurement application. Response time is 1.0 s. ^5^ Stability for around 10,000 loading/unloading cycles. ^6^ The gate voltage is higher than 5.0 V. ^7^
53.0	Ag NWs (Embedded PDMS Electrode with Microarray Structure)	Piezocapacitive	0.003	5.0	2.94	[297]	No blood pressure measurement application. Stability for around 1000 loading/unloading cycles. ^6^ Response time is less than 50 ms. ^5^ Not all properties were reported.

^1^ The smallest change that can be detected by a sensor. ^2^ The largest change that can be detected by a sensor. ^3^ The rate of change in output signal when responding to a change in stimulus. ^4^ Calculated Sensitivity. ^5^ The time interval within which a sensor can detect the smallest change in stimulus. ^6^ The rate of change in sensitivity and/or other sensor properties over continuous cycles of loading/unloading. ^7^ The sensor operating voltage should be as small as fractions of an mV to reduce power consumption, improve the battery life of the wearable system and/or support the integration with other energy harvesting applications.

**Table 5:** List of self-healing mechanisms, along with some of their structure design properties.

SN	Structure Design	Sensor Type	Self-Healing Mechanism	Self-Healing Material/Agent	Self-Healing Time (h)	Note
1.0	(f-BN NS)/PEDOT: PSS/PNIPam Hydrogel [469]	Pressure	Chemical bond interaction-based (Hydrogen-bond)	-	6.0	The self-healing starts at room temperature.
2.0	Ag NWs/rGO@m-PCL Microspheres onto PDMS [481]	Strain	Solid Microsphere	Ag NWs/rGO@m-PCL Microspheres	0.05 (3.0 min)	Heated at 80 °C for 1 min, then, self-healed for 3 min with a cut of 10 µm size. Acceptable conductive stability (25% drop in resistance) and sensitivity (0.16 rad^−1^ in bending downward direction) under cyclic bending.
3.0	MWCNT-PEDOT-PAM-PVA [482]	Pressure	Chemical bond interaction-based (Hydrogen-bond)	-	~1 s	The self-healing property of the hydrogel was evaluated by restoring conductivity (i.e., ~96.0%) instantly.
4.0	Au NP/Sh-crl-PU [483]	Gas analytes, pressure, strain, and temperature	Chemical bond interaction-based (Hydrogen-bond)	-	48.0	The self-healing time of a cutting groove of ~ 150 µm was 30 min at room temperature. For compete cut into two pieces, it requires 48 h. Self-healing can occur between −20 °C–50°C After self-healing, composite recovered ~ 90% of tensile strength and ~97% of elongation in comparison with pristine Sh-Crl-PU.
5.0	(Ti_3_C_2_Tx)/PV) hydrogel [484]	Strain	Chemical bond interaction-based (Hydrogen-bond)	-	~0.15 s	Instantaneous self-healing is ~0.15 s at room temperature. This sensor maintains its original performance after a self-healing.
6.0	PAA slightly crosslinked with PEG Ionic Conductive Ink [470]	Strain	Chemical bond interaction-based (Ionic Interaction)	-	0.5	The self-healing time was 30 min at room temperature when the hydrogel is cut into two pieces and then reattached to each other even if directions of the two pieces were changed.
7.0	Ternary Composite DMSO-mixed PEDOT: PSS with Triton X-100 Wearable thermoelectric generators [485]	Strain	Chemical bond interaction-based (Hydrogen-bond)	Triton X-100 >(C_14_H_22_O(C_2_H_4_O*_n_*)	1.0 s	The self-healing behavior was observed with the film thickness ≥20 µm and cutting width ≤100 µm.
8.0	PETMP-TTT Thiol-Ene Coatings [424]	-	Other Strategies (Shape memory assisted with heat)	-	0.083 (5.0 min)	Scratches produced on the PETMP-TTT polymer coatings with different constant loadings (1.2 N, 1.5 N, and 2.7 N) were completely self-healed after heating to 70 °C for 5 min. The crosslinked PETMP-TTT polymer network was also capable of initiating scratch recovery at ambient temperature conditions.
9.0	Conductive Polyimine Film (Dynamic Covalent Thermoset Polyimine with Ag NPs) [486]	Flow, humidity, tactile, and temperature	Other Strategies (Chemical Reaction assisted with heat and pressure)	Re-healing agent (TAA-DETA-TREN with EToH and Ag NPs)	4.0	The self-healing starts instantly. Heat-press (4 h at 80 °C and 8.5 kPa) was adopted to make the rehealed area more uniform and robust. This sensor is recyclable, too.
10.0	Stacked textile reinforcement with dual-channel [487]	-	3D Micro-vascular Networks	A mixture of DGEBA and Aliphatic amido-TETA	48.0	Two patterns of microvascular networks were applied (i.e., Parallel, and Herringbone). Herringbone pattern enhances mixing through increased interfacial and overlapping fluid boundary layers. Self-healing starts at 30 °C. Evaluation of continuous self-healing cycle.
11.0	rGO based Composite [488]	Pressure and flexion	Encapsulation	PBS confined in rGO networks with microscopic porosity	24.0	The self-healing and full-recovery start at ambient temperature for both mechanical and electrical properties.
12.0	Self-healing magnet-polymer composite [489]	Strain	Other Strategies (Shape memory assisted with a magnet)		0.167 10.0 min	The self-healing starts at ambient temperature when placing the torn edges of a test sample together between two glass slides while applying a magnetic field with an operating frequency of 475 kHz. Healing time is 10 min. Mn-Zn ferrite magnetic filler can trigger actuation, self-healing, and multiple cycle damage sensing.

**Table 6:** List of biocompatible and biodegradable materials used in wearable sensors as substrates and/or insulators.

SN	Material	Material Category	Chemical Structure	Young Modulus (MPa)/ Elongation (%)	Bio-compatible	Bio-degradable	Note
1.0	Cellulose Paper	Organic	-	17.6 ± 0.7/14.0 ± 0.4 [532]	Yes [533]	Yes [534]	Mechanical property values are based on cellulose paper made of steam-exploded bamboo microfibers. Mechanical property values are Tensile Strength and Elongation at the break. It can be used as an insulator [535] or substrate, [536] or all paper-based [537]
2.0	Ecoflex Silicone Elastomer 00-30	Organic	-	0.1/835 [538]	Yes [230,539]	-	Elongation value is at ƐMax. It can be used as an insulator [540] or substrate [541]
3.0	PCL	Organic	[C_6_H_10_O_2_]n	325±115/650± 350 [537]	Yes [542,543]	Yes [544]	Elongation value is at the break. It can be used as an insulator [545]
4.0	PDMS	Organic	[C_2_H_6_OSi]n	Hyperplastic [546]	Yes [547,548]	-	Usually used as a substrate [549,550] but can be used as an insulator [551].
5.0	PES	Organic	[C_12_H_8_O_3_S]n	3.76/47.66 [552]	Yes [553]	-	Elongation value is at the break. Bio-PES can be synthesized [554] It can be used as an insulator [555]
6.0	PET	Organic	[C_10_H_8_O_4_]n	19.59±0.22/1.87 ±0.03 [556]	Yes [557]	-	Mechanical properties are for Neat PET. Elongation value is at the break. They are usually used as a substrate. It can be used as an insulator [558,559] or substrate [560,561].
7.0	PI	Organic	C_35_H_28_N_2_O_7_	2010/27.5 [562]	Yes [563]	-	Mechanical properties are for Neat PI. Elongation value is at the break. They are usually used as a substrate. Bio-PI can be synthesized [564] It can be used as an insulator or substrate [565]
8.0	PLGA	Organic	C_5_H_8_O_5_	-	Yes [543,566]	Yes [566]	It can be used as an insulator [543]
9.0	PVA	Organic	[C2H4O]n	Hyperplastic [567]	Yes [568]	Yes [569]	It can be used as an insulator [570]
10.0	Silk	Organic	-	6100/19.55 [571]	Yes [572,573]	Yes [574]	Mechanical properties are based on B. Mori Silk. Elongation value is at the break. Silk properties can be tuned when blending with other materials [575] It can be used as an insulator [576] or substrate [577]
11.0	Shellac	Organic	-	Rheologic [578,579]	Yes [580]	Yes [581]	It can be used as an insulator [581] or substrate [582]
12.0	Sylgard Elastomer (184)	Organic	-	2.4/135 [538]	Yes [583]	-	Elongation value is at ƐMax. Elongation can be tuned when blending with other materials [584]

**Table 7:** List of biocompatible and/or biodegradable materials used in wearable sensors as conductors.

SN	Material	Material Category	Chemical Structure	Conductivity (S/m)	Bio-compatible	Bio-degradable	Note
1.0	Ag NWs	Inorganic	-	6.3 × 107 [585]	Yes [586]	-	They are used as an active material [185] or an electrode [587,588]
2.0	CNTs	Organic	-	103 − 6.7 × 106 [553]	Yes [589]	Yes [590]	CNT conductivity will highly depend on many parameters, such as structure and purity. Likewise, CNTs biodegradability will depend on many parameters, including concentration, size, and functionalization. However, the remaining part of CNTs that stay in the body and accumulate may lead to unknown long-term effects [590].
3.0	Graphene	Inorganic	-	7.095 × 104 [591]	Yes [592]	-	Electrical conductivity is for pristine Graphene. Conductivity will highly depend on many parameters, including the structure and quality of as-made materials. They are used as an excellent conductor in electromechanically based sensors [593], and biosensors, [594], as well as gas separation [595].
4.0	MoS2	Inorganic	-	107 [596]	Yes [597]	-	They are used as an active material, [598] or an electrode [284]
5.0	PANI (doped)	Organic	-	103–104 [599]	Yes [600]	-	Electrical conductivity is influenced by the synthesis conditions such as current density, pH degree, and polymerization time [601] They are used as an active material [602] or an electrode [603]
6.0	PEDOT (doped)	Organic	-	(6.259 ± 1.468) × 105 [604]	Yes [605]	-	Electrical conductivity can be enhanced through chemical treatment with Methanol [606] or PSS content increase reaching the percolation threshold [607] They are used as an active material [608] or an electrode [609]
7.0	PPy (doped)	Organic	-	103–105 [599]	Yes [600]	-	PANI and PPy can be similarly applied in biomedicine when solely their biological properties are considered [600] They are used as an active material [610] or an electrode [611]

## Acronyms and Abbreviations

CB	Carbon Black.
CCE	Capacitive Coupled electrode, which is a non-contact electrode that works on the principle of capacitive charges between the user’s skin and the electrode.
CMC	Carboxymethyl Chitosan.
C-MOF	Carbonized Metal Organic Framework.
CNT	Carbon Nanotube.
CuTCNQ	Copper 7,7,8,8-tetracyano-p-quinodimethane.
CPH	Conducting polymer hydrogel.
CVD	Chemical Vapor Deposition.
DETA	Diethylenetriamine.
DGEBA	Diglycidyl ether of bisphenol A (Epon™ 8132).
DMSO	Dimethyl sulfoxide.
DN Hydrogel	Double Network Hydrogel.
DPP-DTT:PCBM	N-alkyl diketopyrrolo-pyrrole dithienylthienothiophene, and a fullerene derivative, phenyl-C61-butyric acid Methylester.
EmFi	Electromechanical Film.
EPDM	ethylene propylene diene monomer.
EToH	Ethanol.
F	Force.
FFR	Fractional Flow Reserve is a physiological index that invasively measures the ratio between distal and proximal pressure of stenosis at maximum hyperaemia. Also, it is considered a gold standard.
FFR_CT_	Fractional Flow Reserve based on Computed Tomography.
GF	Graphene Foam.
GO	Graphene Oxide.
GNS	Graphene Nanosheet.
GPN	Graphene Porous Network.
GS	Graphene Sponge.
LED	Light Emitting Diode.
LZT	Lead Zirconate Titanate.
MEMS	Micro-electromechanical System.
MOF-5	Zn4O(BDC)3, where BDC =1,4-benzodicarboxylate.
MP	Microparticles.
m-PCL	poly(ɛ-caprolactone).
MWCNT	Multi-Walled Carbon Nanotube.
NITEC	Nitrile imine-mediated tetrazole-ene cycloaddition.
NIR	Near-Infrared.
NP	Nanoparticles.
NS	Nanosheet.
NW	Nanowire.
OLED	Organic Light Emitting Diode.
P	Pressure.
PA	Polyamide.
PAA	Polyacrylic acid.
PANI	Polyaniline.
PANIF	Polyaniline Nanofiber.
PANIPAm	Poly(*N*-isopropylacrylamide).
PBS	Polyborosiloxane
PCL	Polycaprolactone.
PDMS	Polydimethylsiloxane.
PdOx	Palladium Oxides.
PE	Poly(ethelene).
PEDOT: PSS	Poly(3,4-ethylenedioxythiophene):poly(styrene sulfonate).
PEG	Poly(ethylene glycol).
PEN	Polyethylene Naphthalene.
PES	Polyether Sulfone.
PET	Polyethylene Terephthalate.
PETMP	Pentaerythritol tetrakis(3-mercaptopropionate).
PFDTS	1H,1H,2H,2H-Perfluorodecyltrichlorosilane
PGS	Poly(glycerol sebacate).
PHB/PHV	Polyhydroxybutyrate/Polyhydroxyvalerate.
PI	Polyimide.
PLA	Polylactic Acid.
PLGA	Poly(lactic-co-glycolic acid).
PMMA	Polymethylmethacrylate.
POE	Polyolefin Elastomer.
POMaC	Poly(octamethyle nemaleate (anhydride) citrate).
PP	Polypropylene.
PPy	Polypyrrole.
PRF	Passive Radio Frequency.
PSR	Pressure Sensitive Rubber.
PTFE	Polytetrafluoroethylene.
PU	Polyurethane.
PUD	Polyurethane Dispersion.
PVA	Polyvinyl Alcohol.
PVDF	Polyvinylidene fluoride.
PVDF-HFP	Poly(vinylidene fluoride)-co-Hexafluoropropylene.
P(VDF-TrFe)	Poly(vinylidene fluoride-co-trifluoroethylene).
rGO	Reduced Graphene Oxide.
SEM	Scanning Electron Microscope.
sh-crl-PU	Disulfide-cross-linked polyurethane.
SP	Spiropyran
S-R	Self -Resonant.
SWCNT	Single-Walled Carbon Nanotube.
TAA	Terephthalaldehyde.
T-CVD	Thermal -Chemical Vapor Deposition.
TEMPO	2, 2, 6, 6-tetrametylpiperidine-1-oxyl.
TETA	triethylenetetramine.
TOCNF	TEMPO-oxidized cellulose nanofiber.
TPU	Thermoplastic polyurethane.
Triton X-100	Polyethylene glycol p-(1,1,3,3-tetramethylbutyl)-phenyl ether.
TREN	Tris(2-aminoethyl)amine.
TTT	1,3,5-triallyl-1,3,5-triazine-2,4,6(1H,3H,5H)-trione.
VHB	Very High Bond.

## References

[1] World Health Organization (2019). World Health Organization Cardiovascular Diseases (CVDs) Report.

[2] (2020). COVID-19 Surveillance Group Characteristics of COVID-19 Patients dying in Italy Report Based on Available Data on 24 March 2020.

[3] World Health Organization (WHO)-China Joint Mission (2020). Report of the WHO-China Joint Mission on Coronavirus Disease 2019 (COVID-19).

[4] Kovacs R.J., Moyer D.V. (2020). Statement on the Need for Increased Access to Telehealth to Combat Community Spread of COVID-19.

[5] Perl T.M., Price C.S. (2020). Managing emerging infectious diseases: Should travel be the fifth vital sign?. Ann. Intern. Med..

[6] Mancia G., Fagard R., Narkiewicz K., Redon J., Zanchetti A., Bohm M., Christiaens T., Cifkova R., De Backer G., Dominiczak A. (2013). 2013 ESH/ESC Guidelines for the management of arterial hypertension: The Task Force for the management of arterial hypertension of the European Society of Hypertension (ESH) and of the European Society of Cardiology (ESC). J. Hypertens..

[7] Bentzon J.F., Otsuka F., Virmani R., Falk E. (2014). Mechanisms of plaque formation and rupture. Circ. Res..

[8] RenJi H. Early Detection of Cardiac Impairment and Prediction of RV Hypertrophy in Patients With CTD. https://ClinicalTrials.gov/show/NCT04297371.

[9] Sierra C., de la Sierra A. (2008). Early detection and management of the high-risk patient with elevated blood pressure. Vasc. Health Risk Manag..

[10] Handler J. (2009). The importance of accurate blood pressure measurement. Perm. J..

[11] Poncette A.S., Spies C., Mosch L., Schieler M., Weber-Carstens S., Krampe H., Balzer F. (2019). Clinical Requirements of Future Patient Monitoring in the Intensive Care Unit: Qualitative Study. JMIR Med. Inform..

[12] Liu J., Geng Z., Fan Z., Liu J., Chen H. (2019). Point-of-care testing based on smartphone: The current state-of-the-art (2017–2018). Biosens. Bioelectron..

[13] Zhou Z., Padgett S., Cai Z., Conta G., Wu Y., He Q., Zhang S., Sun C., Liu J., Fan E. (2020). Single-layered ultra-soft washable smart textiles for all-around ballistocardiograph, respiration, and posture monitoring during sleep. Biosens. Bioelectron..

[14] Lisi F., Peterson J.R., Gooding J.J. (2020). The application of personal glucose meters as universal point-of-care diagnostic tools. Biosens. Bioelectron..

[15] Shandilya R., Bhargava A., Bunkar N., Tiwari R., Goryacheva I.Y., Mishra P.K. (2019). Nanobiosensors: Point-of-care approaches for cancer diagnostics. Biosens. Bioelectron..

[16] Xu D., Huang X., Guo J., Ma X. (2018). Automatic smartphone-based microfluidic biosensor system at the point of care. Biosens. Bioelectron..

[17] Escobedo P., Erenas M.M., Martinez-Olmos A., Carvajal M.A., Gonzalez-Chocano S., Capitan-Vallvey L.F., Palma A.J. (2019). General-purpose passive wireless point-of-care platform based on smartphone. Biosens. Bioelectron..

[18] Wang T., Mei Q., Tao Z., Wu H., Zhao M., Wang S., Liu Y. (2020). A smartphone-integrated ratiometric fluorescence sensing platform for visual and quantitative point-of-care testing of tetracycline. Biosens. Bioelectron..

[19] Kurbanoglu S., Ozkan S.A., Merkoci A. (2017). Nanomaterials-based enzyme electrochemical biosensors operating through inhibition for biosensing applications. Biosens. Bioelectron..

[20] Song Y., Min J., Gao W. (2019). Wearable and Implantable Electronics: Moving toward Precision Therapy. ACS Nano.

[21] Iftikhar Z., Lahdenoja O., Jafari Tadi M., Hurnanen T., Vasankari T., Kiviniemi T., Airaksinen J., Koivisto T., Pänkäälä M. (2018). Multiclass classifier based cardiovascular condition detection using smartphone mechanocardiography. Sci. Rep..

[22] Pfeiffer S. (2017). The Vision of Industrie 4.0 in the making-a case of future told, tamed, and traded. Nanoethics.

[23] Schwab K. (2016). The Fourth Industrial Revolution.

[24] Topol E.J. (2019). High-performance medicine: The convergence of human and artificial intelligence. Nat. Med..

[25] Chrysant S.G. (2011). A new paradigm in the treatment of the cardiovascular disease continuum: Focus on prevention. Hippokratia.

[26] Engel J., van der Wulp I., Poldervaart J.M., Reitsma J.B., de Bruijne M.C., Wagner C. (2015). Clinical decision-making of cardiologists regarding admission and treatment of patients with suspected unstable angina or non-ST-elevation myocardial infarction: Protocol of a clinical vignette study. BMJ Open.

[27] Karunathilake S.P., Ganegoda G.U. (2018). Secondary prevention of cardiovascular diseases and application of technology for early diagnosis. Biomed. Res. Int..

[28] McGill H.C., McMahan C.A., Gidding S.S. (2008). Preventing heart disease in the 21st century: Implications of the pathobiological determinants of Atherosclerosis in youth (PDAY) study. Circulation.

[29] D’Addona D.M., Rongo R., Teti R., Martina R. (2018). Bio-compatible cyber-physical system for cloud-based customizable sensor monitoring of pressure conditions. Proc. CIRP.

[30] Seminara L., Pinna L., Ibrahim A., Noli L., Capurro M., Caviglia S., Gastaldo P., Valle M. (2014). Electronic Skin: Achievements, Issues and Trends. Proc. Technol..

[31] Shimonomura K. (2019). tactile image sensors employing camera: A review. Sensors.

[32] Xu S., Jayaraman A., Rogers J.A. (2019). Skin sensors are the future of health care. Nature.

[33] Heikenfeld J., Jajack A., Feldman B., Granger S.W., Gaitonde S., Begtrup G., Katchman B.A. (2019). Accessing analytes in biofluids for peripheral biochemical monitoring. Nat. Biotechnol..

[34] Chung H.U., Kim B.H., Lee J.Y., Lee J., Xie Z., Ibler E.M., Lee K., Banks A., Jeong J.Y., Kim J. (2019). Binodal, wireless epidermal electronic systems with in-sensor analytics for neonatal intensive care. Science.

[35] Yao S., Swetha P., Zhu Y. (2018). Nanomaterial-enabled wearable sensors for healthcare. Adv. Healthcare Mater..

[36] Ray T.R., Choi J., Bandodkar A.J., Krishnan S., Gutruf P., Tian L., Ghaffari R., Rogers J.A. (2019). Bio-Integrated wearable systems: A comprehensive review. Chem. Rev..

[37] Yetisen A.K., Martinez-Hurtado J.L., Unal B., Khademhosseini A., Butt H. (2018). Wearables in medicine. Adv. Mater..

[38] Dooley E.E., Golaszewski N.M., Bartholomew J.B. (2017). Estimating Accuracy at exercise intensities: A comparative study of self-monitoring heart rate and physical activity wearable devices. JMIR M.Health UHealth.

[39] Izmailova E.S., Wagner J.A., Perakslis E.D. (2018). Wearable devices in clinical trials: Hype and hypothesis. Clin. Pharmacol. Ther..

[40] Bent B., Goldstein B.A., Kibbe W.A., Dunn J.P. (2020). Investigating sources of inaccuracy in wearable optical heart rate sensors. NPJ Digit. Med..

[41] Kaisti M., Panula T., Leppanen J., Punkkinen R., Jafari Tadi M., Vasankari T., Jaakkola S., Kiviniemi T., Airaksinen J., Kostiainen P. (2019). Clinical assessment of a non-invasive wearable MEMS pressure sensor array for monitoring of arterial pulse waveform, heart rate and detection of atrial fibrillation. NPJ Digit. Med..

[42] Duking P., Fuss F.K., Holmberg H.C., Sperlich B. (2018). Recommendations for assessment of the reliability, sensitivity, and validity of data provided by wearable sensors designed for monitoring physical activity. JMIR MHealth UHealth.

[43] Magder S. (2018). The meaning of blood pressure. Crit. Care.

[44] Vlachopoulos C., O’Rourke M., Nichols W.W. (2011). McDonald’s Blood Flow in Arteries: Theoretical, Experimental and Clinical Principles.

[45] Kleinstreuer C. (2006). Biofluid Dynamics: Principles And Selected Applications.

[46] Belz G.G. (1995). Elastic properties and Windkessel function of the human aorta. Cardiovasc. Drugs Ther..

[47] Ogedegbe G., Pickering T. (2010). Principles and techniques of blood pressure measurement. Cardiol. Clin..

[48] Kim C.S., Ober S.L., McMurtry M.S., Finegan B.A., Inan O.T., Mukkamala R., Hahn J.O. (2016). Ballistocardiogram: Mechanism and potential for unobtrusive cardiovascular health monitoring. Sci. Rep..

[49] Varghees V.N., Ramachandran K.I. (2014). A novel heart sound activity detection framework for automated heart sound analysis. Biomed. Sign. Process. Control.

[50] Chowdhury M.H., Cheung R.C.C. (2019). Reconfigurable architecture for multi-lead ecg signal compression with high-frequency noise reduction. Sci. Rep..

[51] McEniery C.M., Cockcroft J.R., Roman M.J., Franklin S.S., Wilkinson I.B. (2014). Central blood pressure: Current evidence and clinical importance. Eur. Heart J..

[52] Romagnoli S., Ricci Z., Quattrone D., Tofani L., Tujjar O., Villa G., Romano S.M., De Gaudio A.R. (2014). Accuracy of invasive arterial pressure monitoring in cardiovascular patients: An observational study. Crit. Care.

[53] Crystal G.J., Assaad S.I., Heerdt P.M., Hemmings H.C., Egan T.D. (2019). Cardiovascular Physiology. Pharmacology and Physiology for Anesthesia.

[54] Gaukroger P.B., Roberts J.G., Manners T.A. (1988). Infusion thrombophlebitis: A prospective comparison of 645 Vialon and Teflon cannulae in anaesthetic and postoperative use. Anaesth. Intens. Care.

[55] Pizzoferrato A., Arciola C.R., Cenni E., Ciapetti G., Sassi S. (1995). In vitro biocompatibility of a polyurethane catheter after deposition of fluorinated film. Biomaterials.

[56] Zhang L., Cao S., Marsh N., Ray-Barruel G., Flynn J., Larsen E., Rickard C.M. (2016). Infection risks associated with peripheral vascular catheters. J. Infect. Prev..

[57] Lambert J.M., Lee M.-S., Taller R.A., Solomon D.D. (1991). Medical grade tubing: Criteria for catheter applications. J. Vinyl Addit. Technol..

[58] Cohen A.B., Dagli M., Stavropoulos S.W., Mondschein J.I., Soulen M.C., Shlansky-Goldberg R.D., Solomon J.A., Chittams J.L., Trerotola S.O. (2011). Silicone and polyurethane tunneled infusion catheters: A comparison of durability and breakage rates. J. Vasc. Interv. Radiol..

[59] Wall C., Moore J., Thachil J. (2016). Catheter-related thrombosis: A practical approach. J. Intens. Care Soc..

[60] Auffan M., Santaella C., Thiéry A., Paillès C., Rose J., Achouak W., Thill A., Masion A., Wiesner M., Bottero J.-Y., Bhushan B. (2012). Electrostatic MEMS microphones. Encyclopedia of Nanotechnology.

[61] Meena K.V., Mathew R., Sankar A.R. (2017). Design and optimization of a three-terminal piezoresistive pressure sensor for catheter based in vivo biomedical applications. Biomed. Phys. Eng. Express.

[62] Allen H., Ramzan K., Knutti J., Withers S. (2011). A novel ultra-miniature catheter tip pressure sensor fabricated using silicon and glass thinning techniques. MRS Proc..

[63] Hasenkamp W., Forchelet D., Pataky K., Villard J., Van Lintel H., Bertsch A., Wang Q., Renaud P. (2012). Polyimide/SU-8 catheter-tip MEMS gauge pressure sensor. Biomed. Microdev..

[64] Pandya H.J., Sheng J., Desai J.P. (2017). MEMS-based flexible force sensor for tri-axial catheter contact force measurement. J. Microelectromech. Syst..

[65] Dario P., De Rossi D., Bedini R., Francesconi R., Trivella M.G. (2011). PVF2catheter-tip transducers for pressure, sound and flow measurements. Ferroelectrics.

[66] Boutry C.M., Beker L., Kaizawa Y., Vassos C., Tran H., Hinckley A.C., Pfattner R., Niu S., Li J., Claverie J. (2019). Biodegradable and flexible arterial-pulse sensor for the wireless monitoring of blood flow. Nat. Biomed. Eng..

[67] Qingsong X., Guoxing W., Zhengchun P. Machine Learning Methods for Real-Time Blood Pressure Measurement Based on Photoplethysmography. Proceedings of the 2018 IEEE 23rd International Conference on Digital Signal Processing (DSP) Digital Signal Processing (DSP).

[68] Versi E. (1992). Gold standard is an appropriate term. BMJ.

[69] Pour-Ghaz I., Manolukas T., Foray N., Raja J., Rawal A., Ibebuogu U.N., Khouzam R.N. (2019). Accuracy of non-invasive and minimally invasive hemodynamic monitoring: Where do we stand?. Ann. Transl. Med..

[70] Murphy O.H., Bahmanyar M.R., Borghi A., McLeod C.N., Navaratnarajah M., Yacoub M.H., Toumazou C. (2013). Continuous in vivo blood pressure measurements using a fully implantable wireless SAW sensor. Biomed. Microdev..

[71] Melki S., Todani A., Cherfan G. (2014). An implantable intraocular pressure transducer: Initial safety outcomes. JAMA Ophthalmol..

[72] Kawoos U., McCarron R.M., Auker C.R., Chavko M. (2015). Advances in intracranial pressure monitoring and its significance in managing traumatic brain injury. Int. J. Mol. Sci..

[73] Chen X., Brox D., Assadsangabi B., Mohamed Ali M.S., Takahata K. (2017). A stainless-steel-based implantable pressure sensor chip and its integration by microwelding. Sens. Actuators A Phys..

[74] Shin J., Liu Z., Bai W., Liu Y., Yan Y., Xue Y., Kandela I., Pezhouh M., MacEwan M.R., Huang Y. (2019). Bioresorbable optical sensor systems for monitoring of intracranial pressure and temperature. Sci. Adv..

[75] Potkay J.A. (2008). Long term, implantable blood pressure monitoring systems. Biomed. Microdev..

[76] Benmira A., Perez-Martin A., Schuster I., Aichoun I., Coudray S., Bereksi-Reguig F., Dauzat M. (2016). From Korotkoff and Marey to automatic non-invasive oscillometric blood pressure measurement: Does easiness come with reliability?. Exp. Rev. Med. Dev..

[77] Raamat R., Talts K.J.J., Kivastik J. A model-based retrospective analysis of the fixed-ratio oscillometric blood pressure measurement. Proceedings of the 13th IEEE International Conference on BioInformatics and BioEngineering (IEEE BIBE 2013).

[78] Dinesh S., M B., Charles F., Upendra K., Subramaniam N., Jamshed D. (2010). Palpatory method of measuring diastolic blood pressure. J. Anaesthesiol. Clin. Pharmacol..

[79] Odagiri T., Morita T., Yamauchi T., Imai K., Tei Y., Inoue S. (2014). Convenient measurement of systolic pressure: The reliability and validity of manual radial pulse pressure measurement. J. Palliat. Med..

[80] International Organization for Standardization (2018). Non-invasive Sphygmomanometers-Part 2: Clinical Investigation of Intermittent Automated Measurement Type.

[81] Ringrose J.S., McLean D., Ao P., Yousefi F., Sankaralingam S., Millay J., Padwal R. (2016). Effect of cuff design on auscultatory and oscillometric blood pressure measurements. Am. J. Hypertens..

[82] Bilo G., Sala O., Perego C., Faini A., Gao L., Gluszewska A., Ochoa J.E., Pellegrini D., Lonati L.M., Parati G. (2017). Impact of cuff positioning on blood pressure measurement accuracy: May a specially designed cuff make a difference?. Hypertens. Res..

[83] O’Brien E. (1996). Review: A century of confusion; which bladder for accurate blood pressure measurement?. J. Hum. Hypertens..

[84] Tochikubo O., Watanabe J., Hanada K., Miyajima E., Kimura K. (2001). A new double cuff sphygmotonometer for accurate blood pressure measurement. Hypertens. Res..

[85] Brown M.A., Buddle M.L., Whitworth J.A. (1993). Measurement of blood pressure during pregnancy: Evaluation of the ‘TriCUFF’. Aust. N. Z. J. Obstet. Gynaecol..

[86] Bonso E., Saladini F., Zanier A., Benetti E., Dorigatti F., Palatini P. (2010). Accuracy of a single rigid conical cuff with standard-size bladder coupled to an automatic oscillometric device over a wide range of arm circumferences. Hypertens. Res..

[87] Duffy M.K., Williams M. (1992). Blood Pressure Cuff and to a Method of Making the Same. U.S. Patent.

[88] Ledford J., Drake R., Ellenburg L., Jarvis G., Edward L.P. (1998). Bladderless Blood Pressure Cuff. U.S. Patent.

[89] Garrett R.J. (1994). Disposable Medical Pressure Cuffs and Method of Production. U.S. Patent.

[90] Vivenzio Ian R.L., Edwards I.K., Lia R.A., Perkins J., Karla S.R. (2014). Recyclable or Biodegradable Blood Pressure Cuff. U.S. Patent.

[91] Li H., Bao H., Bok K.X., Lee C.Y., Li B., Zin M.T., Kang L. (2016). High durability and low toxicity antimicrobial coatings fabricated by quaternary ammonium silane copolymers. Biomater. Sci..

[92] McCaughey E., Higgins T., Shlisky T. (2010). Antimicrobial Blood Pressure Cuff Liner. U.S. Patent.

[93] Deselle C.T., Durgag K., Paul B., Gunn V., Pendleton B., Provonchee R. (2014). Blood Pressure Cuff Shield Incorporating Antimicrobial Technology. U.S. Patent.

[94] De Smedt S. (2015). Noninvasive intraocular pressure monitoring: Current insights. Clin. Ophthalmol..

[95] Drzewiecki G., Krishna G., Katta H. (2019). Method of deflection corrected tonometry with phantom vessel experiments. Comput. Biol. Med..

[96] Garcia-Ortiz L., Recio-Rodriguez J.I., Agudo-Conde C., Maderuelo-Fernandez J.A., Patino-Alonso M.C., de Cabo-Laso A., Rodriguez-Martin C., Gonzalez-Sanchez J., Rodriguez-Sanchez E., Gomez-Marcos M.A. (2018). Noninvasive validation of central and peripheral augmentation index estimated by a novel wrist-worn tonometer. J. Hypertens..

[97] Hirano H., Fukuchi T., Kurita Y., Kandori A., Sano Y., Nakamura R., Saeki N., Kawamoto M., Yoshizumi M., Tsuji T. (2012). Development of a palpable carotid pulse pressure sensor using electromagnetic induction. IEEJ Trans. Electron. Inform. Syst..

[98] Okafor K.C., Brandt J.D. (2015). Measuring intraocular pressure. Curr. Opin. Ophthalmol..

[99] Ozcura F., Yildirim N., Sahin A., Colak E. (2015). Comparison of Goldmann applanation tonometry, rebound tonometry and dynamic contour tonometry in normal and glaucomatous eyes. Int. J. Ophthalmol..

[100] Leonardi M., Leuenberger P., Bertrand D., Bertsch A., Renaud P. (2003). Digest of Technical Papers (Cat. No.03TH8664). A Soft Contact Lens with a MEMS Strain Gage Embedded For Intraocular Pressure Monitoring, Proceedings of the Transducers 03, 12th International Conference on Solid-State Sensors, Actuators and Microsystems, Boston, MA, USA, 8–12 June 2003.

[101] Chen G.Z., Chan I.S., Leung L.K., Lam D.C. (2014). Soft wearable contact lens sensor for continuous intraocular pressure monitoring. Med. Eng. Phys..

[102] Zhang Y., Chen Y., Man T., Huang D., Li X., Zhu H., Li Z. (2019). High resolution non-invasive intraocular pressure monitoring by use of graphene woven fabrics on contact lens. Microsyst. Nanoeng..

[103] Lee B., Jeong J., Kim J., Kim B., Chun K. (2014). Cantilever arrayed blood pressure sensor for arterial applanation tonometry. IET Nanobiotechnol..

[104] Roh D., Han S., Park J., Shin H. (2019). Development of a multi-array pressure sensor module for radial artery pulse wave measurement. Sensors.

[105] Agnoletti D., Millasseau S.C., Topouchian J., Zhang Y., Safar M.E., Blacher J. (2014). Pulse wave analysis with two tonometric devices: A comparison study. Physiol. Meas..

[106] Hansen S., Staber M. (2006). Oscillometric blood pressure measurement used for calibration of the arterial tonometry method contributes significantly to error. Eur. J. Anaesthesiol..

[107] Uemura K., Kawada T., Sugimachi M. (2019). A novel minimally occlusive cuff method utilizing ultrasound vascular imaging for stress-free blood pressure measurement: A-proof-of-concept study. IEEE Trans. Biomed. Eng..

[108] Saugel B., Cecconi M., Hajjar L.A. (2019). Noninvasive cardiac output monitoring in cardiothoracic surgery patients: Available methods and future directions. J. Cardiothorac. Vasc. Anesth..

[109] Saugel B., Dueck R., Wagner J.Y. (2014). Measurement of blood pressure. Best Pract. Res. Clin. Anaesthesiol..

[110] Gerdt D.W., Adkins C., Baruch M. (2012). Hydrostatic Finger Cuff for Blood Wave Formanalysis And Dagnostic Support. U.S. Patent.

[111] Cline R.L., Rosthauser J.W., Markusch P.H. (2000). Removable Polyurethane Adhesives with Improved Temperature Resistance Properties. U.S. Patent.

[112] Huber C., Grüllenberger R., Fortin J. (2011). Disposable and Detachable Sensor For Continuous Non-Invasive Arterial Blood Pressure Monitoring. U.S. Patent.

[113] Westerhof B., Schraa O., Van Groeningen C.J.E., Li P. (2018). Self Closing Finger Cuff. U.S. Patent.

[114] Edwards Lifesciences, Edwards Lifesciences, ClearSight System (2018). Innovation for Noninvasive Hemodynamic Management.

[115] Hertzman A.B. (1938). The blood supply of various skin areas as estimated by the photoelectric plethysmograph. Am. J. Physiol. Cell Physiol..

[116] Ding X.R., Zhao N., Yang G.Z., Pettigrew R.I., Lo B., Miao F., Li Y., Liu J., Zhang Y.T. (2016). Continuous blood pressure measurement from invasive to unobtrusive: Celebration of 200th birth anniversary of Carl Ludwig. IEEE J. Biomed. Health Inform..

[117] Cluff K., Becker R., Jayakumar B., Han K., Condon E., Dudley K., Szatkowski G., Pipinos I.I., Amick R.Z., Patterson J. (2018). Passive wearable skin patch sensor measures limb hemodynamics based on electromagnetic resonance. IEEE Trans. Biomed. Eng..

[118] Birch A.A., Morris S.L. (2003). Do the Finapres and Colin radial artery tonometer measure the same blood pressure changes following deflation of thigh cuffs?. Physiol. Meas..

[119] Hohn A., Defosse J.M., Becker S., Steffen C., Wappler F., Sakka S.G. (2013). Non-invasive continuous arterial pressure monitoring with Nexfin does not sufficiently replace invasive measurements in critically ill patients. Br. J. Anaesth..

[120] Berkelmans G.F.N., Kuipers S., Westerhof B.E., Spoelstra-de Man A.M.E., Smulders Y.M. (2018). Comparing volume-clamp method and intra-arterial blood pressure measurements in patients with atrial fibrillation admitted to the intensive or medium care unit. J. Clin. Monit. Comput..

[121] Xiong G., Figueroa C.A., Xiao N., Taylor C.A. (2011). Simulation of blood flow in deformable vessels using subject-specific geometry and spatially varying wall properties. Int. J. Numer. Method Biomed. Eng..

[122] Davies J.I., Struthers A.D. (2005). Beyond blood pressure: Pulse wave analysis--a better way of assessing cardiovascular risk?. Future Cardiol..

[123] De Cort S.C., Innes J.A., Barstow T.J., Guz A. (1991). Cardiac output, oxygen consumption and arteriovenous oxygen difference following a sudden rise in exercise level in humans. J. Physiol..

[124] Alruwaili F., Cluff K., Griffith J., Farhoud H. (2018). Passive self resonant skin patch sensor to monitor cardiac intraventricular stroke volume using electromagnetic properties of blood. IEEE J. Transl. Eng. Health Med..

[125] Wang C., Li X., Hu H., Zhang L., Huang Z., Lin M., Zhang Z., Yin Z., Huang B., Gong H. (2018). Monitoring of the central blood pressure waveform via a conformal ultrasonic device. Nat. Biomed. Eng..

[126] Task Force M., Montalescot G., Sechtem U., Achenbach S., Andreotti F., Arden C., Budaj A., Bugiardini R., Crea F., Cuisset T. (2013). 2013 ESC guidelines on the management of stable coronary artery disease: The Task Force on the management of stable coronary artery disease of the European Society of Cardiology. Eur. Heart J..

[127] Ma Y., Choi J., Hourlier-Fargette A., Xue Y., Chung H.U., Lee J.Y., Wang X., Xie Z., Kang D., Wang H. (2018). Relation between blood pressure and pulse wave velocity for human arteries. Proc. Natl. Acad. Sci. USA.

[128] Meijboom W.B., Van Mieghem C.A., van Pelt N., Weustink A., Pugliese F., Mollet N.R., Boersma E., Regar E., van Geuns R.J., de Jaegere P.J. (2008). Comprehensive assessment of coronary artery stenoses: Computed tomography coronary angiography versus conventional coronary angiography and correlation with fractional flow reserve in patients with stable angina. J. Am. Coll. Cardiol..

[129] Douglas P.S., Pontone G., Hlatky M.A., Patel M.R., Norgaard B.L., Byrne R.A., Curzen N., Purcell I., Gutberlet M., Rioufol G. (2015). Clinical outcomes of fractional flow reserve by computed tomographic angiography-guided diagnostic strategies vs. usual care in patients with suspected coronary artery disease: The prospective longitudinal trial of FFR(CT): Outcome and resource impacts study. Eur. Heart J..

[130] Neumann F.J., Sousa-Uva M., Ahlsson A., Alfonso F., Banning A.P., Benedetto U., Byrne R.A., Collet J.P., Falk V., Head S.J. (2019). 2018 ESC/EACTS Guidelines on myocardial revascularization. Eur. Heart J..

[131] Norgaard B.L., Leipsic J., Gaur S., Seneviratne S., Ko B.S., Ito H., Jensen J.M., Mauri L., De Bruyne B., Bezerra H. (2014). Diagnostic performance of noninvasive fractional flow reserve derived from coronary computed tomography angiography in suspected coronary artery disease: The NXT trial (Analysis of coronary blood flow using ct angiography: Next steps). J. Am. Coll. Cardiol..

[132] Cook C.M., Petraco R., Shun-Shin M.J., Ahmad Y., Nijjer S., Al-Lamee R., Kikuta Y., Shiono Y., Mayet J., Francis D.P. (2017). Diagnostic Accuracy of computed tomography-derived fractional flow reserve: A systematic review. JAMA Cardiol..

[133] Finlay D.D., Nugent C.D., Donnelly M.P., McCullagh P.J., Black N.D. (2008). Optimal electrocardiographic lead systems: Practical scenarios in smart clothing and wearable health systems. IEEE Trans. Inf. Technol. Biomed..

[134] Baek J.-Y., An J.-H., Choi J.-M., Park K.-S., Lee S.-H. (2008). Flexible polymeric dry electrodes for the long-term monitoring of ECG. Sens. Actuators A Phys..

[135] Haghdoost F., Mottaghitalab V., Haghi A.K. (2015). Comfortable textile-based electrode for wearable electrocardiogram. Sens. Rev..

[136] Chlaihawi A.A., Narakathu B.B., Emamian S., Bazuin B.J., Atashbar M.Z. (2018). Development of printed and flexible dry ECG electrodes. Sens. Bio-Sens. Res..

[137] Pani D., Dessi A., Saenz-Cogollo J.F., Barabino G., Fraboni B., Bonfiglio A. (2016). fully textile, pedot:pss based electrodes for wearable ecg monitoring systems. IEEE Trans. Biomed. Eng..

[138] Stauffer F., Thielen M., Sauter C., Chardonnens S., Bachmann S., Tybrandt K., Peters C., Hierold C., Voros J. (2018). Skin conformal polymer electrodes for clinical ECG and EEG recordings. Adv. Healthcare Mater..

[139] Chen Y.H., Op de Beeck M., Vanderheyden L., Carrette E., Mihajlovic V., Vanstreels K., Grundlehner B., Gadeyne S., Boon P., Van Hoof C. (2014). Soft, comfortable polymer dry electrodes for high quality ECG and EEG recording. Sensors.

[140] Joosten A., Desebbe O., Suehiro K., Murphy L.S., Essiet M., Alexander B., Fischer M.O., Barvais L., Van Obbergh L., Maucort-Boulch D. (2017). Accuracy and precision of non-invasive cardiac output monitoring devices in perioperative medicine: A systematic review and meta-analysisdagger. Br. J. Anaesth..

[141] Mukkamala R., Hahn J.O., Inan O.T., Mestha L.K., Kim C.S., Toreyin H., Kyal S. (2015). Toward Ubiquitous Blood Pressure Monitoring via Pulse Transit Time: Theory and Practice. IEEE Trans. Biomed. Eng..

[142] Ding X., Zhang Y.T. (2019). Pulse transit time technique for cuffless unobtrusive blood pressure measurement: From theory to algorithm. Biomed. Eng. Lett..

[143] Ale I.S., Maibacht H.A. (2010). Diagnostic approach in allergic and irritant contact dermatitis. Exp. Rev. Clin. Immunol..

[144] Brasch J., Becker D., Aberer W., Bircher A., Kranke B., Jung K., Przybilla B., Biedermann T., Werfel T., John S.M. (2014). Guideline contact dermatitis: S1-Guidelines of the German Contact Allergy Group (DKG) of the German Dermatology Society (DDG), the Information Network of Dermatological Clinics (IVDK), the German Society for Allergology and Clinical Immunology (DGAKI), the Working Group for Occupational and Environmental Dermatology (ABD) of the DDG, the Medical Association of German Allergologists (AeDA), the Professional Association of German Dermatologists (BVDD) and the DDG. Allergol. J. Int..

[145] Jin H., Abu-Raya Y.S., Haick H. (2017). Advanced materials for health monitoring with skin-based wearable devices. Adv. Healthcare Mater..

[146] Zink M.D., Bruser C., Stuben B.O., Napp A., Stohr R., Leonhardt S., Marx N., Mischke K., Schulz J.B., Schiefer J. (2017). Unobtrusive nocturnal heartbeat monitoring by a ballistocardiographic sensor in patients with sleep disordered breathing. Sci. Rep..

[147] Lee K.J., Roh J., Cho D., Hyeong J., Kim S. (2019). A Chair-based unconstrained/nonintrusive cuffless blood pressure monitoring system using a two-channel ballistocardiogram. Sensors.

[148] Ha T., Tran J., Liu S., Jang H., Jeong H., Mitbander R., Huh H., Qiu Y., Duong J., Wang R.L. (2019). A chest-laminated ultrathin and stretchable e-tattoo for the measurement of electrocardiogram, seismocardiogram, and cardiac time intervals. Adv. Sci. (Weinh).

[149] Lu N., Ameri S., Ha T., Nicolini L., Stier A., Wang P. (2017). Epidermal Electronic Systems for Sensing and Therapy.

[150] Pinheiro E., Postolache O., Girão P. (2012). Study on Ballistocardiogram Acquisition in a Moving Wheelchair with Embedded Sensors. Metrol. Meas. Syst..

[151] Heise D., Rosales L., Skubic M., Devaney M.J. Refinement and Evaluation of a Hydraulic Bed Sensor. Proceedings of the 2011 Annual International Conference of the IEEE Engineering in Medicine and Biology Society.

[152] Chiu Y.-Y., Lin W.-Y., Wang H.-Y., Huang S.-B., Wu M.-H. (2013). Development of a piezoelectric polyvinylidene fluoride (PVDF) polymer-based sensor patch for simultaneous heartbeat and respiration monitoring. Sens. Actuators A Phys..

[153] Marquez J.C., Rempfler M., Seoane F., Lindecrantz K. (2019). Textrode-enabled transthoracic electrical bioimpedance measurements–towards wearable applications of impedance cardiography. J. Electric. Bioimpedance.

[154] Searle A., Kirkup L. (2000). A direct comparison of wet, dry and insulating bioelectric recording electrodes. Physiol. Meas..

[155] Ramasamy S., Balan A. (2018). Wearable sensors for ECG measurement: A review. Sens. Rev..

[156] Merritt C.R., Nagle H.T., Grant E. (2009). Textile-based capacitive sensors for respiration monitoring. IEEE Sens. J..

[157] Sharma P., Imtiaz S.A., Rodriguez-Villegas E. (2019). Acoustic sensing as a novel wearable approach for cardiac monitoring at the wrist. Sci. Rep..

[158] Griffith J., Cluff K., Eckerman B., Aldrich J., Becker R., Moore-Jansen P., Patterson J. (2018). Non-invasive electromagnetic skin patch sensor to measure intracranial fluid-volume shifts. Sensors.

[159] Kaniusas E., Pfutzner H., Mehnen L., Kosel J., Tellez-Blanco C., Varoneckas G., Alonderis A., Meydan T., Vazquez M., Rohn M. (2006). Method for continuous nondisturbing monitoring of blood pressure by magnetoelastic skin curvature sensor and ECG. IEEE Sens. J..

[160] Pogue B.W., Poplack S.P., McBride T.O., Wells W.A., Osterman K.S., Osterberg U.L., Paulsen K.D. (2001). Quantitative hemoglobin tomography with diffuse near-infrared spectroscopy: Pilot results in the breast. Radiology.

[161] Xing X., Sun M. (2016). Optical blood pressure estimation with photoplethysmography and FFT-based neural networks. Biomed. Opt. Express.

[162] Kamal A.A., Harness J.B., Irving G., Mearns A.J. (1989). Skin photoplethysmography—A review. Comput. Methods Programs Biomed..

[163] Tamura T., Maeda Y., Sekine M., Yoshida M. (2014). Wearable Photoplethysmographic Sensors—Past and Present. Electronics.

[164] Mendelson Y., Ochs B.D. (1988). Noninvasive pulse oximetry utilizing skin reflectance photoplethysmography. IEEE Trans. Biomed. Eng..

[165] Vazquez K., Cota J., Sifuentes E., Gonzalez R. (2016). High Signal-to-noise ratio phonocardiogram using a shielded pvdf film sensor. IEEE Lat. Am. Trans..

[166] Ding X., Dai W., Luo N., Liu J., Zhao N., Zhang Y. A Flexible Tonoarteriography-Based Body Sensor Network for Cuffless Measurement Of Arterial Blood Pressure. Proceedings of the 2015 IEEE 12th International Conference on Wearable and Implantable Body Sensor Networks (BSN).

[167] Eduardo C., Octavian A., Pedro S. Calibration and validation of homeostasis parameters estimates produced by a DSP embedded in a wheelchair. Proceedings of the 2013 IEEE International Instrumentation and Measurement Technology Conference (I2MTC) Instrumentation and Measurement Technology Conference (I2MTC).

[168] Yang C., Tavassolian N. (2018). Pulse transit time measurement using seismocardiogram, photoplethysmogram, and acoustic recordings: Evaluation and comparison. IEEE J Biomed. Health Inform..

[169] Pang C., Koo J.H., Nguyen A., Caves J.M., Kim M.G., Chortos A., Kim K., Wang P.J., Tok J.B., Bao Z. (2015). Highly skin-conformal microhairy sensor for pulse signal amplification. Adv. Mater..

[170] Luo N., Dai W., Li C., Zhou Z., Lu L., Poon C.C.Y., Chen S.-C., Zhang Y., Zhao N. (2016). Flexible piezoresistive sensor patch enabling ultralow power cuffless blood pressure measurement. Adv. Funct. Mater..

[171] Rendon D.B., Rojas Ojeda J.L., Crespo Foix L.F., Morillo D.S., Fernandez M.A. (2007). Mapping the human body for vibrations using an accelerometer. Conf. Proc. IEEE Eng. Med. Biol. Soc..

[172] Dagdeviren C., Su Y., Joe P., Yona R., Liu Y., Kim Y.S., Huang Y., Damadoran A.R., Xia J., Martin L.W. (2014). Conformable amplified lead zirconate titanate sensors with enhanced piezoelectric response for cutaneous pressure monitoring. Nat. Commun..

[173] Simjanoska M., Gjoreski M., Gams M., Madevska Bogdanova A. (2018). Non-invasive blood pressure estimation from ecg using machine learning techniques. Sensors.

[174] Yazdanian H., Mahnam A., Edrisi M., Esfahani M.A. (2016). Design and implementation of a portable impedance cardiography system for noninvasive stroke volume monitoring. J. Med. Sign. Sens..

[175] Xu H., Liu J., Zhang J., Zhou G., Luo N., Zhao N. (2017). Flexible organic/inorganic hybrid near-infrared photoplethysmogram sensor for cardiovascular monitoring. Adv. Mater..

[176] Choong C.L., Shim M.B., Lee B.S., Jeon S., Ko D.S., Kang T.H., Bae J., Lee S.H., Byun K.E., Im J. (2014). Highly stretchable resistive pressure sensors using a conductive elastomeric composite on a micropyramid array. Adv. Mater..

[177] Stauffer D. (2014). Introduction to Percolation Theory.

[178] Yu H., Huang J. (2015). Design and application of a high sensitivity piezoresistive pressure sensor for low pressure conditions. Sensors.

[179] Kaidarova A., Alsharif N., Oliveira B.N.M., Marengo M., Geraldi N.R., Duarte C.M., Kosel J. (2020). Laser-Printed, Flexible Graphene Pressure Sensors. Glob. Chall..

[180] Lu Y., Tian M., Sun X., Pan N., Chen F., Zhu S., Zhang X., Chen S. (2019). Highly sensitive wearable 3D piezoresistive pressure sensors based on graphene coated isotropic non-woven substrate. Compos. Part A Appl. Sci. Manufact..

[181] Herren B., Saha M.C., Liu Y. (2019). Carbon nanotube-based piezoresistive sensors fabricated by microwave irradiation. Adv. Eng. Mater..

[182] Hu J., Yu J., Li Y., Liao X., Yan X., Li L. (2020). Nano carbon black-based high performance wearable pressure sensors. Nanomaterials.

[183] Chang X., Sun S., Sun S., Liu T., Xiong X., Lei Y., Dong L., Yin Y. (2018). ZnO nanorods/carbon black-based flexible strain sensor for detecting human motions. J. Alloys Comp..

[184] Vuorinen T., Laurila M.-M., Mangayil R., Karp M., Mäntysalo M. (2018). High Resolution E-Jet Printed Temperature Sensor on Artificial Skin.

[185] Choi S., Han S.I., Jung D., Hwang H.J., Lim C., Bae S., Park O.K., Tschabrunn C.M., Lee M., Bae S.Y. (2018). Highly conductive, stretchable and biocompatible Ag-Au core-sheath nanowire composite for wearable and implantable bioelectronics. Nat. Nanotechnol..

[186] Wang Z., Wang S., Zeng J., Ren X., Chee A.J., Yiu B.Y., Chung W.C., Yang Y., Yu A.C., Roberts R.C. (2016). High sensitivity, wearable, piezoresistive pressure sensors based on irregular microhump structures and its applications in body motion sensing. Small.

[187] Zhou Y., He J., Wang H., Qi K., Nan N., You X., Shao W., Wang L., Ding B., Cui S. (2017). Highly sensitive, self-powered and wearable electronic skin based on pressure-sensitive nanofiber woven fabric sensor. Sci. Rep..

[188] Gao Y., Yan C., Huang H., Yang T., Tian G., Xiong D., Chen N., Chu X., Zhong S., Deng W. (2020). Microchannel-confined mxene based flexible piezoresistive multifunctional micro-force sensor. Adv. Funct. Mater..

[189] Li L., Fu X., Chen S., Uzun S., Levitt A.S., Shuck C.E., Han W., Gogotsi Y. (2020). Hydrophobic and stable mxene-polymer pressure sensors for wearable electronics. ACS Appl. Mater. Interfaces.

[190] Luo S., Yang J., Song X., Zhou X., Yu L., Sun T., Yu C., Huang D., Du C., Wei D. (2018). Tunable-Sensitivity flexible pressure sensor based on graphene transparent electrode. Solid-State Electron..

[191] Kang M., Kim J., Jang B., Chae Y., Kim J.H., Ahn J.H. (2017). Graphene-based three-dimensional capacitive touch sensor for wearable electronics. ACS Nano.

[192] Wan S., Bi H., Zhou Y., Xie X., Su S., Yin K., Sun L. (2017). Graphene oxide as high-performance dielectric materials for capacitive pressure sensors. Carbon.

[193] Sahatiya P., Badhulika S. (2017). Eraser-based eco-friendly fabrication of a skin-like large-area matrix of flexible carbon nanotube strain and pressure sensors. Nanotechnology.

[194] Kwon D., Lee T.I., Shim J., Ryu S., Kim M.S., Kim S., Kim T.S., Park I. (2016). Highly sensitive, flexible, and wearable pressure sensor based on a giant piezocapacitive effect of three-dimensional microporous elastomeric dielectric layer. ACS Appl. Mater. Interfaces.

[195] Wan Y., Qiu Z., Huang J., Yang J., Wang Q., Lu P., Yang J., Zhang J., Huang S., Wu Z. (2018). Natural plant materials as dielectric layer for highly sensitive flexible electronic skin. Small.

[196] Chhetry A., Yoon H., Park J.Y. (2017). A flexible and highly sensitive capacitive pressure sensor based on conductive fibers with a microporous dielectric for wearable electronics. J. Mater. Chem. C.

[197] Li W., Xiong L., Pu Y., Quan Y., Li S. (2019). High-Performance paper-based capacitive flexible pressure sensor and its application in human-related measurement. Nanoscale Res. Lett..

[198] Maity K., Garain S., Henkel K., Schmeißer D., Mandal D. (2020). Self-Powered human-health monitoring through aligned pvdf nanofibers interfaced skin-interactive piezoelectric sensor. ACS Appl. Polym. Mater..

[199] Kim H., Kim G., Kim T., Lee S., Kang D., Hwang M.S., Chae Y., Kang S., Lee H., Park H.G. (2018). Transparent, flexible, conformal capacitive pressure sensors with nanoparticles. Small.

[200] Guo Y., Zhong M., Fang Z., Wan P., Yu G. (2019). A Wearable transient pressure sensor made with mxene nanosheets for sensitive broad-range human-machine interfacing. Nano Lett..

[201] Ramuz M., Tee B.C., Tok J.B., Bao Z. (2012). Transparent, optical, pressure-sensitive artificial skin for large-area stretchable electronics. Adv. Mater..

[202] Koyama S., Ishizawa H. (2019). Vital sign measurement using fbg sensor for new wearable sensor development, fiber optic sensing-principle, measurement and applications. Shien-Kuei Liaw.

[203] Dziuda L., Skibniewski F.W., Krej M., Baran P.M. (2013). Fiber Bragg grating-based sensor for monitoring respiration and heart activity during magnetic resonance imaging examinations. J. Biomed. Opt..

[204] Ogawa K., Koyama S., Haseda Y., Fujita K., Ishizawa H., Fujimoto K. (2019). Wireless, portable fiber bragg grating interrogation system employing optical edge filter. Sensors.

[205] Nedoma J., Kepak S., Fajkus M., Cubik J., Siska P., Martinek R., Krupa P. (2018). Magnetic resonance imaging compatible non-invasive fibre-optic sensors based on the bragg gratings and interferometers in the application of monitoring heart and respiration rate of the human body: A comparative study. Sensors.

[206] Lo Presti D., Romano C., Massaroni C., D’Abbraccio J., Massari L., Caponero M.A., Oddo C.M., Formica D., Schena E. (2019). Cardio-Respiratory monitoring in archery using a smart textile based on flexible fiber bragg grating sensors. Sensors.

[207] Yogeswaran N., Navaraj W.T., Gupta S., Liu F., Vinciguerra V., Lorenzelli L., Dahiya R. (2018). Piezoelectric graphene field effect transistor pressure sensors for tactile sensing. Appl. Phys. Lett..

[208] Kotlowski C., Aspermair P., Khan H.U., Reiner-Rozman C., Breu J., Szunerits S., Kim J.-J., Bao Z., Kleber C., Pelosi P. (2018). Electronic biosensing with flexible organic transistor devices. Flexib. Print. Electron..

[209] Viola F.A., Spanu A., Ricci P.C., Bonfiglio A., Cosseddu P. (2018). Ultrathin, flexible and multimodal tactile sensors based on organic field-effect transistors. Sci. Rep..

[210] Wang C., Hwang D., Yu Z., Takei K., Park J., Chen T., Ma B., Javey A. (2013). User-interactive electronic skin for instantaneous pressure visualization. Nat. Mater..

[211] Lee Y.H., Jang M., Lee M.Y., Kweon O.Y., Oh J.H. (2017). Flexible field-effect transistor-type sensors based on conjugated molecules. Chemicals.

[212] Chen J., Wang Z.L. (2017). Reviving vibration energy harvesting and self-powered sensing by a triboelectric nanogenerator. Joule.

[213] Lin Z., Yang J., Li X., Wu Y., Wei W., Liu J., Chen J., Yang J. (2018). Large-Scale and washable smart textiles based on triboelectric nanogenerator arrays for self-powered sleeping monitoring. Adv. Funct. Mater..

[214] Zhang N., Tao C., Fan X., Chen J. (2017). Progress in triboelectric nanogenerators as self-powered smart sensors. J. Mater. Res..

[215] Meng K., Zhao S., Zhou Y., Wu Y., Zhang S., He Q., Wang X., Zhou Z., Fan W., Tan X. (2020). Wireless textile-based sensor system for self-powered personalized health care. Matter.

[216] Yang J., Chen J., Su Y., Jing Q., Li Z., Yi F., Wen X., Wang Z., Wang Z.L. (2015). Eardrum-inspired active sensors for self-powered cardiovascular system characterization and throat-attached anti-interference voice recognition. Adv. Mater..

[217] Bai P., Zhu G., Jing Q., Yang J., Chen J., Su Y., Ma J., Zhang G., Wang Z.L. (2014). Membrane-Based self-powered triboelectric sensors for pressure change detection and its uses in security surveillance and healthcare monitoring. Adv. Funct. Mater..

[218] Lin Z., Chen J., Li X., Zhou Z., Meng K., Wei W., Yang J., Wang Z.L. (2017). Triboelectric nanogenerator enabled body sensor network for self-powered human heart-rate monitoring. ACS Nano.

[219] Yan C., Deng W., Jin L., Yang T., Wang Z., Chu X., Su H., Chen J., Yang W. (2018). Epidermis-Inspired ultrathin 3d cellular sensor array for self-powered biomedical monitoring. ACS Appl. Mater. Interfaces.

[220] Meng K., Chen J., Li X., Wu Y., Fan W., Zhou Z., He Q., Wang X., Fan X., Zhang Y. (2018). Flexible weaving constructed self-powered pressure sensor enabling continuous diagnosis of cardiovascular disease and measurement of cuffless blood pressure. Adv. Funct. Mater..

[221] Lee J.H., Hinchet R., Kim S.K., Kim S., Kim S.-W. (2015). Shape memory polymer-based self-healing triboelectric nanogenerator. Energy Environ. Sci..

[222] Ha M., Park J., Lee Y., Ko H. (2015). Triboelectric generators and sensors for self-powered wearable electronics. ACS Nano.

[223] Fan F.R., Lin L., Zhu G., Wu W., Zhang R., Wang Z.L. (2012). Transparent triboelectric nanogenerators and self-powered pressure sensors based on micropatterned plastic films. Nano Lett..

[224] Wang S., Lin L., Xie Y., Jing Q., Niu S., Wang Z.L. (2013). Sliding-triboelectric nanogenerators based on in-plane charge-separation mechanism. Nano Lett..

[225] Someya T., Bao Z., Malliaras G.G. (2016). The rise of plastic bioelectronics. Nature.

[226] Liu Y., Wang H., Zhao W., Zhang M., Qin H., Xie Y. (2018). Flexible, stretchable sensors for wearable health monitoring: Sensing mechanisms, materials, fabrication strategies and features. Sensors.

[227] Park M., Bok B.G., Ahn J.H., Kim M.S. (2018). Recent advances in tactile sensing technology. Micromachines.

[228] Haddara Y.M., Howlader M.M.R. (2018). Integration of heterogeneous materials for wearable sensors. Polymers.

[229] Lee C., Jug L., Meng E. (2013). High strain biocompatible polydimethylsiloxane-based conductive graphene and multiwalled carbon nanotube nanocomposite strain sensors. Appl. Phys. Lett..

[230] Tao L., Wang D., Tian H., Ju Z., Liu Y., Chen Y., Xie Q., Zhao H., Yang Y., Ren T. In Tunable and Wearable High Performance Strain Sensors Based on Laser Patterned Graphene Flakes. Proceedings of the 2016 IEEE International Electron Devices Meeting (IEDM).

[231] Glennon T., O’Quigley C., McCaul M., Matzeu G., Beirne S., Wallace G.G., Stroiescu F., O’Mahoney N., White P., Diamond D. (2016). ‘SWEATCH’: A wearable platform for harvesting and analysing sweat sodium content. Electroanalysis.

[232] Munje R.D., Muthukumar S., Prasad S. (2017). Lancet-free and label-free diagnostics of glucose in sweat using Zinc Oxide based flexible bioelectronics. Sens. Actuators B Chem..

[233] Kurra N., Kulkarni G.U. (2013). Pencil-on-paper: Electronic devices. Lab. Chip.

[234] Cheng J., Zhou B., Lukowicz P., Seoane F., Varga M., Mehmann A., Chabrecek P., Gaschler W., Goenner K., Horter H. (2017). Textile building blocks: Toward simple, modularized, and standardized smart textile. Smart Text..

[235] Guay P., Gorgutsa S., LaRochelle S., Messaddeq Y. (2017). Wearable contactless respiration sensor based on multi-material fibers integrated into textile. Sensors.

[236] Wei Y., Chen S., Lin Y., Yuan X., Liu L. (2016). Silver nanowires coated on cotton for flexible pressure sensors. J. Mater. Chem. C.

[237] Lei K.F., Lee K.-F., Lee M.-Y. (2012). Development of a flexible PDMS capacitive pressure sensor for plantar pressure measurement. Microelectron. Eng..

[238] Zhang J., Zhou L.J., Zhang H.M., Zhao Z.X., Dong S.L., Wei S., Zhao J., Wang Z.L., Guo B., Hu P.A. (2018). Highly sensitive flexible three-axis tactile sensors based on the interface contact resistance of microstructured graphene. Nanoscale.

[239] Ponnamma D., Sadasivuni K.K., Grohens Y., Guo Q., Thomas S. (2014). Carbon nanotube based elastomer composites – an approach towards multifunctional materials. J. Mater. Chem. C.

[240] Wu X., Han Y., Zhang X., Zhou Z., Lu C. (2016). Large-Area compliant, low-cost, and versatile pressure-sensing platform based on microcrack-designed carbon black @ polyurethane sponge for human-machine interfacing. Adv. Funct. Mater..

[241] Guo S.Z., Qiu K., Meng F., Park S.H., McAlpine M.C. (2017). 3D Printed stretchable tactile sensors. Adv. Mater..

[242] Xia K., Wang C., Jian M., Wang Q., Zhang Y. (2017). CVD growth of fingerprint-like patterned 3D graphene film for an ultrasensitive pressure sensor. Nano Res..

[243] Zhu B., Niu Z., Wang H., Leow W.R., Wang H., Li Y., Zheng L., Wei J., Huo F., Chen X. (2014). Microstructured graphene arrays for highly sensitive flexible tactile sensors. Small.

[244] Lipomi D.J., Vosgueritchian M., Tee B.C., Hellstrom S.L., Lee J.A., Fox C.H., Bao Z. (2011). Skin-like pressure and strain sensors based on transparent elastic films of carbon nanotubes. Nat. Nanotechnol..

[245] Schwartz G., Tee B.C., Mei J., Appleton A.L., Kim D.H., Wang H., Bao Z. (2013). Flexible polymer transistors with high pressure sensitivity for application in electronic skin and health monitoring. Nat. Commun..

[246] Douguet M., Picard C., Savary G., Merlaud F., Loubat-Bouleuc N., Grisel M. (2017). Spreading properties of cosmetic emollients: Use of synthetic skin surface to elucidate structural effect. Colloids Surf. B Biointerfaces.

[247] Yang J., Luo S., Zhou X., Li J., Fu J., Yang W., Wei D. (2019). Flexible, tunable, and ultrasensitive capacitive pressure sensor with microconformal graphene electrodes. ACS Appl. Mater. Interfaces.

[248] Chen M., Luo W., Xu Z., Zhang X., Xie B., Wang G., Han M. (2019). An ultrahigh resolution pressure sensor based on percolative metal nanoparticle arrays. Nat. Commun..

[249] Fu X., Dong H., Zhen Y., Hu W. (2015). Solution-processed large-area nanocrystal arrays of metal-organic frameworks as wearable, ultrasensitive, electronic skin for health monitoring. Small.

[250] Ma Y., Liu N., Li L., Hu X., Zou Z., Wang J., Luo S., Gao Y. (2017). A highly flexible and sensitive piezoresistive sensor based on MXene with greatly changed interlayer distances. Nat. Commun..

[251] Lou Z., Chen S., Wang L., Jiang K., Shen G. (2016). An ultra-sensitive and rapid response speed graphene pressure sensors for electronic skin and health monitoring. Nano Energy.

[252] Spahr M.E., Rothon R., Palsule S. (2016). Carbon Black as a Polymer Filler. Polymers and Polymeric Composites: A Reference Series.

[253] Lekawa-Raus A., Patmore J., Kurzepa L., Bulmer J., Koziol K. (2014). Electrical properties of carbon nanotube based fibers and their future use in electrical wiring. Adv. Funct. Mater..

[254] Tang J., Cao Q., Tulevski G., Jenkins K.A., Nela L., Farmer D.B., Han S.-J. (2018). Flexible CMOS integrated circuits based on carbon nanotubes with sub-10 ns stage delays. Nat. Electron..

[255] Chen J., Yan L. (2018). Effect of carbon nanotube aspect ratio on the thermal and electrical properties of epoxy nanocomposites. Fuller. Nanotubes Carbon Nanostruct..

[256] Tewari A., Gandla S., Bohm S., McNeill C.R., Gupta D. (2018). Highly exfoliated MWNT-RGO ink-wrapped polyurethane foam for piezoresistive pressure sensor applications. ACS Appl. Mater. Interfaces.

[257] Jung S., Kim J.H., Kim J., Choi S., Lee J., Park I., Hyeon T., Kim D.H. (2014). Reverse-micelle-induced porous pressure-sensitive rubber for wearable human-machine interfaces. Adv. Mater..

[258] Park J., Lee Y., Hong J., Ha M., Jung Y.D., Lim H., Kim S.Y., Ko H. (2014). Giant tunneling piezoresistance of composite elastomers with interlocked microdome arrays for ultrasensitive and multimodal electronic skins. ACS Nano.

[259] Jian M., Xia K., Wang Q., Yin Z., Wang H., Wang C., Xie H., Zhang M., Zhang Y. (2017). Flexible and highly sensitive pressure sensors based on bionic hierarchical structures. Adv. Funct. Mater..

[260] Kim K.H., Hong S.K., Jang N.S., Ha S.H., Lee H.W., Kim J.M. (2017). Wearable resistive pressure sensor based on highly flexible carbon composite conductors with irregular surface morphology. ACS Appl. Mater. Interfaces.

[261] Ishikawa F.N., Chang H.K., Ryu K., Chen P.C., Badmaev A., Gomez De Arco L., Shen G., Zhou C. (2009). Transparent electronics based on transfer printed aligned carbon nanotubes on rigid and flexible substrates. ACS Nano.

[262] Thostenson E.T., Chou T.-W. (2002). Aligned multi-walled carbon nanotube-reinforced composites: Processing and mechanical characterization. J. Phys. D Appl. Phys..

[263] Mikhalchan A., Gspann T., Windle A. (2016). Aligned carbon nanotube–epoxy composites: The effect of nanotube organization on strength, stiffness, and toughness. J. Mater. Sci..

[264] Geim A.K. (2009). Graphene: Status and prospects. Science.

[265] Singh V., Joung D., Zhai L., Das S., Khondaker S.I., Seal S. (2011). Graphene based materials: Past, present and future. Progress Mater. Sci..

[266] Tiginyanu I., Ursaki V., Popa V., Makhlouf A.S.H., Tiginyanu I. (2011). Ultra-thin membranes for sensor applications. Nanocoatings and Ultra-Thin Films.

[267] Azizighannad S., Mitra S. (2018). Stepwise Reduction of Graphene Oxide (GO) and its effects on chemical and colloidal properties. Sci. Rep..

[268] Pyo S., Choi J., Kim J. (2018). Flexible, Transparent, Sensitive, and Crosstalk-Free Capacitive Tactile Sensor Array Based on Graphene Electrodes and Air Dielectric. Adv. Electron. Mater..

[269] Meng Y., Zhao J., Yang X., Zhao C., Qin S., Cho J.H., Zhang C., Sun Q., Wang Z.L. (2018). Mechanosensation-active matrix based on direct-contact tribotronic planar graphene transistor array. ACS Nano.

[270] Shin S.H., Ji S., Choi S., Pyo K.H., Wan An B., Park J., Kim J., Kim J.Y., Lee K.S., Kwon S.Y. (2017). Integrated arrays of air-dielectric graphene transistors as transparent active-matrix pressure sensors for wide pressure ranges. Nat. Commun..

[271] Zhu Y., Cai H., Ding H., Pan N., Wang X. (2019). Fabrication of low-cost and highly sensitive graphene-based pressure sensors by direct laser scribing polydimethylsiloxane. ACS Appl. Mater. Interfaces.

[272] Wang X., Gu Y., Xiong Z., Cui Z., Zhang T. (2014). Silk-molded flexible, ultrasensitive, and highly stable electronic skin for monitoring human physiological signals. Adv. Mater..

[273] Yao H.B., Ge J., Wang C.F., Wang X., Hu W., Zheng Z.J., Ni Y., Yu S.H. (2013). A flexible and highly pressure-sensitive graphene-polyurethane sponge based on fractured microstructure design. Adv. Mater..

[274] Zhu S.-E., Krishna Ghatkesar M., Zhang C., Janssen G.C.A.M. (2013). Graphene based piezoresistive pressure sensor. Appl. Phys. Lett..

[275] Tao L.Q., Zhang K.N., Tian H., Liu Y., Wang D.Y., Chen Y.Q., Yang Y., Ren T.L. (2017). Graphene-Paper pressure sensor for detecting human motions. ACS Nano.

[276] Zhu L., Wang Y., Mei D., Wu X. (2019). Highly sensitive and flexible tactile sensor based on porous graphene sponges for distributed tactile sensing in monitoring human motions. J. Microelectromech. Syst..

[277] Berger C., Phillips R., Centeno A., Zurutuza A., Vijayaraghavan A. (2017). Capacitive pressure sensing with suspended graphene-polymer heterostructure membranes. Nanoscale.

[278] Khan U., Kim T.H., Ryu H., Seung W., Kim S.W. (2017). Graphene tribotronics for electronic skin and touch screen applications. Adv. Mater..

[279] Ge G., Cai Y., Dong Q., Zhang Y., Shao J., Huang W., Dong X. (2018). A flexible pressure sensor based on rGO/polyaniline wrapped sponge with tunable sensitivity for human motion detection. Nanoscale.

[280] Liu W., Zhang X., Wei G., Su Z. (2018). Reduced graphene oxide-based double network polymeric hydrogels for pressure and temperature sensing. Sensors.

[281] Zhang B.-X., Hou Z.-L., Yan W., Zhao Q.-L., Zhan K.-T. (2017). Multi-dimensional flexible reduced graphene oxide/polymer sponges for multiple forms of strain sensors. Carbon.

[282] Jia J., Huang G., Deng J., Pan K. (2019). Skin-inspired flexible and high-sensitivity pressure sensors based on rGO films with continuous-gradient wrinkles. Nanoscale.

[283] Ai Y., Hsu T.H., Wu D.C., Lee L., Chen J.-H., Chen Y.-Z., Wu S.-C., Wu C., Wang Z.M., Chueh Y.-L. (2018). An ultrasensitive flexible pressure sensor for multimodal wearable electronic skins based on large-scale polystyrene ball@reduced graphene-oxide core–shell nanoparticles. J. Mater. Chem. C.

[284] Kim S.J., Mondal S., Min B.K., Choi C.G. (2018). Highly sensitive and flexible strain-pressure sensors with cracked paddy-shaped mos2/graphene foam/ecoflex hybrid nanostructures. ACS Appl. Mater. Interfaces.

[285] Liu W., Liu N., Yue Y., Rao J., Cheng F., Su J., Liu Z., Gao Y. (2018). Piezoresistive pressure sensor based on synergistical innerconnect polyvinyl alcohol nanowires/wrinkled graphene film. Small.

[286] Kweon O.Y., Lee S.J., Oh J.H. (2018). Wearable high-performance pressure sensors based on three-dimensional electrospun conductive nanofibers. NPG Asia Mater..

[287] Persano L., Dagdeviren C., Su Y., Zhang Y., Girardo S., Pisignano D., Huang Y., Rogers J.A. (2013). High performance piezoelectric devices based on aligned arrays of nanofibers of poly(vinylidenefluoride-co-trifluoroethylene). Nat. Commun..

[288] Zhong W., Liu Q., Wu Y., Wang Y., Qing X., Li M., Liu K., Wang W., Wang D. (2016). A nanofiber based artificial electronic skin with high pressure sensitivity and 3D conformability. Nanoscale.

[289] Gong S., Schwalb W., Wang Y., Chen Y., Tang Y., Si J., Shirinzadeh B., Cheng W. (2014). A wearable and highly sensitive pressure sensor with ultrathin gold nanowires. Nat. Commun..

[290] Li S.X., Xia H., Xu Y.S., Lv C., Wang G., Dai Y.Z., Sun H.B. (2019). Gold nanoparticle densely packed micro/nanowire-based pressure sensors for human motion monitoring and physiological signal detection. Nanoscale.

[291] Wang Y., Chao M., Wan P., Zhang L. (2020). A wearable breathable pressure sensor from metal-organic framework derived nanocomposites for highly sensitive broad-range healthcare monitoring. Nano Energy.

[292] Zhao X.H., Ma S.N., Long H., Yuan H., Tang C.Y., Cheng P.K., Tsang Y.H. (2018). Multifunctional sensor based on porous carbon derived from metal-organic frameworks for real time health monitoring. ACS Appl. Mater. Interfaces.

[293] Pang C., Lee G.Y., Kim T.I., Kim S.M., Kim H.N., Ahn S.H., Suh K.Y. (2012). A flexible and highly sensitive strain-gauge sensor using reversible interlocking of nanofibres. Nat. Mater..

[294] Zhuo B., Chen S., Zhao M., Guo X. (2017). High sensitivity flexible capacitive pressure sensor using polydimethylsiloxane elastomer dielectric layer micro-structured by 3-D printed mold. IEEE J. Electron. Dev. Soc..

[295] Wu N., Chen S., Lin S., Li W., Xu Z., Yuan F., Huang L., Hu B., Zhou J. (2018). Theoretical study and structural optimization of a flexible piezoelectret-based pressure sensor. J. Mater. Chem. A.

[296] Mannsfeld S.C., Tee B.C., Stoltenberg R.M., Chen C.V., Barman S., Muir B.V., Sokolov A.N., Reese C., Bao Z. (2010). Highly sensitive flexible pressure sensors with microstructured rubber dielectric layers. Nat. Mater..

[297] Shuai X., Zhu P., Zeng W., Hu Y., Liang X., Zhang Y., Sun R., Wong C.P. (2017). Highly sensitive flexible pressure sensor based on silver nanowires-embedded polydimethylsiloxane electrode with microarray structure. ACS Appl. Mater. Interfaces.

[298] Lin J., Peng Z., Liu Y., Ruiz-Zepeda F., Ye R., Samuel E.L., Yacaman M.J., Yakobson B.I., Tour J.M. (2014). Laser-induced porous graphene films from commercial polymers. Nat. Commun..

[299] Eom J., Jaisutti R., Lee H., Lee W., Heo J.S., Lee J.Y., Park S.K., Kim Y.H. (2017). Highly sensitive textile strain sensors and wireless user-interface devices using all-polymeric conducting fibers. ACS Appl. Mater. Interfaces.

[300] Nandi A.K., Mandelkern L. (1991). The influence of chain structure on the equilibrium melting temperature of poly(vinylidene fluoride). J. Polym. Sci. Part B Polym. Phys..

[301] Salimi A., Yousefi A.A. (2003). Analysis Method: FTIR studies of β-phase crystal formation in stretched PVDF films. Polym. Test..

[302] Giannetti E. (2001). Semi-crystalline fluorinated polymers. Polym. Int..

[303] Wang M., Gurunathan R., Imasato K., Geisendorfer N.R., Jakus A.E., Peng J., Shah R.N., Grayson M., Snyder G.J. (2018). A Percolation Model for Piezoresistivity in Conductor–Polymer Composites. Adv. Theory Simul..

[304] Leleux P., Badier J.M., Rivnay J., Benar C., Herve T., Chauvel P., Malliaras G.G. (2014). Conducting polymer electrodes for electroencephalography. Adv. Healthcare Mater..

[305] Khodashenas B., Ghorbani H.R. (2019). Synthesis of silver nanoparticles with different shapes. Arab. J. Chem..

[306] Zhang Y., Wu J., Aagesen M., Liu H. (2015). III–V nanowires and nanowire optoelectronic devices. J. Phys. D Appl. Phys..

[307] Signorello G., Sant S., Bologna N., Schraff M., Drechsler U., Schmid H., Wirths S., Rossell M.D., Schenk A., Riel H. (2017). manipulating surface states of iii-v nanowires with uniaxial stress. Nano Lett..

[308] Lee P., Lee J., Lee H., Yeo J., Hong S., Nam K.H., Lee D., Lee S.S., Ko S.H. (2012). Highly stretchable and highly conductive metal electrode by very long metal nanowire percolation network. Adv. Mater..

[309] Wang L., Chen Q.D., Cao X.W., Buividas R., Wang X., Juodkazis S., Sun H.B. (2017). Plasmonic nano-printing: Large-area nanoscale energy deposition for efficient surface texturing. Light Sci. Appl..

[310] Pan L., Liu G., Shi W., Shang J., Leow W.R., Liu Y., Jiang Y., Li S., Chen X., Li R.W. (2018). Mechano-regulated metal-organic framework nanofilm for ultrasensitive and anti-jamming strain sensing. Nat. Commun..

[311] Li H., Eddaoudi M., O’Keeffe M., Yaghi O.M. (1999). Design and synthesis of an exceptionally stable and highly porous metal-organic framework. Nature.

[312] Zhou H.C., Long J.R., Yaghi O.M. (2012). Introduction to metal-organic frameworks. Chem. Rev..

[313] Pathak A., Shen J.W., Usman M., Wei L.F., Mendiratta S., Chang Y.S., Sainbileg B., Ngue C.M., Chen R.S., Hayashi M. (2019). Integration of a (-Cu-S-)n plane in a metal-organic framework affords high electrical conductivity. Nat. Commun..

[314] Le Ouay B., Boudot M., Kitao T., Yanagida T., Kitagawa S., Uemura T. (2016). Nanostructuration of PEDOT in porous coordination polymers for tunable porosity and conductivity. J. Am. Chem. Soc..

[315] Kousik S., Velmathi S. (2019). Engineering Metal-organic framework catalysts for C-C and C-X coupling reactions: Advances in reticular approaches from 2014-2018. Chemistry.

[316] Zhang S., Pei X., Gao H., Chen S., Wang J. (2019). Metal-organic framework-based nanomaterials for biomedical applications. Chin. Chem. Lett..

[317] Hantanasirisakul K., Gogotsi Y. (2018). Electronic and optical properties of 2D transition metal carbides and nitrides (MXenes). Adv. Mater..

[318] Luo J.-Q., Zhao S., Zhang H.-B., Deng Z., Li L., Yu Z.-Z. (2019). Flexible, stretchable and electrically conductive MXene/natural rubber nanocomposite films for efficient electromagnetic interference shielding. Compos. Sci. Technol..

[319] Anasori B., Lukatskaya M.R., Gogotsi Y. (2017). 2D metal carbides and nitrides (MXenes) for energy storage. Nat. Rev. Mater..

[320] Lipatov A., Lu H., Alhabeb M., Anasori B., Gruverman A., Gogotsi Y., Sinitskii A. (2018). Elastic properties of 2D Ti3C2T x MXene monolayers and bilayers. Sci. Adv..

[321] Liu H., Zhao G., Wu M., Liu Z., Xiang D., Wu C., Cheng Y., Wang H., Wang Z.L., Li L. (2019). Ionogel infiltrated paper as flexible electrode for wearable all-paper based sensors in active and passive modes. Nano Energy.

[322] Kim K., Jung M., Kim B., Kim J., Shin K., Kwon O.-S., Jeon S. (2017). Low-voltage, high-sensitivity and high-reliability bimodal sensor array with fully inkjet-printed flexible conducting electrode for low power consumption electronic skin. Nano Energy.

[323] Fan X., Xu B., Wang N., Wang J., Liu S., Wang H., Yan F. (2017). Highly conductive stretchable all-plastic electrodes using a novel dipping-embedded transfer method for high-performance wearable sensors and semitransparent organic solar cells. Adv. Electron. Mater..

[324] Kim K., Song G., Park C., Yun K.S. (2017). Multifunctional woven structure operating as triboelectric energy harvester, capacitive tactile sensor array, and piezoresistive strain sensor array. Sensors.

[325] Ding S., Jiu J., Gao Y., Tian Y., Araki T., Sugahara T., Nagao S., Nogi M., Koga H., Suganuma K. (2016). One-Step Fabrication of Stretchable Copper Nanowire Conductors by a Fast Photonic Sintering Technique and Its Application in Wearable Devices. ACS Appl. Mater. Interfaces.

[326] Choo D.C., Bae S.K., Kim T.W. (2019). Flexible, transparent patterned electrodes based on graphene oxide/silver nanowire nanocomposites fabricated utilizing an accelerated ultraviolet/ozone process to control silver nanowire degradation. Sci. Rep..

[327] Hyun W.J., Secor E.B., Hersam M.C., Frisbie C.D., Francis L.F. (2015). High-resolution patterning of graphene by screen printing with a silicon stencil for highly flexible printed electronics. Adv. Mater..

[328] Zhang Y., Zhang L., Cui K., Ge S., Cheng X., Yan M., Yu J., Liu H. (2018). Flexible electronics based on micro/nanostructured paper. Adv. Mater..

[329] Xu B., Gopalan S.A., Gopalan A.I., Muthuchamy N., Lee K.P., Lee J.S., Jiang Y., Lee S.W., Kim S.W., Kim J.S. (2017). Functional solid additive modified PEDOT:PSS as an anode buffer layer for enhanced photovoltaic performance and stability in polymer solar cells. Sci. Rep..

[330] Pereni C.I., Zhao Q., Liu Y., Abel E. (2006). Surface free energy effect on bacterial retention. Colloids Surf. B Biointerfaces.

[331] Gusnaniar N., van der Mei H.C., Qu W., Nuryastuti T., Hooymans J.M.M., Sjollema J., Busscher H.J. (2017). Physico-chemistry of bacterial transmission versus adhesion. Adv. Colloid Interface Sci..

[332] Oh J.Y., Rondeau-Gagne S., Chiu Y.C., Chortos A., Lissel F., Wang G.N., Schroeder B.C., Kurosawa T., Lopez J., Katsumata T. (2016). Intrinsically stretchable and healable semiconducting polymer for organic transistors. Nature.

[333] Lee Y., Park J., Choe A., Cho S., Kim J., Ko H. (2019). Mimicking human and biological skins for multifunctional skin electronics. Adv. Funct. Mater..

[334] Shimizu R., Nonomura Y. (2018). Preparation of artificial skin that mimics human skin surface and mechanical properties. J. Oleo Sci..

[335] Wang T., Si Y., Luo S., Dong Z., Jiang L. (2019). Wettability manipulation of overflow behavior via vesicle surfactant for water-proof surface cleaning. Mater. Horiz..

[336] Stadlober B., Zirkl M., Irimia-Vladu M. (2019). Route towards sustainable smart sensors: Ferroelectric polyvinylidene fluoride-based materials and their integration in flexible electronics. Chem. Soc. Rev..

[337] Santarelli L. (2018). Organic Semiconductors-Based Devices Electrical Reliability to Environmental Stress. Ph.D. Thesis.

[338] Xu K., Lu Y., Takei K. (2019). Multifunctional skin-inspired flexible sensor systems for wearable electronics. Adv. Mater. Technol..

[339] Kenry, Yeo J.C., Lim C.T. (2016). Emerging flexible and wearable physical sensing platforms for healthcare and biomedical applications. Microsyst. Nanoeng..

[340] Song F., Ren D. (2014). Stiffness of cross-linked poly(dimethylsiloxane) affects bacterial adhesion and antibiotic susceptibility of attached cells. Langmuir.

[341] Song F., Brasch M.E., Wang H., Henderson J.H., Sauer K., Ren D. (2017). How bacteria respond to material stiffness during attachment: A role of escherichia coli flagellar motility. ACS Appl. Mater. Interfaces.

[342] Song F., Wang H., Sauer K., Ren D. (2018). Cyclic-di-GMP and oprF are involved in the response of pseudomonas aeruginosa to substrate material stiffness during attachment on polydimethylsiloxane (PDMS). Front. Microbiol..

[343] Hia I.L., Vahedi V., Pasbakhsh P. (2016). Self-healing polymer composites: Prospects, challenges, and applications. Polym. Rev..

[344] Latif S., Amin S., Haroon S.S., Sajjad I.A. (2019). Self-healing materials for electronic applications: An overview. Mater. Res. Express.

[345] Toohey K.S., Sottos N.R., Lewis J.A., Moore J.S., White S.R. (2007). Self-healing materials with microvascular networks. Nat. Mater..

[346] Bartlett M.D., Dickey M.D., Majidi C. (2019). Self-healing materials for soft-matter machines and electronics. NPG Asia Mater..

[347] Wang Y., Adokoh C.K., Narain R. (2018). Recent development and biomedical applications of self-healing hydrogels. Exp. Opin. Drug Deliv..

[348] Patrick J.F., Robb M.J., Sottos N.R., Moore J.S., White S.R. (2016). Polymers with autonomous life-cycle control. Nature.

[349] Fritzen C.P., Kraemer P. (2009). Self-diagnosis of smart structures based on dynamical properties. Mech. Syst. Sign. Process..

[350] Choi S.-B. (2014). The grand challenges in smart materials research. Front. Mater..

[351] Song P., Yang G., Lang T., Yong K.-T. (2019). Nanogenerators for wearable bioelectronics and biodevices. J. Phys. D Appl. Phys..

[352] Costerton J.W., Stewart P.S., Greenberg E.P. (1999). Bacterial biofilms: A common cause of persistent infections. Science.

[353] Liaqat I., Gotsiridze-Columbus N., Bailey W.C. (2011). Biofilms: Formation, Development and Properties. Biofilms: Formation, Development and Properties.

[354] O’Toole G., Kaplan H.B., Kolter R. (2000). Biofilm formation as microbial development. Annu. Rev. Microbiol..

[355] Obolski U., Stein G.Y., Hadany L. (2015). antibiotic restriction might facilitate the emergence of multi-drug resistance. PLoS Comput. Biol..

[356] MacCallum N., Howell C., Kim P., Sun D., Friedlander R., Ranisau J., Ahanotu O., Lin J.J., Vena A., Hatton B. (2014). Liquid-infused silicone as a biofouling-free medical material. ACS Biomater. Sci. Eng..

[357] Miller-Petrie M., Pant S., Laxminarayan R., Holmes K.K., Bertozzi S., Bloom B.R., Jha P. (2017). Drug-Resistant Infections. Major Infectious Diseases.

[358] Barthlott W., Neinhuis C. (1997). Purity of the sacred lotus, or escape from contamination in biological surfaces. Planta.

[359] Kargar M., Chang Y.-R., Khalili Hoseinabad H., Pruden A., Ducker W.A. (2016). colloidal crystals delay formation of early stage bacterial biofilms. ACS Biomater. Sci. Eng..

[360] Fadeeva E., Truong V.K., Stiesch M., Chichkov B.N., Crawford R.J., Wang J., Ivanova E.P. (2011). Bacterial retention on superhydrophobic titanium surfaces fabricated by femtosecond laser ablation. Langmuir.

[361] Lei W., Bruchmann J., Ruping J.L., Levkin P.A., Schwartz T. (2019). Biofilm bridges forming structural networks on patterned lubricant-infused surfaces. Adv. Sci. (Weinh).

[362] Moazzam P., Razmjou A., Golabi M., Shokri D., Landarani-Isfahani A. (2016). Investigating the BSA protein adsorption and bacterial adhesion of Al-alloy surfaces after creating a hierarchical (micro/nano) superhydrophobic structure. J. Biomed. Mater. Res. A.

[363] Achinas S., Charalampogiannis N., Euverink G.J.W. (2019). A brief recap of microbial adhesion and biofilms. Appl. Sci..

[364] Carniello V., Peterson B.W., van der Mei H.C., Busscher H.J. (2018). Physico-chemistry from initial bacterial adhesion to surface-programmed biofilm growth. Adv. Colloid Interface Sci..

[365] Becker K. (1998). Detachment studies on microfouling in natural biofilms on substrata with different surface tensions. Int. Biodet. Biodegrad..

[366] Tsibouklis J., Stone M., Thorpe A.A., Graham P., Peters V., Heerlien R., Smith J.R., Green K.L., Nevell T.G. (1999). Preventing bacterial adhesion onto surfaces: The low-surface-energy approach. Biomaterials.

[367] Reche I., D’Orta G., Mladenov N., Winget D.M., Suttle C.A. (2018). Deposition rates of viruses and bacteria above the atmospheric boundary layer. ISME J..

[368] Yang W., Elankumaran S., Marr L.C. (2011). Concentrations and size distributions of airborne influenza A viruses measured indoors at a health centre, a day-care centre and on aeroplanes. J. R. Soc. Interface.

[369] Sjollema J., van der Mei H.C., Hall C.L., Peterson B.W., de Vries J., Song L., Jong E.D., Busscher H.J., Swartjes J. (2017). Detachment and successive re-attachment of multiple, reversibly-binding tethers result in irreversible bacterial adhesion to surfaces. Sci. Rep..

[370] Poortinga A.T., Bos R., Norde W., Busscher H.J. (2002). Electric double layer interactions in bacterial adhesion to surfaces. Surf. Sci. Rep..

[371] Hong Y., Brown D.G. (2008). Electrostatic behavior of the charge-regulated bacterial cell surface. Langmuir.

[372] Soni K.A., Balasubramanian A.K., Beskok A., Pillai S.D. (2008). Zeta potential of selected bacteria in drinking water when dead, starved, or exposed to minimal and rich culture media. Curr. Microbiol..

[373] Samandoulgou I., Fliss I., Jean J. (2015). Zeta Potential and aggregation of virus-like particle of human norovirus and feline calicivirus under different physicochemical conditions. Food Environ. Virol..

[374] Tan M.S., White A.P., Rahman S., Dykes G.A. (2016). Role of fimbriae, flagella and cellulose on the attachment of salmonella typhimurium ATCC 14028 to plant cell wall models. PLoS ONE.

[375] Hochbaum A.I., Aizenberg J. (2010). Bacteria pattern spontaneously on periodic nanostructure arrays. Nano Lett..

[376] Hsu L.C., Fang J., Borca-Tasciuc D.A., Worobo R.W., Moraru C.I. (2013). Effect of micro- and nanoscale topography on the adhesion of bacterial cells to solid surfaces. Appl. Environ. Microbiol..

[377] Spengler C., Nolle F., Mischo J., Faidt T., Grandthyll S., Thewes N., Koch M., Muller F., Bischoff M., Klatt M.A. (2019). Strength of bacterial adhesion on nanostructured surfaces quantified by substrate morphometry. Nanoscale.

[378] Hori K., Matsumoto S. (2010). Bacterial adhesion: From mechanism to control. Biochem. Eng. J..

[379] Maattanen A., Fallarero A., Kujala J., Ihalainen P., Vuorela P., Peltonen J. (2014). Printed paper-based arrays as substrates for biofilm formation. AMB Express.

[380] Cheng Y., Feng G., Moraru C.I. (2019). Micro- and Nanotopography sensitive bacterial attachment mechanisms: A review. Front. Microbiol..

[381] Gristina A.G. (1987). Biomaterial-centered infection: Microbial adhesion versus tissue integration. Science.

[382] Bos R., van der Mei H.C., Busscher H.J. (1999). Physico-chemistry of initial microbial adhesive interactions-its mechanisms and methods for study. FEMS Microbiol. Rev..

[383] Van der Mei H.C., Bos R., Busscher H.J. (1998). A reference guide to microbial cell surface hydrophobicity based on contact angles. Colloids Surf. B Biointerfaces.

[384] Zhang X., Zhang Q., Yan T., Jiang Z., Zhang X., Zuo Y.Y. (2015). Quantitatively predicting bacterial adhesion using surface free energy determined with a spectrophotometric method. Environ. Sci. Technol..

[385] Swartjes J.J.T.M., Veeregowda D.H., van der Mei H.C., Busscher H.J., Sharma P.K. (2014). Normally oriented adhesion versus friction forces in bacterial adhesion to polymer-brush functionalized surfaces under fluid flow. Adv. Funct. Mater..

[386] Valentin J.D.P., Qin X.H., Fessele C., Straub H., van der Mei H.C., Buhmann M.T., Maniura-Weber K., Ren Q. (2019). Substrate viscosity plays an important role in bacterial adhesion under fluid flow. J. Colloid Interface Sci..

[387] Freschauf L.R., McLane J., Sharma H., Khine M. (2012). Shrink-induced superhydrophobic and antibacterial surfaces in consumer plastics. PLoS ONE.

[388] Bonner M.C., Tunney M.M., Jones D.S., Gorman S.P. (1997). Factors affecting in vitro adherence of ureteral stent biofilm isolates to polyurethane. Int. J. Pharm..

[389] Reid G., Beg H.S., Preston C.A.K., Hawthorn L.A. (1991). Effect of bacterial, urine and substratum surface tension properties on bacterial adhesion to biomaterials. Biofouling.

[390] Dou X.Q., Zhang D., Feng C., Jiang L. (2015). Bioinspired hierarchical surface structures with tunable wettability for regulating bacteria adhesion. ACS Nano.

[391] Chung K.K., Schumacher J.F., Sampson E.M., Burne R.A., Antonelli P.J., Brennan A.B. (2007). Impact of engineered surface microtopography on biofilm formation of Staphylococcus aureus. Biointerphases.

[392] Dundar Arisoy F., Kolewe K.W., Homyak B., Kurtz I.S., Schiffman J.D., Watkins J.J. (2018). Bioinspired Photocatalytic shark-skin surfaces with antibacterial and antifouling activity via nanoimprint lithography. ACS Appl. Mater. Interfaces.

[393] Yuan Y., Hays M.P., Hardwidge P.R., Kim J. (2017). Surface characteristics influencing bacterial adhesion to polymeric substrates. RSC Adv..

[394] Pillet F., Formosa-Dague C., Baaziz H., Dague E., Rols M.P. (2016). Cell wall as a target for bacteria inactivation by pulsed electric fields. Sci. Rep..

[395] Zhu A., Liu H.K., Long F., Su E., Klibanov A.M. (2015). Inactivation of bacteria by electric current in the presence of carbon nanotubes embedded within a polymeric membrane. Appl. Biochem. Biotechnol..

[396] Montazerian H., Rashidi A., Dalili A., Najjaran H., Milani A.S., Hoorfar M. (2019). Graphene-coated spandex sensors embedded into silicone sheath for composites health monitoring and wearable applications. Small.

[397] Yu L., Chen G.Y., Xu H., Liu X. (2016). Substrate-Independent, Transparent oil-repellent coatings with self-healing and persistent easy-sliding oil repellency. ACS Nano.

[398] Prosheva M., Aboudzadeh M.A., Leal G.P., Gilev J.B., Tomovska R. (2019). High-performance uv protective waterborne polymer coatings based on hybrid graphene/carbon nanotube radicals scavenging filler. Part. Part. Syst. Charact..

[399] Nuraje N., Khan S.I., Misak H., Asmatulu R. (2013). The Addition of graphene to polymer coatings for improved weathering. ISRN Polym. Sci..

[400] Cui X., Zhu G., Pan Y., Shao Q., Zhao C., Dong M., Zhang Y., Guo Z. (2018). Polydimethylsiloxane-titania nanocomposite coating: Fabrication and corrosion resistance. Polymer.

[401] Nakamura A., Hamanishi T., Kawakami S., Takeda M. (2017). A piezo-resistive graphene strain sensor with a hollow cylindrical geometry. Mater. Sci. Eng. B.

[402] Maya-Cornejo J., Rodríguez-Gómez F.J., Molina G.A., Galindo-de-la-Rosa J., Ledesma-García J., Hernández-Martínez Á.R., Esparza R., Pérez R., Estévez M. (2019). Electrochemical study of a hybrid polymethyl methacrylate coating using SIO2 nanoparticles toward the mitigation of the corrosion in marine environments. Materials.

[403] Radwan A.B., Mohamed A.M.A., Abdullah A.M., Al-Maadeed M.A. (2016). Corrosion protection of electrospun PVDF–ZnO superhydrophobic coating. Surf. Coatings Technol..

[404] Xiao Y.-K., Ji W.-F., Chang K.-S., Hsu K.-T., Yeh J.-M., Liu W.-R. (2017). Sandwich-structured rGO/PVDF/PU multilayer coatings for anti-corrosion application. RSC Adv..

[405] Gao H., Minh P.T., Wang H., Minko S., Locklin J., Nguyen T., Sharma S. (2018). High-performance flexible yarn for wearable piezoelectric nanogenerators. Smart Mater. Struct..

[406] Satyanarayana N., Minn M., Samad M.A., Sinha S.K., Wang Q.J., Chung Y.-W. (2013). Polymer Tribological Coatings. Encyclopedia of Tribology.

[407] Cui G., Bi Z., Zhang R., Liu J., Yu X., Li Z. (2019). A comprehensive review on graphene-based anti-corrosive coatings. Chem. Eng. J..

[408] Li J., Wang L., Wang X., Yang Y., Hu Z., Liu L., Huang Y. (2020). Highly Conductive PVA/Ag Coating by Aqueous in Situ Reduction and Its Stretchable Structure for Strain Sensor. ACS Appl. Mater. Interfaces.

[409] Busuioc C., Evanghelidis A., Galatanu A., Enculescu I. (2016). Direct and contactless electrical control of temperature of paper and textile foldable substrates using electrospun metallic-web transparent electrodes. Sci. Rep..

[410] Chun S., Choi Y., Park W. (2017). All-graphene strain sensor on soft substrate. Carbon.

[411] Li X., Koh K.H., Farhan M., Lai K.W.C. (2020). An ultraflexible polyurethane yarn-based wearable strain sensor with a polydimethylsiloxane infiltrated multilayer sheath for smart textiles. Nanoscale.

[412] Health D., Therapeutic Goods Administration (2016). Literature Review on the Safety Of Titanium Dioxide and Zinc Oxide Nanoparticles in Sunscreens.

[413] Dong C., Fu Y., Zang W., He H., Xing L., Xue X. (2017). Self-powering/self-cleaning electronic-skin basing on PVDF/TiO2 nanofibers for actively detecting body motion and degrading organic pollutants. Appl. Surf. Sci..

[414] Feng Y., Li H., Ling L., Yan S., Pan D., Ge H., Li H., Bian Z. (2018). Enhanced photocatalytic degradation performance by fluid-induced piezoelectric field. Environ. Sci. Technol..

[415] Su C., Tong Y., Zhang M., Zhang Y., Shao C. (2013). TiO2 nanoparticles immobilized on polyacrylonitrile nanofibers mats: A flexible and recyclable photocatalyst for phenol degradation. RSC Adv..

[416] Marelli B., Brenckle M.A., Kaplan D.L., Omenetto F.G. (2016). Silk fibroin as edible coating for perishable food preservation. Sci. Rep..

[417] Umuhoza D., Yang F., Long D., Hao Z., Dai J., Zhao A. (2020). Strategies for tuning the biodegradation of silk fibroin-based materials for tissue engineering applications. ACS Biomater. Sci. Eng..

[418] Ling S., Qin Z., Li C., Huang W., Kaplan D.L., Buehler M.J. (2017). Polymorphic regenerated silk fibers assembled through bioinspired spinning. Nat. Commun..

[419] Wang C., Li X., Gao E., Jian M., Xia K., Wang Q., Xu Z., Ren T., Zhang Y. (2016). Carbonized silk fabric for ultrastretchable, highly sensitive, and wearable strain sensors. Adv. Mater..

[420] Li L., Bai Y., Li L., Wang S., Zhang T. (2017). A Superhydrophobic smart coating for flexible and wearable sensing electronics. Adv. Mater..

[421] Fu Q., Rao G.V.R., Ista L.K., Wu Y., Andrzejewski B.P., Sklar L.A., Ward T.L., López G.P. (2003). Control of molecular transport through stimuli-responsive ordered mesoporous materials. Adv. Mater..

[422] Stumpel J.E., Broer D.J., Schenning A.P. (2014). Stimuli-responsive photonic polymer coatings. Chem. Commun. (Cambridge).

[423] Cotting F., Koebsch A., Aoki I.V. (2019). Epoxy self-healing coating by encapsulated epoxy ester resin in poly (urea-formaldehyde-melamine) microcapsules. Front. Mater..

[424] Lazauskas A., Jucius D., Baltrusaitis V., Gudaitis R., Prosycevas I., Abakeviciene B., Guobiene A., Andrulevicius M., Grigaliunas V. (2019). Shape-memory assisted scratch-healing of transparent thiol-ene coatings. Materials.

[425] Coppola A.M., Griffin A.S., Sottos N.R., White S.R. (2015). Retention of mechanical performance of polymer matrix composites above the glass transition temperature by vascular cooling. Compos. Part A Appl. Sci. Manufact..

[426] Xu K., Lu Y., Yamaguchi T., Arie T., Akita S., Takei K. (2019). Highly precise multifunctional thermal management-based flexible sensing sheets. ACS Nano.

[427] Xu D., Zhang Y., Wang B., Yang H., Ban J., Liu F., Li T. (2019). Acute effects of temperature exposure on blood pressure: An hourly level panel study. Environ. Int..

[428] Halonen J.I., Zanobetti A., Sparrow D., Vokonas P.S., Schwartz J. (2011). Relationship between outdoor temperature and blood pressure. Occup. Environ. Med..

[429] Cheng W., Wang J., Ma Z., Yan K., Wang Y., Wang H., Li S., Li Y., Pan L., Shi Y. (2018). Flexible pressure sensor with high sensitivity and low hysteresis based on a hierarchically microstructured electrode. IEEE Electron. Dev. Lett..

[430] Lu Y., Sathasivam S., Song J., Crick C.R., Carmalt C.J., Parkin I.P. (2015). Repellent materials. Robust self-cleaning surfaces that function when exposed to either air or oil. Science.

[431] Fernandez-Blazquez J.P., Fell D., Bonaccurso E., del Campo A. (2011). Superhydrophilic and superhydrophobic nanostructured surfaces via plasma treatment. J. Colloid Interface Sci..

[432] Pan S., Kota A.K., Mabry J.M., Tuteja A. (2013). Superomniphobic surfaces for effective chemical shielding. J. Am. Chem. Soc..

[433] Zhang P., Lin L., Zang D., Guo X., Liu M. (2017). Designing bioinspired anti-biofouling surfaces based on a superwettability strategy. Small.

[434] Hermelin E., Petitjean J., Lacroix J.-C., Chane-Ching K.I., Tanguy J., Lacaze P.-C. (2008). Ultrafast electrosynthesis of high hydrophobic polypyrrole coatings on a zinc electrode: Applications to the protection against corrosion. Chem. Mater..

[435] (2019). Bi; Li; Zhao; Ran; Cao; Guo; Xue, Robust super-hydrophobic coating prepared by electrochemical surface engineering for corrosion protection. Coatings.

[436] Wang P., Sun B., Liang Y., Han H., Fan X., Wang W., Yang Z. (2018). A stretchable and super-robust graphene superhydrophobic composite for electromechanical sensor application. J. Mater. Chem. A.

[437] Rawal S.P., Goodman J.W., Kelly A., Zweben C. (2000). Composites for Spacecraft. Comprehensive Composite Materials.

[438] Ali N., Hong J.-E. (2018). Failure detection and prevention for cyber-physical systems using ontology-based knowledge base. Computers.

[439] Rodgers M.A.J. (2002). Chromic Phenomena: Technological Applications of Color Chemistry. J. Am. Chem. Soc..

[440] Pucci A. (2019). Mechanochromic Fluorescent polymers with aggregation-induced emission features. Sensors.

[441] Fu F., Hu L., Fan M., Fu F. (2017). Temperature sensitive colour-changed composites. Advanced High Strength Natural Fibre Composites in Construction.

[442] Kong L.B., Ruan S., Xiao Z., Li X., Zhou K., Su H., Wang C., Zhang T., Orlandi M.O. (2020). Tin oxide-based electrochromics. Tin Oxide Materials.

[443] Estupiñán D., Gegenhuber T., Blinco J.P., Barner-Kowollik C., Barner L. (2017). Self-reporting fluorescent step-growth raft polymers based on nitrile imine-mediated tetrazole-ene cycloaddition chemistry. ACS Macro Lett..

[444] Kim T.A., Robb M.J., Moore J.S., White S.R., Sottos N.R. (2018). Mechanical reactivity of two different spiropyran mechanophores in polydimethylsiloxane. Macromolecules.

[445] Gossweiler G.R., Brown C.L., Hewage G.B., Sapiro-Gheiler E., Trautman W.J., Welshofer G.W., Craig S.L. (2015). Mechanochemically Active Soft Robots. ACS Appl. Mater. Interfaces.

[446] Xie M., Hisano K., Zhu M., Toyoshi T., Pan M., Okada S., Tsutsumi O., Kawamura S., Bowen C. (2019). Flexible multifunctional sensors for wearable and robotic applications. Adv. Mater. Technol..

[447] Beiermann B.A., Kramer S.L.B., Moore J.S., White S.R., Sottos N.R. (2011). Role of mechanophore orientation in mechanochemical reactions. ACS Macro Lett..

[448] Bowser B.H., Craig S.L. (2018). Empowering mechanochemistry with multi-mechanophore polymer architectures. Polym. Chem..

[449] Li J., Nagamani C., Moore J.S. (2015). Polymer mechanochemistry: From destructive to productive. Acc. Chem. Res..

[450] Kean Z.S., Niu Z., Hewage G.B., Rheingold A.L., Craig S.L. (2013). Stress-responsive polymers containing cyclobutane core mechanophores: Reactivity and mechanistic insights. J. Am. Chem. Soc..

[451] Filonenko G.A., Khusnutdinova J.R. (2017). Dynamic phosphorescent probe for facile and reversible stress sensing. Adv. Mater..

[452] Schenzel A.M., Moszner N., Barner-Kowollik C. (2017). Self-reporting dynamic covalent polycarbonate networks. Polym. Chem..

[453] Peterson G.I., Larsen M.B., Ganter M.A., Storti D.W., Boydston A.J. (2015). 3D-printed mechanochromic materials. ACS Appl. Mater. Interfaces.

[454] Zhang Y., Zhuang G., Ouyang M., Hu B., Song Q., Sun J., Zhang C., Gu C., Xu Y., Ma Y. (2013). Mechanochromic and thermochromic fluorescent properties of cyanostilbene derivatives. Dyes Pigments.

[455] Toivola R., Jang S.-H., Baker S., Jen A.K.-Y., Flinn B.D. (2018). Thermochromic polymer film sensors for detection of incipient thermal damage in carbon fiber–epoxy composites. Sensors.

[456] Chou H.H., Nguyen A., Chortos A., To J.W., Lu C., Mei J., Kurosawa T., Bae W.G., Tok J.B., Bao Z. (2015). A chameleon-inspired stretchable electronic skin with interactive colour changing controlled by tactile sensing. Nat. Commun..

[457] Chung D.D.L. (2007). Damage detection using self-sensing concepts. Proc. Inst. Mech. Eng. Part G J. Aerospace Eng..

[458] Gao L., Thostenson E.T., Zhang Z., Chou T.-W. (2009). Coupled carbon nanotube network and acoustic emission monitoring for sensing of damage development in composites. Carbon.

[459] Odom S.A., Tyler T.P., Caruso M.M., Ritchey J.A., Schulmerich M.V., Robinson S.J., Bhargava R., Sottos N.R., White S.R., Hersam M.C. (2012). Autonomic restoration of electrical conductivity using polymer-stabilized carbon nanotube and graphene microcapsules. Appl. Phys. Lett..

[460] Baltopoulos A., Polydorides N., Pambaguian L., Vavouliotis A., Kostopoulos V. (2012). Damage identification in carbon fiber reinforced polymer plates using electrical resistance tomography mapping. J. Compos. Mater..

[461] Hu M., Peil S., Xing Y., Döhler D., Caire da Silva L., Binder W.H., Kappl M., Bannwarth M.B. (2018). Monitoring crack appearance and healing in coatings with damage self-reporting nanocapsules. Mater. Horiz..

[462] Robb M.J., Li W., Gergely R.C., Matthews C.C., White S.R., Sottos N.R., Moore J.S. (2016). A Robust damage-reporting strategy for polymeric materials enabled by aggregation-induced emission. ACS Cent. Sci..

[463] Kang J., Son D., Wang G.N., Liu Y., Lopez J., Kim Y., Oh J.Y., Katsumata T., Mun J., Lee Y. (2018). Tough and water-insensitive self-healing elastomer for robust electronic skin. Adv. Mater..

[464] Hager M.D., Greil P., Leyens C., van der Zwaag S., Schubert U.S. (2010). Self-healing materials. Adv. Mater..

[465] Blaiszik B.J., Kramer S.L.B., Olugebefola S.C., Moore J.S., Sottos N.R., White S.R. (2010). Self-healing polymers and composites. Annu. Rev. Mater. Res..

[466] Yang Y., Urban M.W. (2013). Self-healing polymeric materials. Chem. Soc. Rev..

[467] Zou W., Dong J., Luo Y., Zhao Q., Xie T. (2017). Dynamic covalent polymer networks: From old chemistry to modern day innovations. Adv. Mater..

[468] Yoon J.H., Kim S.M., Eom Y., Koo J.M., Cho H.W., Lee T.J., Lee K.G., Park H.J., Kim Y.K., Yoo H.J. (2019). Extremely Fast Self-Healable Bio-Based Supramolecular Polymer for Wearable Real-Time Sweat-Monitoring Sensor. ACS Appl. Mater. Interfaces.

[469] Cao S., Tong X., Dai K., Xu Q. (2019). A super-stretchable and tough functionalized boron nitride/PEDOT:PSS/poly(N-isopropylacrylamide) hydrogel with self-healing, adhesion, conductive and photothermal activity. J. Mater. Chem. A.

[470] Park S., Shin B.G., Jang S., Chung K. (2020). Three-dimensional self-healable touch sensing artificial skin device. ACS Appl. Mater. Interfaces.

[471] Burattini S., Greenland B.W., Merino D.H., Weng W., Seppala J., Colquhoun H.M., Hayes W., Mackay M.E., Hamley I.W., Rowan S.J. (2010). A healable supramolecular polymer blend based on aromatic π−π stacking and hydrogen-bonding interactions. J. Am. Chem. Soc..

[472] Keller M.W. (2013). CHAPTER 2. Encapsulation-Based Self-Healing Polymers and Composites. Healable Polymer Systems.

[473] Tian F., Zhong Z., Pan Y. (2018). A chemo-mechanically coupled model for capsule-based self-healing polymer materials. Int. J. Damage Mech..

[474] Li P., Liu G., Liu Y., Huang J. (2020). Microvascular network optimization of self-healing materials using non-dominated sorting genetic algorithm II and experimental validation. Sci. Progr..

[475] Corten C.C., Urban M.W. (2009). Repairing polymers using oscillating magnetic field. Adv. Mater..

[476] Bandodkar A.J., Lopez C.S., Vinu Mohan A.M., Yin L., Kumar R., Wang J. (2016). All-printed magnetically self-healing electrochemical devices. Sci. Adv..

[477] Wang Y., Liu Q., Li J., Ling L., Zhang G., Sun R., Wong C.-P. (2019). UV-triggered self-healing polyurethane with enhanced stretchability and elasticity. Polymer.

[478] Fang Y., Du X., Du Z., Wang H., Cheng X. (2017). Light- and heat-triggered polyurethane based on dihydroxyl anthracene derivatives for self-healing applications. J. Mater. Chem. A.

[479] Luo H., Wang H., Zhou H., Zhou X., Hu J., Yi G., Hao Z., Lin W. (2018). Shape memory-enhanced electrical self-healing of stretchable electrodes. Appl. Sci..

[480] Michal B.T., McKenzie B.M., Felder S.E., Rowan S.J. (2015). Metallo-, thermo-, and photoresponsive shape memory and actuating liquid crystalline elastomers. Macromolecules.

[481] Liu S., Lin Y., Wei Y., Chen S., Zhu J., Liu L. (2017). A high performance self-healing strain sensor with synergetic networks of poly(ɛ-caprolactone) microspheres, graphene and silver nanowires. Compos. Sci. Technol..

[482] Ye F., Li M., Ke D., Wang L., Lu Y. (2019). Ultrafast self-healing and injectable conductive hydrogel for strain and pressure sensors. Adv. Mater. Technol..

[483] Huynh T.-P., Haick H. (2016). Self-healing, fully functional, and multiparametric flexible sensing platform. Adv. Mater..

[484] Zhang J., Wan L., Gao Y., Fang X., Lu T., Pan L., Xuan F. (2019). Highly stretchable and self-healable mxene/polyvinyl alcohol hydrogel electrode for wearable capacitive electronic skin. Adv. Electron. Mater..

[485] Kee S., Haque M.A., Corzo D., Alshareef H.N., Baran D. (2019). Self-healing and stretchable 3D-printed organic thermoelectrics. Adv. Funct. Mater..

[486] Zou Z., Zhu C., Li Y., Lei X., Zhang W., Xiao J. (2018). Rehealable, fully recyclable, and malleable electronic skin enabled by dynamic covalent thermoset nanocomposite. Sci. Adv..

[487] Patrick J.F., Hart K.R., Krull B.P., Diesendruck C.E., Moore J.S., White S.R., Sottos N.R. (2014). Continuous self-healing life cycle in vascularized structural composites. Adv. Mater..

[488] D’Elia E., Barg S., Ni N., Rocha V.G., Saiz E. (2015). Self-healing graphene-based composites with sensing capabilities. Adv. Mater..

[489] Ahmed A.S., Ramanujan R.V. (2015). Magnetic field triggered multicycle damage sensing and self healing. Sci. Rep..

[490] Zhao M., Liu A., Wu H., Wu B., Li C., Tang W. (2014). Electrochemistry-assisted microstructuring of reduced graphene oxide-based microarrays with adjustable electrical behavior. Electrochem. Commun..

[491] Haubner K., Murawski J., Olk P., Eng L.M., Ziegler C., Adolphi B., Jaehne E. (2010). The route to functional graphene oxide. Chemphyschem.

[492] Wang Q., Ling S., Liang X., Wang H., Lu H., Zhang Y. (2019). Self-healable multifunctional electronic tattoos based on silk and graphene. Adv. Funct. Mater..

[493] Zhong M., Liu Y.-T., Xie X.-M. (2015). Self-healable, super tough graphene oxide–poly(acrylic acid) nanocomposite hydrogels facilitated by dual cross-linking effects through dynamic ionic interactions. J. Mater. Chem. B.

[494] Tang Y., Guo Q., Chen Z., Zhang X., Lu C., Cao J., Zheng Z. (2019). Scalable Manufactured Self-Healing Strain Sensors Based on Ion-Intercalated Graphene Nanosheets and Interfacial Coordination. ACS Appl. Mater. Interfaces.

[495] Hernández M., Bernal M.M., Grande A.M., Zhong N., van der Zwaag S., García S.J. (2017). Effect of graphene content on the restoration of mechanical, electrical and thermal functionalities of a self-healing natural rubber. Smart Mater. Struct..

[496] Yao Z., Kane C.L., Dekker C. (2000). High-field electrical transport in single-wall carbon nanotubes. Phys. Rev. Lett..

[497] De Volder M.F., Tawfick S.H., Baughman R.H., Hart A.J. (2013). Carbon nanotubes: Present and future commercial applications. Science.

[498] Han Y., Wu X., Zhang X., Lu C. (2017). Self-healing, highly sensitive electronic sensors enabled by metal-ligand coordination and hierarchical structure design. ACS Appl. Mater. Interfaces.

[499] Wu Q., Zou S., Gosselin F.P., Therriault D., Heuzey M.-C. (2018). 3D printing of a self-healing nanocomposite for stretchable sensors. J. Mater. Chem. C.

[500] Cai G., Wang J., Qian K., Chen J., Li S., Lee P.S. (2017). Extremely stretchable strain sensors based on conductive self-healing dynamic cross-links hydrogels for human-motion detection. Adv. Sci..

[501] Liu X., Lu C., Wu X., Zhang X. (2017). Self-healing strain sensors based on nanostructured supramolecular conductive elastomers. J. Mater. Chem. A.

[502] Bailey B.M., Leterrier Y., Garcia S.J., van der Zwaag S., Michaud V. (2015). Electrically conductive self-healing polymer composite coatings. Progress Org. Coatings.

[503] Al-Mansoori T., Norambuena-Contreras J., Garcia A. (2018). Effect of capsule addition and healing temperature on the self-healing potential of asphalt mixtures. Mater. Struct..

[504] Zamal H.H., Barba D., Aissa B., Haddad E., Rosei F. (2020). Recovery of electro-mechanical properties inside self-healing composites through microencapsulation of carbon nanotubes. Sci. Rep..

[505] Li Y., Gao F., Xue Z., Luan Y., Yan X., Guo Z., Wang Z. (2018). Synergistic effect of different graphene-CNT heterostructures on mechanical and self-healing properties of thermoplastic polyurethane composites. Mater. Des..

[506] Chen Y., Lu K., Song Y., Han J., Yue Y., Biswas S.K., Wu Q., Xiao H. (2019). A Skin-inspired stretchable, self-healing and electro-conductive hydrogel with a synergistic triple network for wearable strain sensors applied in human-motion detection. Nanomaterials.

[507] Jiang D., Wang Y., Li B., Sun C., Wu Z., Yan H., Xing L., Qi S., Li Y., Liu H. (2019). Flexible sandwich structural strain sensor based on silver nanowires decorated with self-healing substrate. Macromol. Mater. Eng..

[508] Zhang Y.-Z., Lee K.H., Anjum D.H., Sougrat R., Jiang Q., Kim H., Alshareef H.N. (2018). MXenes stretch hydrogel sensor performance to new limits. Sci. Adv..

[509] The Platform for Accelerating the Circular Economy (PACE) (2019). The E-Waste Coalition A New Circular Vision for Electronics.

[510] Sartor F., Gelissen J., van Dinther R., Roovers D., Papini G.B., Coppola G. (2018). Wrist-worn optical and chest strap heart rate comparison in a heterogeneous sample of healthy individuals and in coronary artery disease patients. BMC Sports Sci. Med. Rehabil..

[511] Abt G., Bray J., Benson A.C. (2018). The validity and inter-device variability of the Apple Watch for measuring maximal heart rate. J. Sports Sci..

[512] Lee H., Chung H., Ko H., Lee J. (2018). Wearable multichannel photoplethysmography framework for heart rate monitoring during intensive exercise. IEEE Sens. J..

[513] Winston F.K., Yan A.C. (2017). Wearable health device dermatitis: A case of acrylate-related contact allergy. Cutis.

[514] Ilankoon I., Ghorbani Y., Chong M.N., Herath G., Moyo T., Petersen J. (2018). E-waste in the international context - A review of trade flows, regulations, hazards, waste management strategies and technologies for value recovery. Waste Manag..

[515] Fries R. (2009). Biomaterials-The Intersection of Biology and Materials Science. (Temenoff, J.S. et al.; 2008) [Book reviews]. IEEE Eng. Med. Biol. Mag..

[516] Feig V.R., Tran H., Bao Z. (2018). biodegradable polymeric materials in degradable electronic devices. ACS Cent. Sci..

[517] Williams D.F. (2008). On the mechanisms of biocompatibility. Biomaterials.

[518] Yu X., Shou W., Mahajan B.K., Huang X., Pan H. (2018). Materials, processes, and facile manufacturing for bioresorbable electronics: A review. Adv. Mater..

[519] Boutry C.M., Kaizawa Y., Schroeder B.C., Chortos A., Legrand A., Wang Z., Chang J., Fox P., Bao Z. (2018). A stretchable and biodegradable strain and pressure sensor for orthopaedic application. Nat. Electron..

[520] Salvatore G.A., Sülzle J., Dalla Valle F., Cantarella G., Robotti F., Jokic P., Knobelspies S., Daus A., Büthe L., Petti L. (2017). Biodegradable and highly deformable temperature sensors for the internet of things. Adv. Funct. Mater..

[521] Yoshioka Y., Kuroda E., Hirai T., Tsutsumi Y., Ishii K.J. (2017). Allergic Responses Induced by the Immunomodulatory Effects of Nanomaterials upon Skin Exposure. Front. Immunol..

[522] Tabei Y., Sonoda A., Nakajima Y., Biju V., Makita Y., Yoshida Y., Horie M. (2015). In vitro evaluation of the cellular effect of indium tin oxide nanoparticles using the human lung adenocarcinoma A549 cells. Metallomics.

[523] Olgun N.S., Morris A.M., Barber T.L., Stefaniak A.B., Kashon M.L., Schwegler-Berry D., Cummings K.J., Leonard S.S. (2017). Comparison of the toxicity of sintered and unsintered indium-tin oxide particles in murine macrophage and epidermal cells. Toxicol. Appl. Pharmacol..

[524] Ng A.M., Guo M.Y., Leung Y.H., Chan C.M., Wong S.W., Yung M.M., Ma A.P., Djurisic A.B., Leung F.C., Leung K.M. (2015). Metal oxide nanoparticles with low toxicity. J Photochem. Photobiol. B.

[525] Swarts N. (2018). Material safety data sheet (MSDS): Indium tin oxide. Indium Corporation of America.

[526] Crosera M., Bovenzi M., Maina G., Adami G., Zanette C., Florio C., Filon Larese F. (2009). Nanoparticle dermal absorption and toxicity: A review of the literature. Int. Arch. Occup. Environ. Health.

[527] Larese F.F., D’Agostin F., Crosera M., Adami G., Renzi N., Bovenzi M., Maina G. (2009). Human skin penetration of silver nanoparticles through intact and damaged skin. Toxicology.

[528] Tak Y.K., Pal S., Naoghare P.K., Rangasamy S., Song J.M. (2015). Shape-Dependent Skin Penetration of Silver Nanoparticles: Does It Really Matter?. Sci. Rep..

[529] Tao W., Kong N., Ji X., Zhang Y., Sharma A., Ouyang J., Qi B., Wang J., Xie N., Kang C. (2019). Emerging two-dimensional monoelemental materials (Xenes) for biomedical applications. Chem. Soc. Rev..

[530] Wan S., Zhu Z., Yin K., Su S., Bi H., Xu T., Zhang H., Shi Z., He L., Sun L. (2018). A Highly skin-conformal and biodegradable graphene-based strain sensor. Small Methods.

[531] Liu Y., Tao L.-Q., Wang D.-Y., Zhang T.-Y., Yang Y., Ren T.-L. (2017). Flexible, highly sensitive pressure sensor with a wide range based on graphene-silk network structure. Appl. Phys. Lett..

[532] Tanpichai S., Witayakran S., Srimarut Y., Woraprayote W., Malila Y. (2019). Porosity, density and mechanical properties of the paper of steam exploded bamboo microfibers controlled by nanofibrillated cellulose. J. Mater. Res. Technol..

[533] Wang L., Lou Z., Jiang K., Shen G. (2019). Bio-Multifunctional Smart Wearable Sensors for Medical Devices. Adv. Intell. Syst..

[534] González I., Alcalà M., Chinga-Carrasco G., Vilaseca F., Boufi S., Mutjé P. (2014). From paper to nanopaper: Evolution of mechanical and physical properties. Cellulose.

[535] Petritz A., Wolfberger A., Fian A., Griesser T., Irimia-Vladu M., Stadlober B. (2015). Cellulose-derivative-based gate dielectric for high-performance organic complementary inverters. Adv. Mater..

[536] Koga H., Nagashima K., Huang Y., Zhang G., Wang C., Takahashi T., Inoue A., Yan H., Kanai M., He Y. (2019). Paper-based disposable molecular sensor constructed from oxide nanowires, cellulose nanofibers, and pencil-drawn electrodes. ACS Appl. Mater. Interfaces.

[537] Farah S., Anderson D.G., Langer R. (2016). Physical and mechanical properties of PLA, and their functions in widespread applications - A comprehensive review. Adv. Drug Deliv. Rev..

[538] Vaicekauskaite J., Mazurek P., Vudayagiri S., Skov A.L. (2020). Mapping the mechanical and electrical properties of commercial silicone elastomer formulations for stretchable transducers. J. Mater. Chem. C.

[539] Wen Z., Yang J., Ding H., Zhang W., Wu D., Xu J., Shi Z., Xu T., Tian Y., Li X. (2018). Ultra-highly sensitive, low hysteretic and flexible pressure sensor based on porous MWCNTs/Ecoflex elastomer composites. J. Mater. Sci. Mater. Electron..

[540] Baek S., Jang H., Kim S.Y., Jeong H., Han S., Jang Y., Kim D.H., Lee H.S. (2017). Flexible piezocapacitive sensors based on wrinkled microstructures: Toward low-cost fabrication of pressure sensors over large areas. RSC Adv..

[541] Cholleti E.R., Stringer J., Assadian M., Battmann V., Bowen C., Aw K. (2018). Highly stretchable capacitive sensor with printed carbon black electrodes on barium titanate elastomer composite. Sensors.

[542] Evlashin S., Dyakonov P., Tarkhov M., Dagesyan S., Rodionov S., Shpichka A., Kostenko M., Konev S., Sergeichev I., Timashev P. (2019). Flexible Polycaprolactone and Polycaprolactone/Graphene Scaffolds for Tissue Engineering. Materials.

[543] Khalid M.A.U., Ali M., Soomro A.M., Kim S.W., Kim H.B., Lee B.-G., Choi K.H. (2019). A highly sensitive biodegradable pressure sensor based on nanofibrous dielectric. Sens. Actuators A Phys..

[544] Cameron R.E., Kamvari-Moghaddam A., Jenkins M., Stamboulis A. (2012). Synthetic bioresorbable polymers. Durability and Reliability of Medical Polymers.

[545] Kalenov D.S., Meriakri V.V., Parkhomenko M.P., Zhou S. (2012). Dielectric properties of biocompatible and biodegradable polycaprolone and polylactide and their nanocomposites in the millimeter wave band. AIP Conf. Proc..

[546] Kim T.K., Kim J.K., Jeong O.C. (2011). Measurement of nonlinear mechanical properties of PDMS elastomer. Microelectron. Eng..

[547] Peterson S.L., McDonald A., Gourley P.L., Sasaki D.Y. (2005). Poly(dimethylsiloxane) thin films as biocompatible coatings for microfluidic devices: Cell culture and flow studies with glial cells. J. Biomed. Mater. Res. A.

[548] Liu X., Zhu Y., Nomani M.W., Wen X., Hsia T.-Y., Koley G. (2013). A highly sensitive pressure sensor using a Au-patterned polydimethylsiloxane membrane for biosensing applications. J. Micromech. Microeng..

[549] Cui J., Zhang B., Duan J., Guo H., Tang J. (2016). Flexible pressure sensor with ag wrinkled electrodes based on PDMS substrate. Sensors.

[550] Hou X., Zhang S., Yu J., Cui M., He J., Li L., Wang X., Chou X. (2020). Flexible piezoelectric nanofibers/polydimethylsiloxane-based pressure sensor for self-powered human motion monitoring. Energy Technol..

[551] Chen J., Guo H., He X., Liu G., Xi Y., Shi H., Hu C. (2016). Enhancing performance of triboelectric nanogenerator by filling high dielectric nanoparticles into sponge PDMS film. ACS Appl. Mater. Interfaces.

[552] Kotsilkova R., Borovanska I., Todorov P., Ivanov E., Menseidov D., Chakraborty S., Bhattacharjee C. (2018). tensile and surface mechanical properties of polyethersulphone (PES) and polyvinylidene fluoride (PVDF) membranes. J. Theor. Appl. Mech..

[553] Azadbakht M., Madaeni S.S., Sahebjamee F. (2011). Biocompatibility of polyethersulfone membranes for cell culture systems. Eng. Life Sci..

[554] Gan Z., Abe H., Doi Y. (2000). Biodegradable poly(ethylene succinate) (PES). 1. Crystal growth kinetics and morphology. Biomacromolecules.

[555] Hu L., Zhong J., Tian Y., Zheng X., Cheng J., Pu Z. (2018). Study on properties of barium titanate/polyethersulfone dielectric composites prepared by physical dispersion method. J. Mater. Sci.: Mater. Electron..

[556] Dardmeh N., Khosrowshahi A., Almasi H., Zandi M. (2017). Study on effect of the polyethylene terephthalate/nanoclay nanocomposite film on the migration of terephthalic acid into the yoghurt drinks simulant. J. Food Process Eng..

[557] Samavedi S., Poindexter L.K., Van Dyke M., Goldstein A.S., Orlando G., Lerut J., Soker S., Stratta R.J. (2014). Synthetic Biomaterials for Regenerative Medicine Applications. Regenerative Medicine Applications in Organ Transplantation.

[558] Loh K.J., Lynch J.P., Shim B.S., Kotov N.A. (2007). Tailoring piezoresistive sensitivity of multilayer carbon nanotube composite strain sensors. J. Intell. Mater. Syst. Struct..

[559] Yoon J.I., Choi K.S., Chang S.P. (2017). A novel means of fabricating microporous structures for the dielectric layers of capacitive pressure sensor. Microelectron. Eng..

[560] Yaqoob U., Phan D.-T., Uddin A.S.M.I., Chung G.-S. (2015). Highly flexible room temperature NO2 sensor based on MWCNTs-WO3 nanoparticles hybrid on a PET substrate. Sens. Actuators B Chem..

[561] Sundriyal P., Bhattacharya S., Bhattacharya S., Agarwal A.K., Chanda N., Pandey A., Sen A.K. (2018). Inkjet-printed sensors on flexible substrates. Environmental, Chemical and Medical Sensors.

[562] Dai W., Yu J., Wang Y., Song Y., Bai H., Nishimura K., Liao H., Jiang N. (2014). Enhanced thermal and mechanical properties of polyimide/graphene composites. Macromol. Res..

[563] Constantin C.P., Aflori M., Damian R.F., Rusu R.D. (2019). Biocompatibility of polyimides: A mini-review. Materials.

[564] Lin R.-H., Wang W.-M., Chen Y.-H., Ho T.-H. (2012). Preparation and characterization of biodegradable condensation polyimide. Polym. Degrad. Stab..

[565] Han T., Nag A., Afsarimanesh N., Akhter F., Liu H., Sapra S., Mukhopadhyay S., Xu Y. (2019). Gold/polyimide-based resistive strain sensors. Electronics.

[566] Makadia H.K., Siegel S.J. (2011). Poly Lactic-co-Glycolic Acid (PLGA) as Biodegradable Controlled Drug Delivery Carrier. Polymers.

[567] Karimi A., Navidbakhsh M. (2013). Mechanical properties of PVA material for tissue engineering applications. Mater. Technol..

[568] Paradossi G., Cavalieri F., Chiessi E., Spagnoli C., Cowman M.K. (2003). Poly (vinyl alcohol) as versatile biomaterial for potential biomedical applications. J. Mater. Sci. Mater. Med..

[569] Chiellini E., Corti A., D’Antone S., Solaro R. (2003). Biodegradation of poly (vinyl alcohol) based materials. Progress Polym. Sci..

[570] Nawaz A., Hümmelgen I.A. (2019). Poly (vinyl alcohol) gate dielectric in organic field-effect transistors. J. Mater. Sci. Mater. Electron..

[571] Cheung H.-Y., Lau K.-T., Ho M.-P., Mosallam A. (2009). Study on the mechanical properties of different silkworm silk fibers. J. Compos. Mater..

[572] Zhu Z., Ling S., Yeo J., Zhao S., Tozzi L., Buehler M.J., Omenetto F., Li C., Kaplan D.L. (2018). High-strength, durable all-silk fibroin hydrogels with versatile processability toward multifunctional applications. Adv. Funct. Mater..

[573] Brenckle M.A., Partlow B., Tao H., Applegate M.B., Reeves A., Paquette M., Marelli B., Kaplan D.L., Omenetto F.G. (2016). Methods and Applications of Multilayer Silk Fibroin Laminates Based on Spatially Controlled Welding in Protein Films. Adv. Funct. Mater..

[574] Wu R., Ma L., Hou C., Meng Z., Guo W., Yu W., Yu R., Hu F., Liu X.Y. (2019). silk composite electronic textile sensor for high space precision 2d combo temperature-pressure sensing. Small.

[575] He F., You X., Gong H., Yang Y., Bai T., Wang W., Guo W., Liu X., Ye M. (2020). Stretchable, biocompatible, and multifunctional silk fibroin-based hydrogels toward wearable strain/pressure sensors and triboelectric nanogenerators. ACS Appl. Mater. Interfaces.

[576] Wang C.H., Hsieh C.Y., Hwang J.C. (2011). Flexible organic thin-film transistors with silk fibroin as the gate dielectric. Adv. Mater..

[577] Mannoor M.S., Tao H., Clayton J.D., Sengupta A., Kaplan D.L., Naik R.R., Verma N., Omenetto F.G., McAlpine M.C. (2012). Graphene-based wireless bacteria detection on tooth enamel. Nat. Commun..

[578] Gao J., Li K., Xu J., Zhang W., Ma J., Liu L., Sun Y., Zhang H., Li K. (2018). Unexpected Rheological Behavior of a Hydrophobic Associative Shellac-Based Oligomeric Food Thickener. J. Agric. Food Chem..

[579] Leick S., Kott M., Degen P., Henning S., Pasler T., Suter D., Rehage H. (2011). Mechanical properties of liquid-filled shellac composite capsules. Phys. Chem. Chem. Phys..

[580] Irimia-Vladu M., Głowacki E.D., Schwabegger G., Leonat L., Akpinar H.Z., Sitter H., Bauer S., Sariciftci N.S. (2013). Natural resin shellac as a substrate and a dielectric layer for organic field-effect transistors. Green Chem..

[581] Baek S.W., Ha J.W., Yoon M., Hwang D.H., Lee J. (2018). Shellac films as a natural dielectric layer for enhanced electron transport in polymer field-effect transistors. ACS Appl. Mater. Interfaces.

[582] Irimia-Vladu M., Troshin P.A., Reisinger M., Shmygleva L., Kanbur Y., Schwabegger G., Bodea M., Schwödiauer R., Mumyatov A., Fergus J.W. (2010). Biocompatible and Biodegradable Materials for Organic Field-Effect Transistors. Adv. Funct. Mater..

[583] Kim S.J., Lee D.S., Kim I.G., Sohn D.W., Park J.Y., Choi B.K., Kim S.W. (2012). Evaluation of the biocompatibility of a coating material for an implantable bladder volume sensor. Kaohsiung J. Med. Sci..

[584] Park S., Mondal K., Treadway R.M., Kumar V., Ma S., Holbery J.D., Dickey M.D. (2018). Silicones for Stretchable and Durable Soft Devices: Beyond Sylgard-184. ACS Appl. Mater. Interfaces.

[585] Yun Y.S., Kim D.H., Kim B., Park H.H., Jin H.-J. (2012). Transparent conducting films based on graphene oxide/silver nanowire hybrids with high flexibility. Synth. Metals.

[586] Pauksch L., Hartmann S., Rohnke M., Szalay G., Alt V., Schnettler R., Lips K.S. (2014). Biocompatibility of silver nanoparticles and silver ions in primary human mesenchymal stem cells and osteoblasts. Acta Biomater..

[587] Jung J., Cho H., Yuksel R., Kim D., Lee H., Kwon J., Lee P., Yeo J., Hong S., Unalan H.E. (2019). Stretchable/flexible silver nanowire Electrodes for energy device applications. Nanoscale.

[588] Cheng Y., Wang R., Zhai H., Sun J. (2017). Stretchable electronic skin based on silver nanowire composite fiber electrodes for sensing pressure, proximity, and multidirectional strain. Nanoscale.

[589] Popov A.M., Lozovik Y.E., Fiorito S., Yahia L. (2007). Biocompatibility and applications of carbon nanotubes in medical nanorobots. Int. J. Nanomed..

[590] Simon J., Flahaut E., Golzio M. (2019). Overview of carbon nanotubes for biomedical applications. Materials.

[591] Zhang J., Xu L., Zhou B., Zhu Y., Jiang X. (2018). The pristine graphene produced by liquid exfoliation of graphite in mixed solvent and its application to determination of dopamine. J. Colloid Interface Sci..

[592] Mitra S., Banerjee S., Datta A., Chakravorty D. (2016). A brief review on graphene/inorganic nanostructure composites: Materials for the future. Ind. J. Phys..

[593] Huang H., Su S., Wu N., Wan H., Wan S., Bi H., Sun L. (2019). Graphene-based sensors for human health monitoring. Front. Chem..

[594] Pena-Bahamonde J., Nguyen H.N., Fanourakis S.K., Rodrigues D.F. (2018). Recent advances in graphene-based biosensor technology with applications in life sciences. J. Nanobiotechnol..

[595] Ali A., Pothu R., Siyal S.H., Phulpoto S., Sajjad M., Thebo K.H. (2019). Graphene-based membranes for CO_2_ separation. Mater. Sci. Energy Technol..

[596] El Beqqali O., Zorkani I., Rogemond F., Chermette H., Chaabane R.B., Gamoudi M., Guillaud G. (1997). Electrical properties of molybdenum disulfide MoS2. Experimental study and density functional calculation results. Synth. Metals.

[597] Shah P., Narayanan T.N., Li C.Z., Alwarappan S. (2015). Probing the biocompatibility of MoS2 nanosheets by cytotoxicity assay and electrical impedance spectroscopy. Nanotechnology.

[598] Yang T., Xiang H., Qin C., Liu Y., Zhao X., Liu H., Li H., Ouzounian M., Hong G., Chen H. (2019). Highly sensitive 1T-MOS pressure sensor with wide linearity based on hierarchical microstructures of leaf vein as spacer. Adv. Electron. Mater..

[599] Yi N., Abidian M.R., Poole-Warren L., Martens P., Green R. (2016). Conducting polymers and their biomedical applications. Biosynthetic Polymers for Medical Applications.

[600] Humpolicek P., Kasparkova V., Pachernik J., Stejskal J., Bober P., Capakova Z., Radaszkiewicz K.A., Junkar I., Lehocky M. (2018). The biocompatibility of polyaniline and polypyrrole: A comparative study of their cytotoxicity, embryotoxicity and impurity profile. Mater. Sci. Eng. C Mater. Biol. Appl..

[601] Ziadan K.M., Saadon W.T. (2012). Study of the electrical characteristics of polyaniline prepeared by electrochemical polymerization. Energy Proc..

[602] He X.X., Li J.T., Jia X.S., Tong L., Wang X.X., Zhang J., Zheng J., Ning X., Long Y.Z. (2017). facile fabrication of multi-hierarchical porous polyaniline composite as pressure sensor and gas sensor with adjustable sensitivity. Nanoscale Res. Lett..

[603] Liu K., Zhou Z., Yan X., Meng X., Tang H., Qu K., Gao Y., Li Y., Yu J., Li L. (2019). Polyaniline nanofiber wrapped fabric for high performance flexible pressure sensors. Polymers.

[604] Wang X., Zhang X., Sun L., Lee D., Lee S., Wang M., Zhao J., Shao-Horn Y., Dinca M., Palacios T. (2018). High electrical conductivity and carrier mobility in oCVD PEDOT thin films by engineered crystallization and acid treatment. Sci. Adv..

[605] Asplund M., Thaning E., Lundberg J., Sandberg-Nordqvist A.C., Kostyszyn B., Inganas O., von Holst H. (2009). Toxicity evaluation of PEDOT/biomolecular composites intended for neural communication electrodes. Biomed. Mater..

[606] Alemu D., Wei H.-Y., Ho K.-C., Chu C.-W. (2012). Highly conductive PEDOT: PSS electrode by simple film treatment with methanol for ITO-free polymer solar cells. Energy Environ. Sci..

[607] Stöcker T., Köhler A., Moos R. (2012). Why does the electrical conductivity in PEDOT: PSS decrease with PSS content? A study combining thermoelectric measurements with impedance spectroscopy. J. Polym. Sci. Part B Polym. Phys..

[608] Tessarolo M., Possanzini L., Campari E.G., Bonfiglioli R., Violante F.S., Bonfiglio A., Fraboni B. (2018). Adaptable pressure textile sensors based on a conductive polymer. Flex. Print. Electron..

[609] Miao J., Liu H., Li Y., Zhang X. (2018). Biodegradable Transparent Substrate Based on Edible Starch-Chitosan Embedded with Nature-Inspired Three-Dimensionally Interconnected Conductive Nanocomposites for Wearable Green Electronics. ACS Appl. Mater. Interfaces.

[610] Lv B., Chen X., Liu C. (2020). A Highly sensitive piezoresistive pressure sensor based on graphene oxide/polypyrrole @ polyurethane sponge. Sensors.

[611] Li Y., Jiang C., Han W. (2020). Extending the pressure sensing range of porous polypyrrole with multiscale microstructures. Nanoscale.

[612] Wang P., Xiong P., Liu J., Gao S., Xi T., Cheng Y. (2018). A silk-based coating containing GREDVY peptide and heparin on Mg–Zn–Y–Nd alloy: Improved corrosion resistance, hemocompatibility and endothelialization. J. Mater. Chem. B.

[613] Liu X., Gao C., Sangwan P., Yu L., Tong Z. (2014). Accelerating the degradation of polyolefins through additives and blending. J. Appl. Polym. Sci..

[614] Cheng M.Y., Lin C.L., Lai Y.T., Yang Y.J. (2010). A polymer-based capacitive sensing array for normal and shear force measurement. Sensors.

[615] Nie B., Huang R., Yao T., Zhang Y., Miao Y., Liu C., Liu J., Chen X. (2019). Textile-Based wireless pressure sensor array for human-interactive sensing. Adv. Funct. Mater..

[616] Tian X., Lee P.M., Tan Y.J., Wu T.L.Y., Yao H., Zhang M., Li Z., Ng K.A., Tee B.C.K., Ho J.S. (2019). Wireless body sensor networks based on metamaterial textiles. Nat. Electron..

[617] Chen S., Wu N., Lin S., Duan J., Xu Z., Pan Y., Zhang H., Xu Z., Huang L., Hu B. (2020). Hierarchical elastomer tuned self-powered pressure sensor for wearable multifunctional cardiovascular electronics. Nano Energy.

[618] Lee J.S., Shin K.Y., Cheong O.J., Kim J.H., Jang J. (2015). Highly sensitive and multifunctional tactile sensor using free-standing ZnO/PVDF thin film with graphene electrodes for pressure and temperature monitoring. Sci. Rep..

[619] Kim D.I., Trung T.Q., Hwang B.U., Kim J.S., Jeon S., Bae J., Park J.J., Lee N.E. (2015). A Sensor array using multi-functional field-effect transistors with ultrahigh sensitivity and precision for bio-monitoring. Sci. Rep..

[620] Fan W., He Q., Meng K., Tan X., Zhou Z., Zhang G., Yang J., Wang Z.L. (2020). Machine-knitted washable sensor array textile for precise epidermal physiological signal monitoring. Sci. Adv..

[621] He J., Xiao P., Lu W., Shi J., Zhang L., Liang Y., Pan C., Kuo S.-W., Chen T. (2019). A Universal high accuracy wearable pulse monitoring system via high sensitivity and large linearity graphene pressure sensor. Nano Energy.

